# Development of Half-Sandwich Ru, Os, Rh, and Ir Complexes Bearing the Pyridine-2-ylmethanimine Bidentate Ligand Derived from 7-Chloroquinazolin-4(3H)-one with Enhanced Antiproliferative Activity

**DOI:** 10.1021/acsomega.3c10482

**Published:** 2024-04-13

**Authors:** Michał Łomzik, Andrzej Błauż, Daniel Tchoń, Anna Makal, Błażej Rychlik, Damian Plażuk

**Affiliations:** † Faculty of Chemistry, Department of Organic Chemistry, University of Lodz, ul. Tamka 12, 91-403 Łódź, Poland; ‡ Faculty of Biology and Environmental Protection, Department of Oncobiology and Epigenetics, Cytometry Lab, University of Lodz, ul. Pomorska 141/143, 90-236 Łódź, Poland; § Laboratory for Structural and Biochemical Research (LBSBio), Biological and Chemical Research Centre, Department of Chemistry, University of Warsaw, ul. Zwirki i Wigury 101, 02-089 Warszawa, Poland; ∥ Molecular Biophysics and Integrated Bioimaging Division, Lawrence Berkeley National Laboratory, Berkeley, California 94720, United States

## Abstract

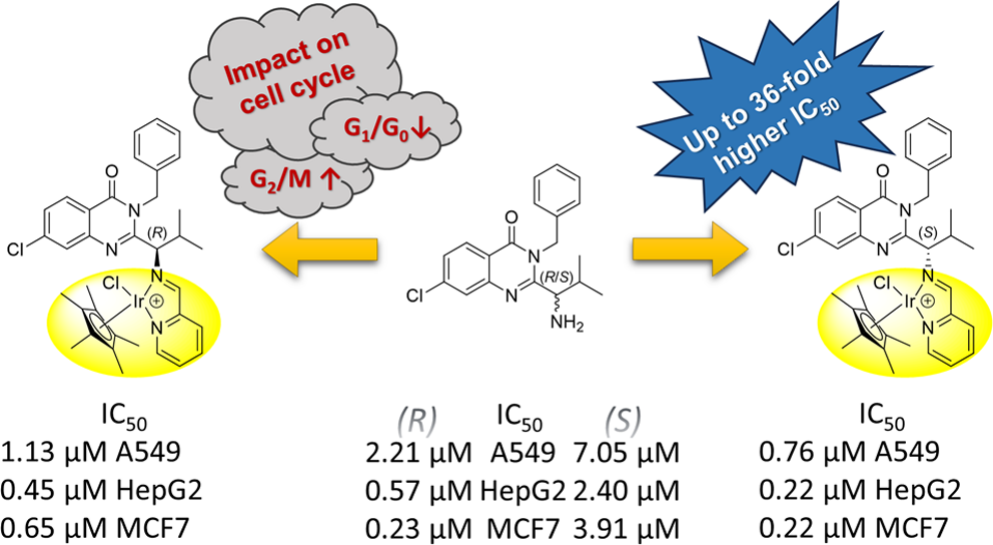

Kinesin spindle protein (KSP) inhibitors are one of the most promising anticancer agents developed in recent years. Herein, we report the synthesis of ispinesib-core pyridine derivative conjugates, which are potent KSP inhibitors, with half-sandwich complexes of ruthenium, osmium, rhodium, and iridium. Conjugation of 7-chloroquinazolin-4(3H)-one with the pyridine-2-ylmethylimine group and the organometallic moiety resulted in up to a 36-fold increased cytotoxicity with IC_50_ values in the micromolar and nanomolar range also toward drug-resistant cells. All studied conjugates increased the percentage of cells in the G_2_/M phase, simultaneously decreasing the number of cells in the G_1_/G_0_ phase, suggesting mitotic arrest. Additionally, ruthenium derivatives were able to generate reactive oxygen species (ROS); however, no significant influence of the organometallic moiety on KSP inhibition was observed, which suggests that conjugation of a KSP inhibitor with the organometallic moiety modulates its mechanism of action.

## Introduction

Despite the recent development of many cancer treatments, chemotherapy remains the primary, and often the only, method used.^1−3^ Among the numerous anticancer drugs, antimitotic compounds such as taxanes and *Vinca* alkaloids are the most important.^2,4^ Antimitotic agents such as taxanes disrupt the typical microtubule dynamics, leading to cancer cell death but can also cause many side effects, such as bleeding, immune system impairment, reduced blood pressure, and pain in muscles and joints.^5−7^ Additionally, the multidrug resistance phenomenon can be observed during chemotherapy, thus decreasing its efficiency. Therefore, developing new molecules able to overcome the drawbacks of currently used antimitotic compounds is still essential.

In the last years, low-molecular-weight inhibitors of the kinesin spindle protein (KSP) were developed.^5^ The KSP is a member of the motor protein family and plays a crucial role in spindle pole separation. It is highly active in dividing cells, while its activity is almost undetectable in nondividing cells.^8^ KSP inhibitors disturb the mitosis without direct microtubule disruption.^8−10^ Numerous KSP inhibitors have been developed, including monastrol,^11^ dimethylenastron,^8^ ispinesib (SB-715992), SB-743921,^8,12^ litronesib (LY2523355),^13^ MK-0731,^14^ and filanesib (ARRY-520).^15,16^ Some of these compounds have been clinically tested in at least 45 phase I/II trials against various types of cancer,^13^ with ispinesib^12,17^ and filanesib^15,18,19^ as the most promising candidates. Encouraging results of clinical trials of ispinesib use in patients with metastatic or relapsing squamous cell carcinoma of the head and neck, with no signs of disease progression or intolerable toxicity, were observed within 21 days of the first dose;^20^ however, up to date, no further phase III studies have been reported.

One of the fruitful methods to develop new anticancer drug candidates involves constructing conjugates of active compounds with an organometallic group.^21−23^ The most intensively studied organometallic derivatives include metallocenes^24^ (mainly ferrocene and ruthenocene) and half-sandwich complexes of ruthenium,^25−28^ osmium,^26,29^ rhodium,^30^ and iridium.^30^ Organometallic compounds have several advantages over purely organic molecules. The presence of an organometallic moiety can increase the affinity to the biological targets by allowing the formation of new hydrophobic or metal–organic interactions with the protein. Organometallic compounds often have access to a protein binding site that is inaccessible to organic molecules. In addition, the presence of a metal atom often increases the ability of the compound to generate reactive oxygen species (ROS), which can induce apoptosis. Organometallic conjugates often exhibit stronger antiproliferative properties than parent compounds and, in many cases, exhibit additional biological properties. In recent years, many new organometallic conjugates of antimitotic compounds have been developed, including derivatives of curcumin,^31,32^ taxanes,^33,34^ colchicine,^35−37^ ethacrynic acid,^38,39^ paullone,^40^ or podophyllotoxin.^41,42^ The resulting conjugates demonstrate a higher antiproliferative activity or a new mechanism of action, being highly selective against tumor cells.

**Figure 1:**
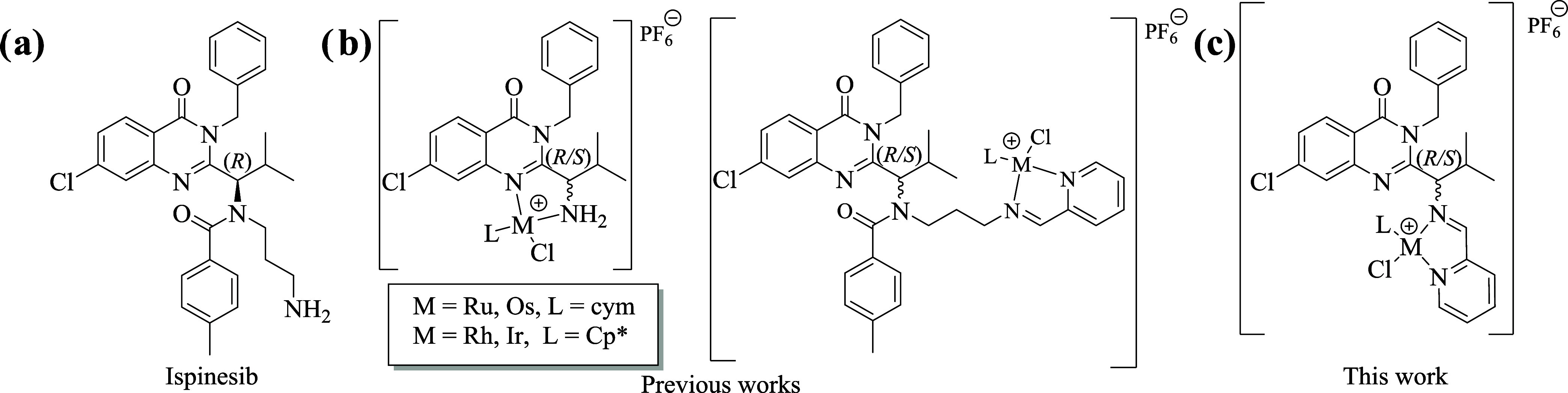
Structure of (a) ispinesib, (b) its quinazoline-derived Ru, Os, Rh, and Ir half-sandwich conjugates reported previously,^44,45^ and (c) compounds studied herein.

Recently, we have reported the synthesis and biological evaluation of a series of ferrocenyl^43^ and Ru, Os, Rh, and Ir half-sandwich^44,45^ conjugates of ispinesib and its 7-chloroquinazolin-4(3H)-one core. Continuing our study on novel organometallic antimitotic agents, we designed new half-sandwich complexes derived from the ispinesib core. Herein, we present the synthesis, structure, and biological activity studies of novel Ru, Os, Rh, and Ir half-sandwich complexes bearing the pyridine-2-ylmethanimine bidentate ligand derived from 7-chloroquinazolin-4(3H)-one (Figure 1).

## Results and Discussion

### Synthesis

**Scheme 1:**
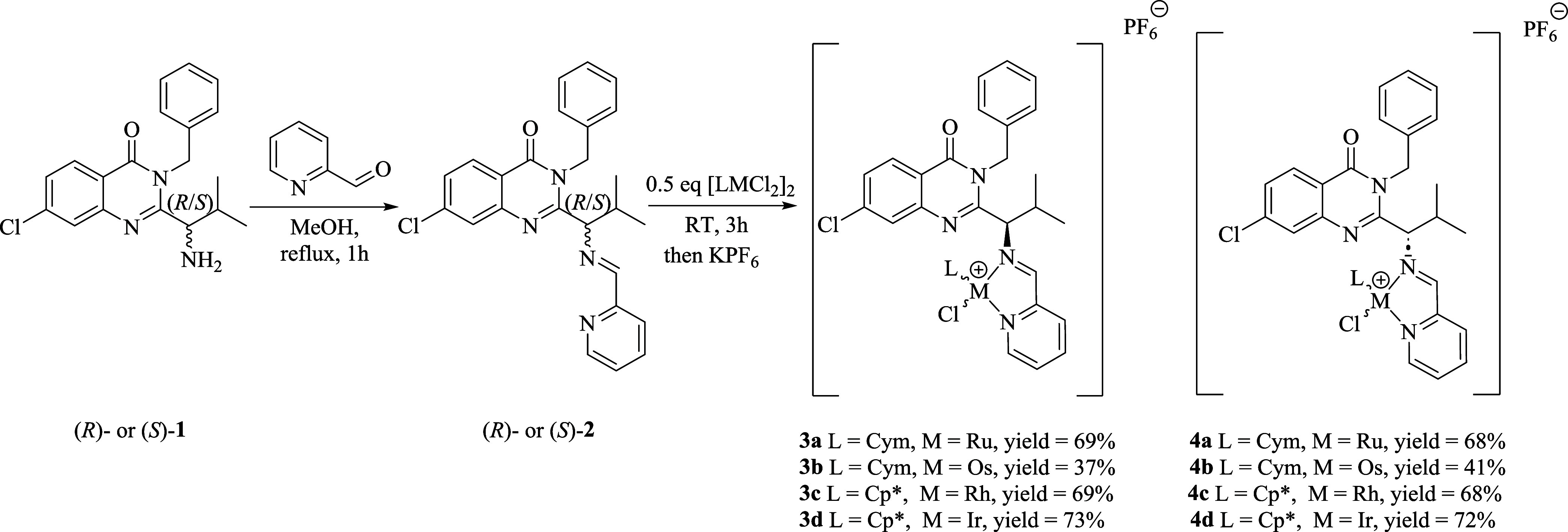
Synthesis of Complexes **3a**–**4d**

The half-sandwich complexes **3a**–**d** and **4a**–**d** were synthesized in two steps according to Scheme 1. First, (*R*)- and (*S*)-**2** imine ligands were generated in situ by reacting (*R*)- and (*S*)-**1** with 2 equiv of pyridine-2-carbaldehyde in anhydrous ethanol for 1 h. Next, 0.5 equiv of the proper dimetallic precursor [(cym)MCl_2_]_2_ (M = Ru for **3a** and **4a**, M = Os for **3b** and **4b**) or [(Cp*)MCl_2_]_2_ (M = Rh for **3c** and **4c** or M = Ir for **3d** and **4d**) was added to the reaction mixture. After 3 h of stirring at RT, the desired complexes **3a**–**d** or **4a**–**d** were isolated as hexafluorophosphate salts in 37–73% yield. All complexes were fully characterized by ^1^H and ^13^C{^1^H} NMR spectroscopy and ESI-MS analyses. The purity of compounds was confirmed by elemental analysis.

**Figure 2:**
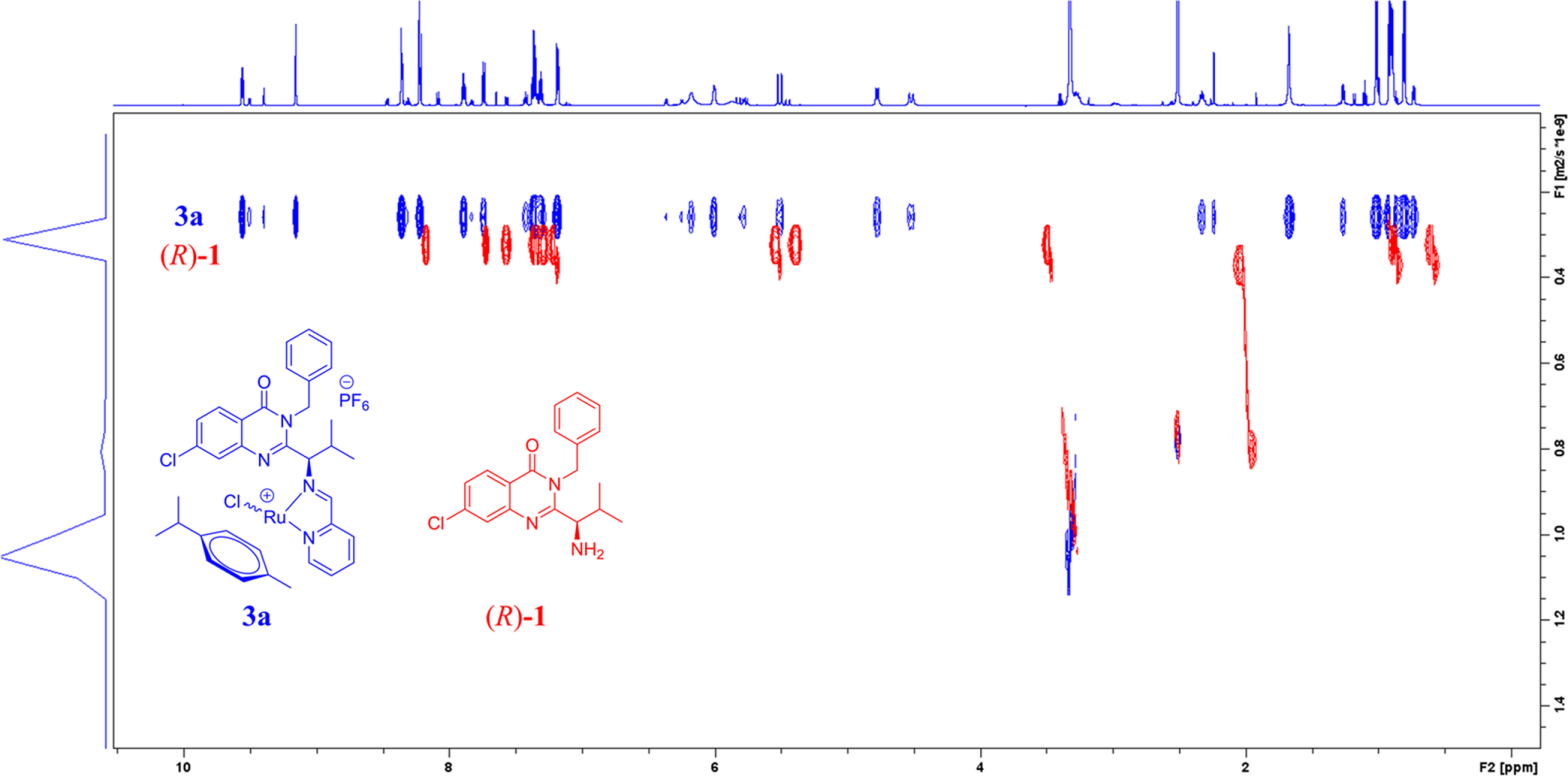
Overlapped ^1^H DOSY spectra of **3a** (blue) and (*R*)-**1** (red) in DMSO-*d*_6_.

It might be expected that a mixture of diastereoisomers of **3a**–**d** and **4a**–**d** would be formed as the result of the complexation reactions of enantiomerically pure imines **2** due to the generation of new chirality on the metal atoms. In the ^1^H NMR spectra of **3a**–**c** and **4a**–**c**, only one main set of peaks was observed in ^1^H and ^13^C{^1^H} NMR spectra together with small amounts (∼15%) of a second species, which can be assigned to the other diastereoisomers. Yet, for iridium complexes (**3d** and **4d**), we detected much more intensive signals originating from the second diastereoisomer (ratio of 1:0.4 for **3d** and ratio of 1:1 for **4d**). The formation of two diastereoisomers of the complexes was also confirmed by diffusion-ordered spectroscopy (DOSY) experiments (Figures 2 and S1–S3). For example, the DOSY spectra of **3a** and (*R*)-**1** (Figure 2) confirmed that all ^1^H signals observed in the ^1^H NMR spectra originate from the molecule(s) showing the same diffusion coefficient.

Notwithstanding, the DOSY experiments confirmed the presence in the solution of compounds showing the same diffusion coefficient. The formation of diastereoisomers of complexes **3a**–**4d** was confirmed by HPLC-MS analysis (Figures S9–S16). For example, the HPLC-MS analysis of both ruthenium complexes reveals two peaks at τ_1_ = 3.50 and τ_2_ = 5.22 min for **3a**, with a ratio of 1:5, with the *m*/*z* of 701 assigned to [M_**3a**-PF6_]^+^, and τ_1_ = 3.39 and τ_2_ = 5.07 min for **4a**, with a ratio of 1:4.3, with the *m*/*z* of 701 assigned to [M_**4a**-PF6_]^+^. Likewise, the HPLC-MS analysis of both osmium complexes **3b** and **4b** confirmed the formation of two diastereoisomers with the ratio of 1:4 (Figures S11 and S12). In the case of iridium complexes (**3d** and **4d**), the ratio of HPLC peaks is 1:0.4 for **3d** and 1:1.4 for **4d**, corresponding with the results observed in ^1^H NMR. However, for rhodium complexes (**3c** and **4c**), only the main peak at τ_1_ = 2.67 min for **3c** and τ_1_ = 2.79 min for **4c**, with an additional small peak (ratio 1:0.08), and small peaks at τ_2_ = 2.26 min for **3c** and τ_2_ = 2.40 min for **4c** with the *m*/*z* of 334 assigned to [M_**3c**-Cl-PF6_]^2+^ and [M_**4c**-Cl-PF6_]^2+^ were detected.

**Figure 3:**
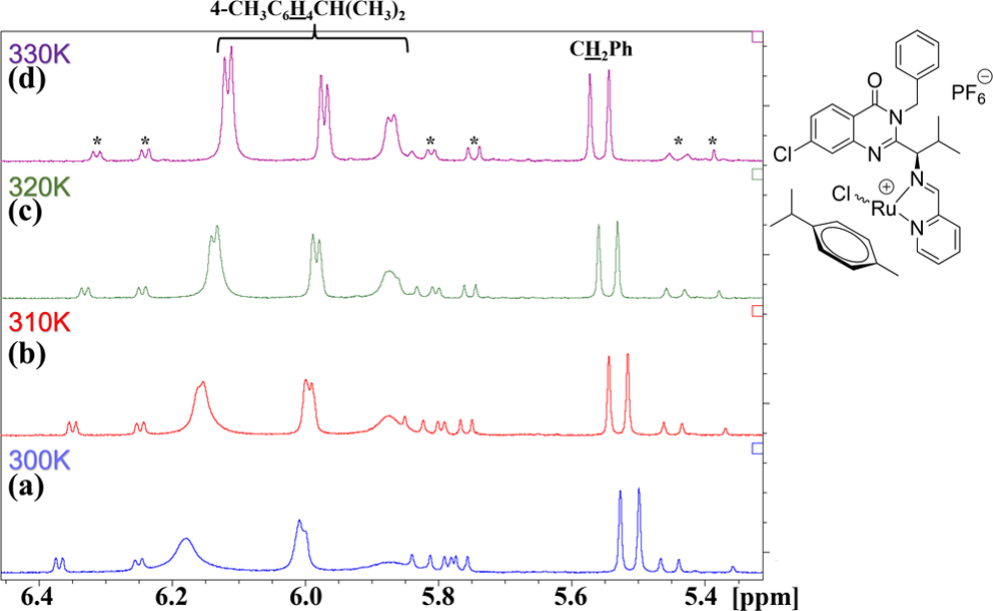
VT-NMR experiments for **3a**. ^1^H NMR spectra in DMSO-*d*_6_ (range 6.45–5.35 ppm) at (a) 300, (b) 310, (c) 320, and (d) 330 K; * denotes the signals assigned to the solvated compound.

On the ^1^H NMR spectra of complexes **3a**–**b** and **4a**–**b** at 300 K, aromatic *p*-cymene proton signals were broad singlets. Also, no correlation between aromatic *p*-cymene protons or proton–carbon correlations in ^1^H–^1^H COSY or ^1^H–^13^C HSQC NMR spectra was observed. Therefore, we performed VT-NMR experiments for **3a** and **3b** in DMSO-*d*_6_ at various temperatures between 300 and 330 K (Figures 3 and S4a–d). An increase in the temperature of the sample from 300 to 330 K results in a change of broad singlets at 6.18 and 6.00 ppm into actual doublets and a doublet at 5.87 ppm, which were assigned to aromatic *p*-cymene protons. Additionally, a small set of signals, most likely originating from the hydrolyzed form of the complex, was observed during the experiment. The ^1^H–^1^H COSY and ^1^H–^13^C HSQC spectra allowed observing the expected correlations between aromatic *p*-cymene protons and carbon atoms (Figures S5 and S6) at 330 K. However, those experiments confirmed the partial thermal decomposition of studied complexes, which impeded the performed ^13^C{^1^H} NMR spectra. Identical results were observed for **4a** and **4b** (300 and 330 K) (Figures S7, S8, S45–S47, S59–S61).

**Figure 4:**
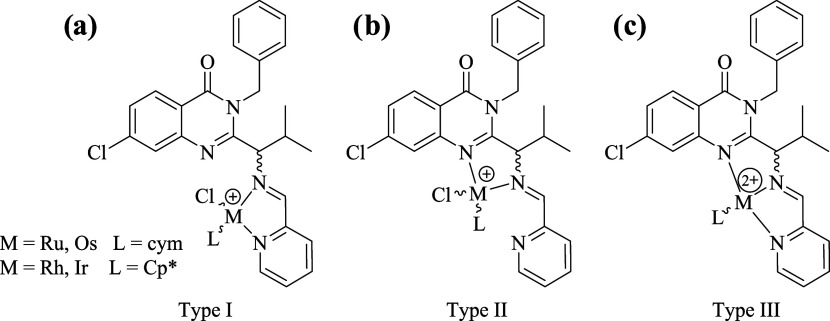
Three possible coordinations of the metal to ligand **2**. Bidentate coordination of (a) Type 1 and (b) Type II and (c) tridentate coordination of Type III.

**Scheme 2:**
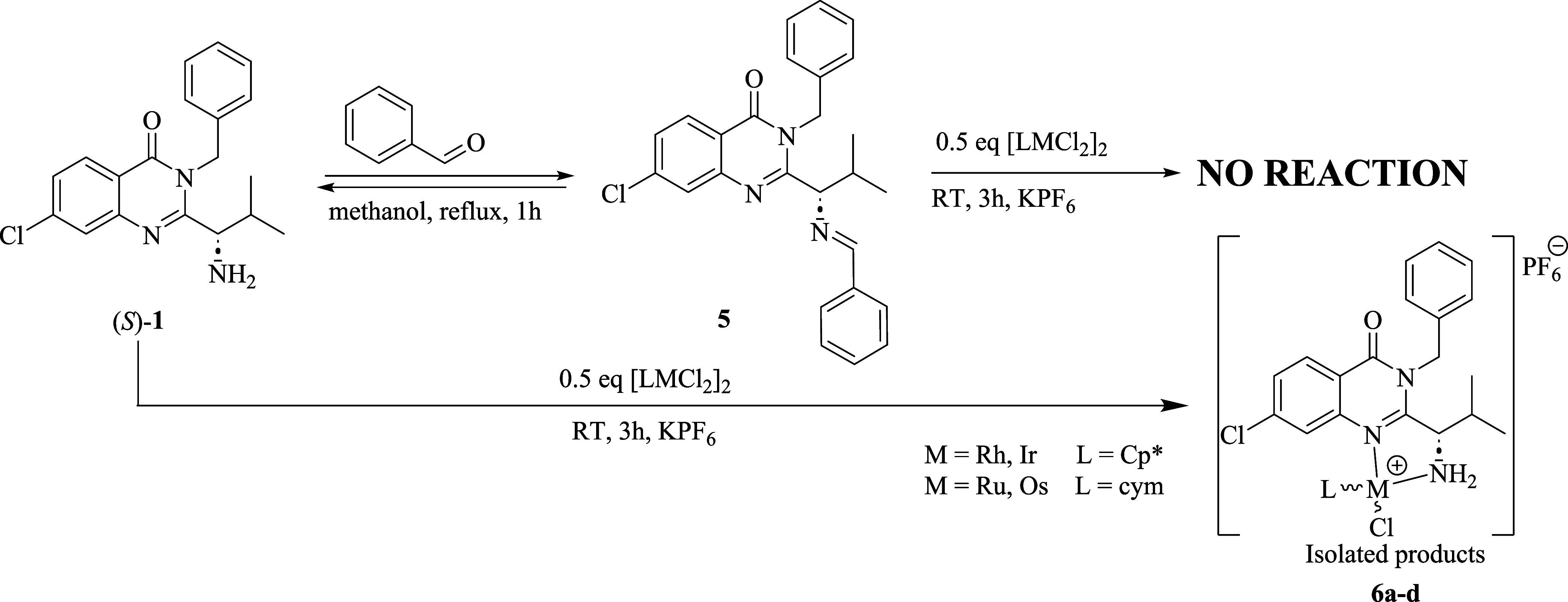
Competitive Complexation of (*S*)-**1** and **5** with [LMCl_2_]_2_

It could be expected that ligand **1** may undergo complexation forming the expected Type I complexes together with two other Type II and Type III complexes (Figure 4). The formation of Type III complexes was excluded by MS analysis. In the MS spectra of **3a**–**4d**, we observed only expected *m*/*z* values assigned to monocations [M]^+^ (Figures S9–S16). To further exclude the formation of Type II complexes, we generated imine **5** in the reaction of **1** with benzaldehyde (Scheme 2). The obtained imine **5** further reacted with 0.49 equiv of metal dimers [LMCl_2_]_2_ (M = Rh/Ir, L = Cp* or M = Ru/Os, L = cym) in methanol at RT for 3 h. After the workup, we isolated only previously reported complexes **6a**–**d** bearing **1** as *N*,*N*-bidentate ligands in trace yield. As the formation of imines is reversible, unless coordinated to a metal,^46^ imine **5** hydrolyzed in the presence of a trace of water to amine **1**, which underwent complexation with [LMCl_2_]_2_ to afford complexes **6a**–**d**. The NMR spectra of the isolated complexes were identical to those reported previously.^44^

### X-ray Diffraction Studies

Although we obtained complexes as a mixture of two possible diastereoisomers, the crystallization of **4a** from the dichloromethane/*n*-pentane mixture by slow evaporation in −20 °C allowed to isolate only one enantiopure isomer **4a**^*S,S_Ru_*^. The complex **4a**^*S,S_Ru_*^ crystallized in the P2_1_ space group and its chiral purity has been confirmed by a low value of the Flack parameter (Table S1).

**Figure 5:**
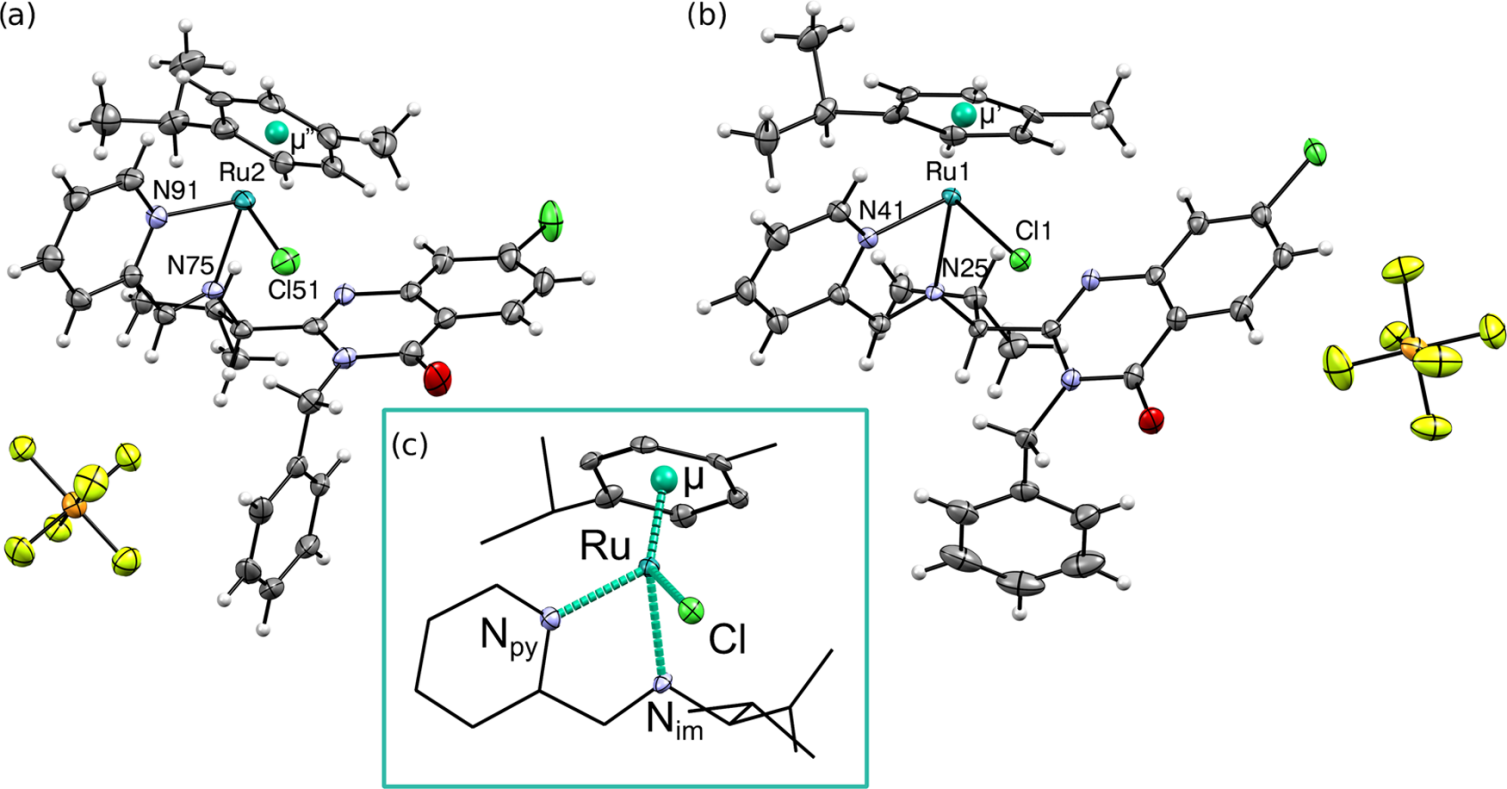
Oak Ridge thermal ellipsoid plot (ORTEP) representation of the molecular structure of **4a**^*S,S*_*Ru*_^: (a) molecule **4a**^*S,S*_*Ru*″_^ with the counterion, (b) molecule **4a**^*S,S*_*Ru*′_^ with the counterion, and (c) schematic representation of the ruthenium coordination sphere. Interatomic distances and angles reported in Table 1 are highlighted in blue. Atomic displacement parameters are drawn at the 50% probability level. Hydrogen atoms are represented as fixed-size spheres in panels (a) and (b) and omitted in panel (c). The cocrystallized disordered solvent molecule has also been removed for clarity.

The imine (*S*)-**2** acts as a *N*,*N*-bidentate ligand, forming five-membered rings with the metal ions by coordinating through the iminium and pyridinium nitrogen (Figure 5). Two similar structures of the complex are present in the unit cell, showing almost identical ruthenium coordination, varying slightly in the conformation of the terminal phenyl and ^*i*^Pr moieties. In Table 1, we had listed bond lengths of the coordination bonds for both forms, which are typical for such types of complexes.^47,48^ A more thorough description of the molecular geometry has been presented in the ESI.

**Table 1:** Selected Coordination Bond Lengths (Å) and Angles (deg) Found in Both Independent Molecules of **4a**^*S,S*_*Ru*_^ in Its Crystal Structure

bond or angle	**4a**^*S,S*_*Ru*′_^	**4a**^*S,S*_*Ru*″_^
Ru–Cl	2.396(1) Å	2.383(2) Å
Ru–N_py_	2.082(4) Å	2.096(4) Å
Ru–N_im_	2.131(4) Å	2.119(4) Å
Ru−μ *[center of the p-cymene ring]*	1.699(2) Å	1.695(2) Å
N_im_–Ru–N_py_	76.7(2)°	76.7(2)°
N_im_–Ru–Cl	86.4(1)°	85.2(1)°
N_py_–Ru–Cl	85.9(1)°	82.4(1)°
N_im_–Ru−μ	135.55°	134.16°
N_py_–Ru−μ	128.88°	132.27°
Cl–Ru−μ	127.11°	125.53°

### Stability Study

**Figure 6:**
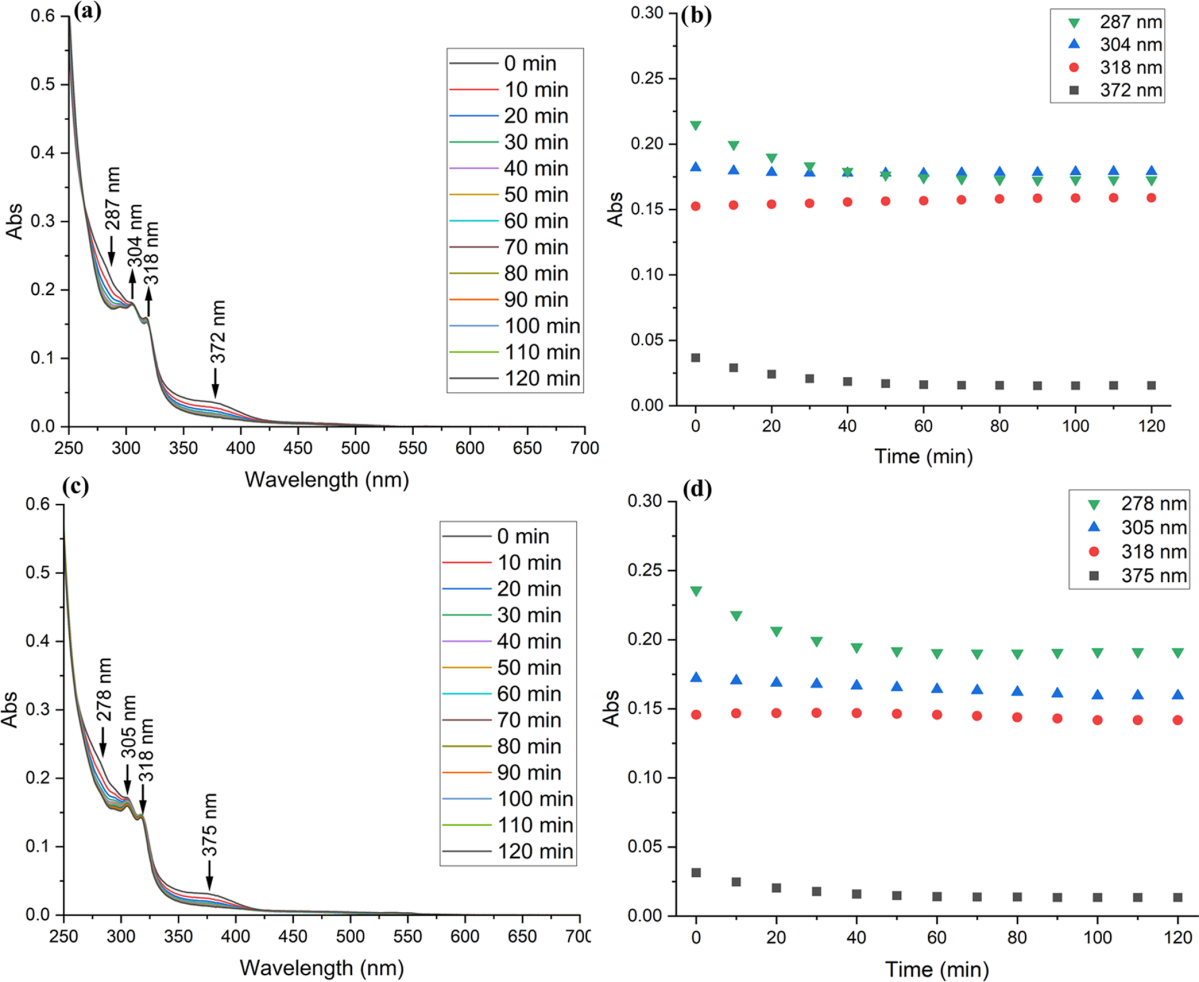
UV–vis spectra of **3d** in DMSO-water solutions in the presence of 0.2 mM (a) l-cysteine or (c) l-histidine. The absorbance maxima value changes vs time in the presence of (b) l-cysteine and (d) l-histidine.

For biological studies, compounds are commonly administered as dimethyl sulfoxide (DMSO) solution to cells cultured in a specific medium such as Dulbecco’s modified Eagle’s medium (DMEM). DMEM consists of numerous organic compounds which may act as ligands for organometallics. Therefore, it is important to know how the compounds behave in such conditions. The two most prominent components of DMEM which may coordinate to half-sandwich complexes are l-cysteine and l-histidine. Both of those amino acids are present in DMEM at 0.2 mM concentration, so we studied how the complexes interact with them using UV–vis spectroscopy and HPLC-MS analysis. The DMSO solutions of complexes were added to the aqueous solution of l-cysteine or l-histidine to achieve a complex concentration of 20 μM while keeping the DMSO concentration at 0.5 vol %. The UV–vis spectra and HPLC-MS analysis indicate that neither ruthenium **3a** nor the osmium complex **3b** reacts with those amino acids within 2 h (Figures S18–S21, S24 and S25). The rhodium complex **3c** slowly reacts with l-cysteine (Figure S22) by increasing the intensity of each absorbance maximum (λ = 279, 304, 317, 348 nm). HPLC-MS analysis confirmed the formation of an additional peak at τ = 0.95 min with *m*/*z* 714 assigned to [M-Cl-PF_6_ + HCOOH]^+^; additionally, the intensity of peaks corresponding to **3c** is lower (Figure S26). A similar effect is observed in the case of l-histidine, with an increase of only one maximum at λ = 278 nm, while the others are almost unchanged (Figure S23). On the other hand, the iridium complex **3d** reacts with both l-cysteine and l-histidine (Figure 6) in 40 min. The intensity of absorbance peaks at λ = 287 and λ = 372 nm in the presence of cysteine is decreasing, while the intensity of peaks at λ = 304 and λ = 318 nm is almost intact. HPLC-MS analysis shows that the intensity of both peaks corresponding to **3d** is lower, while the additional peak at τ = 1.09 min with *m*/*z* 804 is assigned to [M-Cl-PF_6_ + HCOOH]^+^ for the l-histidine experiment and at τ = 1.07 min with *m*/*z* 804 is assigned to [M-Cl-PF_6_ + HCOOH]^+^ for the l-cysteine experiment (Figure S27). The lack of an isosbestic point on the UV–vis spectra and HPLC-MS analysis indicate that the reaction does not lead to the dissociation of ligands **2** and is purely associated with Cl ligand exchange.

### Biological Activity

#### Antiproliferative Potential

To assess the impact of conjugating half-sandwich complexes with amines **1** via an imine-pyridine ligand on biological activity, we examined the antiproliferative potential of (*R*)- and (*S*)-**1** and organometallic conjugates **3a**–**d** and **4a**–**d** in selected human cancer cell lines: alveolar basal epithelial cell adenocarcinoma (A549), colorectal adenocarcinomas (Colo205 and SW620), colorectal carcinoma (HCT116), hepatocellular carcinoma (HepG2), and breast adenocarcinoma (MCF7). The choice of cell lines was dictated by results of previously published clinical trials on ispinesib.^49,50^ All complexes demonstrate an antiproliferative potential in the micromolar or nanomolar range (Table 2, Figures S28 and S30). The activity of these compounds varies significantly depending on the configuration of imine-ligand **2** and the cell line tested. Complexation of the imine derived from (*R*)-**1** by osmium, resulting in complex **3b**, leads to an enhanced cytotoxicity toward A549 (2-fold), HepG2 (3-fold), and MCF7 (3-fold). A similar effect is observed for Rh **3c** and Ir **3d** complexes derived from imine (*R*)-**2**, characterized by a 2-fold increased antiproliferative potential toward A549. However, the complexation of imine (*R*)-**2** with ruthenium **3a** does not enhance the activity toward studied cell lines. Nevertheless, the complexation of the imine derived from (*S*)-**1** with all metals results in a significantly increased antiproliferative potential. It is especially evident in the case of the ruthenium complex **4a** (approximately 6-fold increased activity against MCF7 and Colo205), the osmium complex **4b** (increased cytotoxicity against Colo205 (7-fold), HCT116 (10-fold), and MCF7 (9-fold)), and the iridium complex **4d** (enhanced activity toward all tested cell lines, ranging from 9- to 36-fold). Notably, the iridium complex **4d** also exhibits a significantly higher cytotoxicity compared to both (*S*)-**1** and the more cytotoxic amine (*R*)-**1** (2.6- and 1.6-fold, respectively). Additionally, within the tested concentration ranges, all of the compounds studied show no antiproliferative effects on the normal MRC-5 cell line, with IC_50_ values exceeding 100 μM (Figure S31).

**Table 2:** Antiproliferative Activity of (*R*)-**1** and (*S*)-**1** and Organometallic Complexes **3a**–**4d** in Human Cancer Cell Lines^a^

	IC_50_ [μM]					
compound	A549	Colo205	HCT116	HepG2	MCF7	SW620
(*R*)-**1**	2.21	0.107	0.346	0.566	0.231	0.096
	[1.88–2.59]	[0.094–0.121]	[0.274–0.437]	[0.476–0.672]	[0.195–0.308]	[0.080–0.117]
**3a**	2.45	1.26	2.88	1.57	0.858	1.22
	[2.05–2.93]	[1.16–1.38]	[2.48–3.43]	[1.45–1.70]	[0.742–0.988]	[1.12–1.33]
	(0.902)	(0.085)	(0.120)	(0.360)	(0.269)	(0.079)
**3b**	1.04	0.448	0.424	0.188	0.073	0.556
	[0.983–1.07]	[0.412–0.492]	[0.388–0.476]	[0.174–0.204]	[0.068–0.079]	[0.514–0.601]
	(2.12)	(0.239)	(0.816)	(3.01)	(3.16)	(0.173)
**3c**	1.16	0.138	0.173	0.689	0.357	0.152
	[0.906–1.50]	[0.125–0.153]	[0.145–0.206]	[0.605–0.784]	[0.279–0.459]	[0.125–0.185]
	(1.90)	(0.775)	(2.00)	(0.821)	(0.647)	(0.632)
**3d**	1.13	0.524	0.476	0.454	0.653	0.198
	[0.973–1.32]	[0.453–0.606]	[0.408–0.550]	[0.383–0.539]	[0.552–0.767]	[0.172–0.226]
	(1.96)	(0.204)	(0.727)	(1.25)	(0.354)	(0.485)
(*S*)-**1**	7.05	6.07	8.06	2.40	3.91	2.87
	[6.42–7.36]	[5.18–7.39]	[7.29–8.90]	[2.18–2.63]	[3.56–4.31]	[2.68–3.06]
**4a**	3.25	0.939	3.76	1.89	0.634	2.91
	[2.84–3.73]	[0.737–1.20]	[3.32–4.27]	[1.64–2.17]	[0.539–0.743]	[2.63–3.21]
	(2.17)	(6.46)	(2.14)	(1.27)	(6.17)	(0.986)
**4b**	2.19	0.904	0.823	1.13	0.438	2.31
	[2.00–2.40]	[0.825–0.985]	[0.658–1.07]	[1.04–1.23]	[0.398–0.483]	[2.18–2.45]
	(3.22)	(6.71)	(9.79)	(2.12)	(8.93)	(1.24)
**4c**	3.79	2.89	2.94	1.15	1.50	2.24
	[3.45–4.16]	[2.64–3.15]	[2.75–3.14]	[0.833–1.58]	[1.14–1.94]	[1.95–2.61]
	(1.86)	(2.10)	(2.74)	(2.09)	(2.61)	(1.28)
**4d**	0.764	0.216	0.222	0.218	0.216	0.139
	[0.613–0.954]	[0.197–0.235]	[0.192–0.254]	[0.193–0.244]	[0.191–0.244]	[0.128–0.150]
	(9.23)	(28.1)	(36.31)	(11.01)	(18.10)	(20.65)

a Exposure time 72 h; IC_50_ values are presented together with the corresponding 95% confidence intervals (in brackets), *n* = 3; the activity factors were calculated as IC_50(1)_/IC_50(**3a**–**4d**)_ and are given in parentheses below the confidence intervals.

Next, we evaluated the cytotoxicity of the synthesized complexes toward the panel of six multidrug-resistant (MDR) cell lines derived from SW620 and characterized by the overexpression of various ABC proteins, namely, ABCG2 (SW620C and SW620Mito), ABCC1 (SW620M and SW620E), and ABCB1 (SW620D, SW620E, and SW620V) (Table 3, Figures S29 and S32). Among the series of complexes bearing the (*R*)-**2** ligand, only the iridium complex **3d** shows a 2.2- and 1.7-fold higher cytotoxicity than the corresponding amine (*R*)-**1** toward SW620C and SW620D cancer cell lines. The activity of the complexes derived from the ligand (*S*)-**2** is also considerably higher than that of the compounds containing the ligand (*R*)-**2**. The cytotoxicity of both rhodium **4c** and iridium **4d** complexes is higher than that of amine (*S*)-**1**. In the case of **4c**, the increase in cytotoxicity is low, with the highest value of 3.7-fold for the SW620Mito line. Nevertheless, the IC_50_ values for the iridium complex **4d** are 6.1- to 20.6-fold lower than those for amine (*S*)-**1**. Compound **4d** also exerts a 2.1- and 2.6-fold higher cytotoxicity than (*R*)-**1** against the SW620C and SW620D lines.

**Table 3:** Antiproliferative Activity of (*R*)-**1** and (*S*)-**1** and Organometallic Complexes **3a**–**4d** in Multidrug-Resistant (MDR) Cancer Cell Lines^a^

	IC_50_ [μM]						
comp.	SW620	SW620C	SW620D	SW620E	SW620M	SW620V	SW620Mito
(*R*)-**1**	0.096	0.721	1.12	0.835	0.241	0.206	0.261
	[0.080–0.117]	[0.552–0.942]	[0.851–1.47]	[0.627–1.11]	[0.191–0.305]	[0.160–0.267]	[0.207–0.329]
**3a**	1.22	4.38	7.23	5.52	3.45	4.71	4.33
	[1.12–1.33]	[3.80–5.07]	[6.24–8.37]	[4.74–6.43]	[2.99–3.97]	[4.08–5.44]	[3.80–4.95]
	(0.079)	(0.165)	(0.155)	(0.151)	(0.070)	(0.044)	(0.060)
**3b**	0.556	1.17	3.92	3.52	0.591	1.03	0.748
	[0.514–0.601]	[1.08–1.27]	[3.60–4.27]	[3.25–3.83]	[0.522–0.667]	[0.953–1.12]	[0.683–0.815]
	(0.173)	(0.616)	(0.286)	(0.237)	(0.408)	(0.200)	(0.349)
**3c**	0.152	0.592	4.18	0.880	1.18	0.210	0.524
	[0.125–0.185]	[0.526–0.666]	[3.13–5.96]	[0.657–1.18]	[0.998–1.42]	[0.175–0.253]	[0.418–0.657]
	(0.632)	(1.22)	(0.268)	(0.949)	(0.204)	(0.981)	(0.498)
**3d**	0.198	0.335	0.644	1.02	0.247	0.267	0.268
	[0.172–0.226]	[0.297–0.377]	[0.565–0.732]	[0.928–1.13]	[0.222–0.275]	[0.224–0.317]	[0.233–0.311]
	(0.485)	(2.15)	(1.74)	(0.819)	(0.976)	(0.771)	(0.974)
(*S*)-**1**	2.87	3.33	4.15	4.05	3.46	3.44	3.91
	[2.68–3.06]	[2.94–3.78]	[3.65–4.75]	[3.57–4.61]	[3.03–3.98]	[3.00–3.94]	[3.46–4.44]
**4a**	2.91	4.36	6.01	5.46	3.24	5.01	3.68
	[2.63–3.21]	[3.90–4.88]	[5.24–6.88]	[4.77–6.26]	[2.91–3.61]	[4.43–5.58]	[3.29–4.10]
	(0.986)	(0.764)	(0.690)	(0.741)	(1.07)	(0.687)	(1.06)
**4b**	2.31	4.54	13.5	12.8	2.45	9.30	3.78
	[2.18–2.45]	[4.15–4.99]	[12.3–15.0]	[11.7–14.2]	[2.25–2.66]	[8.52–10.1]	[3.48–4.11]
	(1.24)	(0.738)	(0.307)	(0.316)	(1.41)	(0.370)	(1.03)
**4c**	2.24	2.97	6.07	6.20	2.25	2.98	1.05
	[1.95–2.61]	[2.55–3.45]	[4.88–7.85]	[4.96–8.05]	[1.95–2.59]	[2.58–3.45]	[0.878–1.25]
	(1.28)	(1.12)	(0.684)	(0.653)	(1.54)	(1.15)	(3.72)
**4d**	0.139	0.343	0.425	0.663	0.387	0.411	0.365
	[0.128–0.150]	[0.310–0.379]	[0.384–0.472]	[0.582–0.754]	[0.339–0.443]	[0.374–0.451]	[0.313–0.428]
	(20.65)	(9.71)	(9.76)	(6.11)	(8.94)	(8.37)	(10.71)

a Exposure time 72 h; IC_50_ values are presented together with the corresponding 95% confidence intervals (in brackets), *n* = 3; the activity factors were calculated as IC_50(1)_/IC_50(**3a–4d**)_ and are given in parentheses below the confidence intervals.

#### Cell Cycle

**Figure 7:**
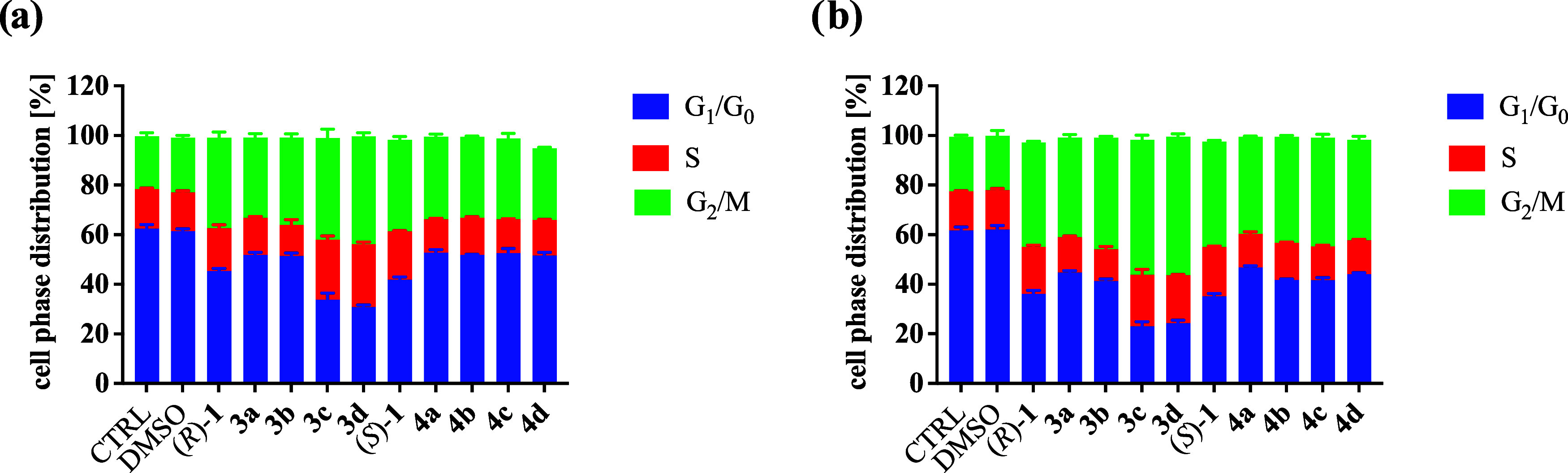
Cell cycle distribution in SW620 cells: (a) after 24 h and (b) after 48 h.

Ispinesib leads to the formation of monopolar mitotic spindles and a blockade of chromosome segregation in cancer cells. Using flow cytometry, we assessed the cell cycle distribution in the SW620 and SW620E cells exposed to the studied compounds for 24 and 48 h. Only two complexes, rhodium **3c** and iridium **3d**, exhibit a significantly different impact on cell cycle phase distribution. In contrast, all other complexes demonstrate a pattern similar to the corresponding amines (*R*)- and (*S*)-**1**, as shown in Figure 7 and Table S2. Both complexes, **3c** and **3d**, decrease the percentage of cells in the G_1_/G_0_ phase and increase the percentage in the S and G_2_/M phases. All other compounds exhibit a similar impact on cell phase distribution. Furthermore, prolonged exposure to the compounds increases the percentage of cells in the G_2_/M phase, with the most intensive effect observed for **3c** and **3d**. These results suggest an aggravated mitotic arrest in cells treated with the rhodium **3c** and iridium **3d** complexes. However, none of the studied compounds affects the cell cycle in SW620E cells, as demonstrated in Figure S33.

### KSP Inhibitory Activity

**Figure 8:**
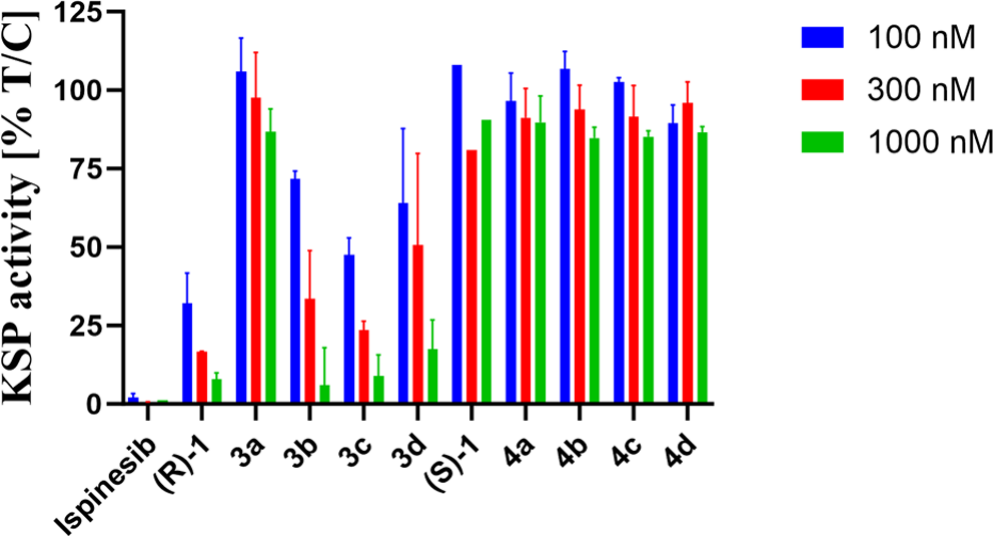
KSP activity after being treated with studied compounds at 100, 300, and 1000 nM concentrations.

The mechanism of the anticancer activity of ispinesib is related to the inhibition of the activity of the KSP. Thus, we studied the synthesized compounds’ ability to inhibit KSP activity using the adenosine 5′-triphosphate (ATP) hydrolysis assay. The inhibitory ability of the KSP is strongly correlated with the configuration of the organic ligand and the type of metal coordinated. Only the derivatives bearing an organic ligand configuration (*R*) exhibit KSP inhibitory activity. In contrast, all compounds bearing an organic ligand in the (S) configuration demonstrate no inhibitory activity toward the KSP at a concentration of 100, 300, and 1000 nM (Figure 8). The reference compound, ispinesib, shows a high KSP inhibitory activity (KSP residual activity 2.2%) at 100 nM concentration, while amine (*R*)-**1** decreases the KSP activity to about 35%. While the complexation of ruthenium leads to the nonactive complex **3a**, the other metal complexes **3b**–**d** are able to inhibit KSP activity with the most active rhodium **3c** (47.5%), followed by iridium **3d** (64.0%) and osmium **3b** (71.8%) complexes. Interestingly, the most cytotoxic iridium complexes **3d** and **4d** are practically deprived of KSP inhibitory activity. These results suggest the existence of another mechanism of anticancer activity than the ability to inhibit KSP activity.

### ROS Generation

**Figure 9:**
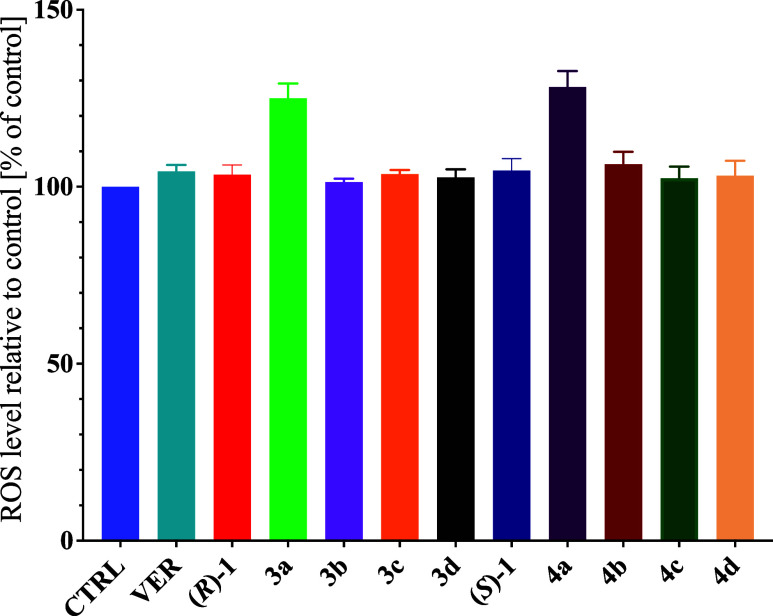
ROS generation in SW620 cells exposure to the studied compounds (1 μM). Ctrl expressed as 100%, cells in DMEM contained 0.1% DMSO as the control; verapamil (VER): cells in DMEM contained 0.1% DMSO and 10 μM VER as an ABC inhibitor to exclude the potential activity of ABC proteins. Results are presented as mean ± SEM, *n* = 3. No statistically significant differences were observed compared to the VER sample, (*R*)-**1** or (*S*)-**1** (*P* < 0.05, one-way ANOVA followed by the *posthoc* Tukey test).

Metal complexes often induce reactive oxygen species (ROS) generation in cells,^51^ which may increase their cytotoxic activity compared to purely organic molecules. To study the impact of the synthesized compounds on ROS production, we have measured the ROS generation in SW620 cells by the dihydrorhodamine 123 (DHR123) oxidation assay (Figure 9). However, there is no correlation between the antiproliferative potential and the ability of a compound to generate ROS. Only Ru derivatives (**3a** and **4a**) increase the level of ROS compared to the control or (*R*)- and (*S*)-**1**, and the level of the ROS generated by those complexes is virtually the same. In contrast, the other derivatives do not induce ROS generation.

## Conclusions

We designed and synthesized a series of organometallic half-sandwich Ru, Os, Rh, and Ir complexes bearing the pyridine-2-ylmethanimine bidentate ligand derived from 7-chloroquinazolin-4(3H)-one. We obtained compounds that exhibited nanomolar IC_50_ values, strongly dependent on the metal center, ligand configuration, and cell type. All studied molecules, with the most potent rhodium and iridium complexes derived from (*R*)-amine, force the cell cycle arrest in the G_2_/M phase. Only rhodium and iridium complexes derived from (*R*)-imine possess KSP inhibitory activity, however, to a lower extent than the corresponding amine. In contrast, all other complexes were significantly less or even nonactive. The complexation of imines derived from **1** only for Ru led to compounds able to do ROS generation. However, there is no clear correlation between the cytotoxicity, KSP inhibitory activity, impact on the cell cycle, and ROS generation ability. The results suggest that the complexation of the imines derived from amines (*R*)- and especially (*S*)-**1** led to compounds showing different mechanisms of activity than the organic ligands. Further studies are planned to determine the mechanism of biological activity of the synthesized compounds.

## Experimental Section

### Materials and Methods

All of the reactions were carried out under an argon atmosphere. All commercially available chemicals and solvents were of analytical grade and used without further purification. OsO_4_, RhCl_3_·xH_2_O, and IrCl_3_·xH_2_O were purchased from Precious Metals Online and Sigma-Aldrich. Bis[dichlorido(η^6^-*p*-cymene)ruthenium(II)] was purchased from Sigma-Aldrich. Bis[dichlorido(η^6^-*p*-cymene)osmium(II)],^52^ bis[dichlorido(η^5^-pentamethylcyclopentadienyl)rhodium(III)], and bis[dichlorido(η^5^-pentamethylcyclopentadienyl)iridium(III)]^53^ were synthesized as described previously. (*R*)-**1** and (*S*)-**1** were synthesized according to a reported procedure.^54^^1^H and ^13^C{^1^H} and ^1^H–^13^C HSQC NMR spectra were recorded at 294 K on a Bruker Avance III 600 MHz spectrometer at 600.3 MHz for ^1^H and at 150.1 MHz for ^13^C{^1^H}. The ^1^H and ^13^C{^1^H} chemical shifts were calibrated based on the residual ^1^H and ^13^C{^1^H} solvent peaks, i.e., δ = 3.58 ppm for ^1^H and 67.2 ppm for ^13^C in THF-*d*_8_, δ = 2.50 ppm for ^1^H and 39.5 ppm for ^13^C in dmso-*d6* and δ = 5.32 ppm for ^1^H and 53.8 ppm for ^13^C in CD_2_Cl_2_. The UV–vis spectra were recorded at 294 K on a PerkinElmer Lambda 45 spectrometer. Elemental analyses were performed at the Faculty of Chemistry, University of Lodz, Poland. The HPLC-MS analysis was performed using a Shimadzu Nexera XR system equipped with an SPD-M40 and an LCMS-2020 detector on a Phenomenex XB-C18 column (50 × 4.6 mm, 2.1 mm, 1.7 μm) using a mixture of 55% water with 0.01% HCOOH (eluent A), 22.5% methanol with 0.01% HCOOH (eluent B), and 22.5% acetonitrile with 0.01% HCOOH (eluent C) with a flow rate of 0.4 mL·min^–1^.

### General Procedure

To a solution of (*R*)-**1** or (*S*)-**1** (1 equiv) in anhydrous ethanol (12 mL), pyridine-2-carbaldehyde (2 equiv) was added and the resulting solution was refluxed under argon conditions for 1 h. Next, the [LMCl_2_]_2_ dimer (M = Rh/Ir, L = Cp* (Cp* = η^5^-1,2,3,4,5-pentamethylcyclopentadienyl) or M = Ru/Os, L = cym (cym = η^6^-*p*-cymene)) (0.49 equiv) was added, the mixture was cooled down to RT, and stirring was continued for an additional 3 h. The solvent was evaporated to c.a. 2 mL and methanol (3 mL) and water (10 mL) were added, followed by a saturated solution of KPF_6_ (5 mL). The precipitant was filtrated off, washed with water (3 × 10 mL), and dried. The products were purified by crystallization from the methanol/diethyl ether mixture.

#### **3a** [(cym)Ru((*R*)-**2**)Cl]PF_6_

Compound **3a** was synthesized in 69% yield (217 mg) according to the general procedure starting from 130 mg (0.38 mmol) of (*R*)-**1**, 79 mg (70 μL, 0.74 mmol) of pyridine-2-carbaldehyde, and 114 mg (0.19 mmol) of [(cym)RuCl_2_]_2_. Elemental analysis calculated for C_35_H_37_Cl_2_F_6_N_4_OPRu (846.64 g/mol) C 49.65, H 4.41, N 6.62; found C 49.37, H 4.61, N 6.85. HPLC-MS τ_1_ = 3.50 min calculated for C_35_H_37_Cl_2_N_4_ORu^+^ [M-PF_6_]^+^*m*/*z* = 701.1; found *m*/*z* = 701.4, τ_2_ = 5.22 min calculated for C_35_H_37_Cl_2_N_4_ORu^+^ [M-PF_6_]^+^*m*/*z* = 701.1; found *m*/*z* = 701.0. ^1^H NMR (600 MHz, CD_2_Cl_2_) δ 9.29 (d, *J* = 5.4 Hz, 1H, CH^Ar^), 9.27 (d, *J* = 5.5 Hz, 0.2H, CH^Ar^), 9.21 (s, 0.2H, CH^Ar^), 8.45 (s, 1H, CH^imine^), 8.33 (d, *J* = 8.5 Hz, 1H, CH^Ar^), 8.24 (d, *J* = 7.0 Hz, 1H, CH^Ar^), 8.20–8.18 (m, 1H, CH^Ar^), 8.15–8.11 (m, 0.4H, CH^Ar^), 7.92 (d, *J* = 1.9 Hz, 1H, CH^Ar^), 7.80–7.78 (m, 1H, CH^Ar^), 7.75–7.72 (m, 0.2H, CH^Ar^), 7.63 (d, *J* = 1.9 Hz, 0.2H, CH^Ar^), 7.61 (dd, *J* = 8.5, 2.0 Hz, 1H, CH^Ar^), 7.48–7.44 (m, 0.6H, CH^Ar^), 7.38 (t, *J* = 7.4 Hz, 2H, CH^Ar^), 7.32 (t, *J* = 7.1 Hz, 2H, CH^Ar^), 7.09 (d, *J* = 7.5 Hz, 2H, CH^Ar^), 6.09 (d, *J* = 16.7 Hz, 0.2H, CH_2_Ph), 5.96 (d, *J* = 6.7 Hz, 0.2H, 4-CH_3_C_6_H_4_CH(CH_3_)_2_), 5.92 (br s, 1H, 4-CH_3_C_6_H_4_CH(CH_3_)_2_), 5.87 (d, *J* = 6.0 Hz, 0.2H, 4-CH_3_C_6_H_4_CH(CH_3_)_2_), 5.80 (d, *J* = 17.3 Hz, 1H, CH_2_Ph), 5.71 (br s, 3H, 4-CH_3_C_6_H_4_CH(CH_3_)_2_), 5.66 (d, *J* = 6.2 Hz, 0.4H, 4-CH_3_C_6_H_4_CH(CH_3_)_2_), 5.48 (d, *J* = 5.9 Hz, 0.2H, NCH–CH(CH_3_)_2_), 5.41 (d, *J* = 10.3 Hz, 0.2H, 4-CH_3_C_6_H_4_CH(CH_3_)_2_), 5.23 (d, *J* = 16.6 Hz, 0.2H, CH_2_Ph_)_, 4.58 (d, *J* = 10.0 Hz, 1H, H-1′), 4.35 (d, *J* = 17.3 Hz, 1H, CH_2_Ph), 3.23–3.17 (m, 1H, H-2′), 2.98–2.93 (m, 0.2H, CH(CH_3_)_2_), 2.60–2.56 (m, 0.2H, CH(CH_3_)_2_), 2.45–2.40 (m, 1H, 4-CH_3_C_6_H_4_CH(CH_3_)_2_), 2.38 (s, 0.6H, 4-CH_3_C_6_H_4_CH(CH_3_)_2_), 1.84 (s, 3H, 4-CH_3_C_6_H_4_CH(CH_3_)_2_), 1.21 (d, *J* = 6.7 Hz, 0.6H, 4-CH_3_C_6_H_4_CH(CH_3_)_2_), 1.07 (d, *J* = 6.9 Hz, 0.6H, 4-CH_3_C_6_H_4_CH(CH_3_)_2_), 1.03–0.99 (m, 7H, 4-CH_3_C_6_H_4_CH(CH_3_)_2_ superimposed with H-3′), 0.89 (d, *J* = 6.3 Hz, 3H, H-3′), 0.86 (d, *J* = 7.0 Hz, 3H, 4-CH_3_C_6_H_4_CH(CH_3_)_2_), 0.55 (d, *J* = 6.5 Hz, 0.6H, 4-CH_3_C_6_H_4_CH(CH_3_)_2_). ^13^C{^1^H} NMR (151 MHz, CD_2_Cl_2_) δ 170.7 (CH^imine^), 161.5 (C^IV^), 156.0 (CH^Ar^), 153.3 (C^IV^), 153.0 (C^IV^), 147.4 (C^IV^), 141.4 (C^IV^), 140.3 (CH^Ar^), 136.2 (C^IV^), 131.2 (CH^Ar^), 130.3 (CH^Ar^), 129.9 (CH^Ar^), 129.8 (CH^Ar^), 129.2 (CH^Ar^), 128.6 (CH^Ar^), 127.1 (CH^Ar^), 127.0 (CH^Ar^), 126.3 (CH^Ar^), 120.2 (C^IV^), 46.6 (CH_2_), 32.0 (4-CH_3_C_6_H_4_CH(CH_3_)_2_), 31.2 (C-2′), 22.9 (4-CH_3_C_6_H_4_CH(CH_3_)_2_), 21.9 (4-CH_3_C_6_H_4_CH(CH_3_)_2_), 20.4 (C-3′), 19.8 (C-3′), 19.2 (4-CH_3_C_6_H_4_CH(CH_3_)_2_).

#### **3b** [(cym)Os((*R*)-**2**)Cl]PF_6_

Compound **3b** was synthesized in 37% yield (128 mg) according to the general procedure starting from 130 mg (0.38 mmol) of (*R*)-**1**, 79 mg (70 μL, 0.74 mmol) of pyridine-2-carbaldehyde, and 148 mg (0.19 mmol) of [(cym)OsCl_2_]_2_. Elemental analysis calculated for C_35_H_37_Cl_2_F_6_N_4_OOsP (935.80 g/mol) C 44.92, H 3.99, N 5.99; found C 44.68, H 3.96, N 6.13. HPLC-MS τ_1_ = 4.23 min calculated for C_35_H_37_Cl_2_N_4_OOs^+^ [M-PF_6_]^+^*m*/*z* = 791.2; found *m*/*z* = 791.3, τ_2_ = 7.06 min calculated for C_35_H_37_Cl_2_N_4_OOs^+^ [M-PF_6_]^+^*m*/*z* = 791.2; found *m*/*z* = 791.2. ^1^H NMR (600 MHz, CD_2_Cl_2_) δ 9.62 (s, 0.2H, CH^imine^), 9.20 (d, *J* = 5.5 Hz, 1H, CH^Ar^), 8.92 (s, 1H, CH^imine^), 8.40 (d, *J* = 7.4 Hz, 1H, CH^Ar^), 8.32 (d, *J* = 8.5 Hz, 1H, CH^Ar^), 8.28 (d, *J* = 7.8 Hz, 0.2H, CH^Ar^), 8.19 (d, *J* = 8.6 Hz, 0.3H, CH^Ar^), 8.16–8.14 (m, 1H, CH^Ar^), 8.10–8.09 (m, 0.3H, CH^Ar^), 7.90 (d, *J* = 1.9 Hz, 1H, CH^Ar^), 7.74–7.71 (m, 1H, CH^Ar^), 7.68–7.66 (m, 0.6H, CH^Ar^), 7.61 (dd, *J* = 8.4, 2.0 Hz, 1H, CH^Ar^), 7.47–7.45 (m, 1H, CH^Ar^), 7.40 (t, *J* = 7.5 Hz, 2H, CH^Ar^), 7.34 (t, *J* = 7.4 Hz, 1H, CH^Ar^), 7.30–7.27 (m, 1H, CH^Ar^), 7.13 (d, *J* = 7.5 Hz, 2H, CH^Ar^), 6.24 (d, *J* = 5.9 Hz, 0.3H, 4-CH_3_C_6_H_4_CH(CH_3_)_2_), 6.20 (d, *J* = 5.6 Hz, 1H, 4-CH_3_C_6_H_4_CH(CH_3_)_2_), 6.06 (d, *J* = 16.6 Hz, 0.4H, CH_2_Ph), 5.96 (br s, 1H, 4-CH_3_C_6_H_4_CH(CH_3_)_2_), 5.93–5.85 (m, 2H, 4-CH_3_C_6_H_4_CH(CH_3_)_2_), 5.81 (d, *J* = 17.0 Hz, 1H, CH_2_Ph), 5.70 (d, *J* = 5.5 Hz, 0.3H, 4-CH_3_C_6_H_4_CH(CH_3_)_2_), 5.41 (d, *J* = 10.3 Hz, 0.2H, H-1′), 5.14 (d, *J* = 16.5 Hz, 0.3H, CH_2_Ph), 4.80 (d, *J* = 9.9 Hz, 1H, H-1′), 4.52 (d, *J* = 13.6 Hz, 1H, CH_2_Ph), 3.16–3.10 (m, 1H, H-2′), 2.99–2.95 (m, 0.25H, H-2′), 2.91 (s, 0.1H), 2.82 (s, 0.1H), 2.50–2.46 (m, 0.2H), 2.43 (s, 0.7H, 4-CH_3_C_6_H_4_CH(CH_3_)_2_), 2.34–2.27 (m, 1H, 4-CH_3_C_6_H_4_CH(CH_3_)_2_), 1.90 (s, 3H, 4-CH_3_C_6_H_4_CH(CH_3_)_2_), 1.18 (d, *J* = 6.6 Hz, 1H, H-3′), 1.08 (d, *J* = 6.9 Hz, 0.8H,), 1.00 (d, *J* = 6.9 Hz, 3H, 4-CH_3_C_6_H_4_CH(CH_3_)_2_), 0.97 (d, *J* = 6.8 Hz, 3H, H-3′), 0.89 (d, *J* = 6.1 Hz, 3H, H-3′), 0.78 (d, *J* = 6.9 Hz, 3H, 4-CH_3_C_6_H_4_CH(CH_3_)_2_), 0.53 (d, *J* = 6.5 Hz, 0.7H, H-3′). ^13^C{^1^H} NMR (151 MHz, CD_2_Cl_2_) δ 172.8 (CH^imine^), 161.5 (C^IV^), 155.5 (CH^Ar^), 154.7 (C^IV^), 152.8 (C^IV^), 147.3 (C^IV^), 141.5 (C^IV^), 140.3 (CH^Ar^), 136.1 (C^IV^), 131.1 (CH^Ar^), 131.1 (C^IV^), 129.9 (CH^Ar^), 129.8 (CH^Ar^), 129.3 (CH^Ar^), 128.7 (CH^Ar^), 127.3 (CH^Ar^), 127.1 (CH^Ar^), 126.4 (CH^Ar^), 120.2 (C^IV^), 83.6 (NCH–CH(CH_3_)_2_), 80.8 (4-CH_3_C_6_H_4_CH(CH_3_)_2_), 76.9 (4-CH_3_C_6_H_4_CH(CH_3_)_2_), 73.9 (4-CH_3_C_6_H_4_CH(CH_3_)_2_), 46.9 (CH_2_Ph), 32.2 (4-CH_3_C_6_H_4_CH(CH_3_)_2_), 31.4 (NCH-CH(CH_3_)_2_) 23.5 (4-CH_3_C_6_H_4_CH(CH_3_)_2_), 21.9 (4-CH_3_C_6_H_4_CH(CH_3_)_2_), 20.5 (NCH–CH(CH_3_)_2_), 19.8 (4-CH_3_C_6_H_4_CH(CH_3_)_2_), 19.1 (4-CH_3_C_6_H_4_CH(CH_3_)_2_).

#### **3c** [(Cp*)Rh((*R*)-**2**)Cl]PF_6_

Compound **3c** was synthesized in 69% yield (536 mg) according to the general procedure starting from 313 mg (0.91 mmol) of (*R*)-**1**, 195 mg (174 μL, 1.82 mmol) of pyridine-2-carbaldehyde, and 277 mg (0.45 mmol) of [Cp*RhCl_2_]_2_. Elemental analysis calculated for C_35_H_38_Cl_2_F_6_N_4_OPRh (849.49 g/mol) C 49.49, H 4.51, N 6.60; found C 49.49, H 4.50, N 6.60. HPLC-MS τ_1_ = 2.67 min calculated for C_35_H_38_Cl_2_N_4_ORh^+^ [M-PF_6_]^+^*m*/*z* = 703.1; found *m*/*z* = 703.5, τ_2_ = 4.29 min calculated for C_35_H_38_Cl_2_N_4_ORh^+^ [M-PF_6_]^+^*m*/*z* = 703.1; found *m*/*z* = 703.5. ^1^H NMR (600 MHz, THF-*d*_8_) δ 10.76 (s, 0.1H, CH), 9.27 (s, 1H, CH^imine^), 8.89 (d, *J* = 5.4 Hz, 1H, CH^Ar^), 8.86 (d, *J* = 5.5 Hz, 0.1H, CH^Ar^), 8.66 (s, 0.1H, CH^Ar^), 8.24 (d, *J* = 7.4 Hz, 1H, CH^Ar^), 8.19 (t, *J* = 7.8 Hz, 1H, CH^Ar^), 8.15 (d, *J* = 8.5 Hz, 1H, CH^Ar^), 7.87 (d, *J* = 1.8 Hz, 0.2H, CH^Ar^), 7.84 (t, *J* = 6.4 Hz, 1H, CH^Ar^), 7.73 (d, *J* = 1.7 Hz, 1H, CH^Ar^), 7.56 (dd, *J* = 8.6, 2.0 Hz, 0.1H, CH^Ar^), 7.47 (dd, *J* = 8.6, 2.0 Hz, 1H, CH^Ar^), 7.39 (t, *J* = 7.5 Hz, 2H, CH^Ar^), 7.32–7.28 (m, 3.5H, CH^Ar^), 7.23 (d, *J* = 7.2 Hz, 0.1H, CH^Ar^), 7.20 (d, *J* = 7.9 Hz, 0.3H, CH^Ar^), 5.91 (d, *J* = 16.9 Hz, 1H, CH_2_Ph), 5.80 (d, *J* = 17.4 Hz, 0.1H, CH_2_Ph), 5.33 (d, *J* = 17.1 Hz, 1H, CH_2_Ph), 5.13 (d, *J* = 8.7 Hz, 1H, H-1′), 3.08–3.02 (m, 1H, H-2′), 2.39 (s, 0.4H), 1.73 (s, 15H, Cp*-CH_3_), 1.63 (s, 2H, Cp*-CH_3_), 1.13 (d, *J* = 6.7 Hz, 3H, H-3′), 0.95 (d, *J* = 6.6 Hz, 3H, H-3′). ^13^C{^1^H} NMR (151 MHz, THF-*d*_8_) δ 170.4 (CH^imine^), 161.5 (C^IV^), 157.0 (C^IV^), 154.7 (C^IV^), 153.6 (CH^Ar^), 148.2 (C^IV^), 140.8 (CH^Ar^), 140.7 (C^IV^), 137.0 (C^IV^), 131.1 (CH^Ar^), 130.7 (CH^Ar^), 129.6 (CH^Ar^), 129.5 (CH^Ar^), 129.3 (CH^Ar^), 128.3 (CH^Ar^), 128.3 (CH^Ar^), 127.6 (CH^Ar^), 127.1 (CH^Ar^), 120.4 (C^IV^), 98.6 (d, *J*_C–Rh_ = 7.7 Hz, Cp*), 73.2 (C-1′), 48.2 (CH_2_Ph), 35.1 (C-2′), 19.9 (C-3′), 18.8 (C-3′), 9.1 (Cp*-CH_3_).

#### **3d** [(Cp*)Ir((*R*)-**2**)Cl]PF_6_

Compound **3d** was synthesized in 73% yield (593 mg) according to the general procedure starting from 296 mg (0.87 mmol) of (*R*)-**1**, 185 mg (165 μL, 1.73 mmol) of pyridine-2-carbaldehyde, and 338 mg (0.42 mmol) of [Cp*IrCl_2_]_2_. Elemental analysis calculated for C_35_H_38_Cl_2_F_6_IrN_4_OP (938.80 g/mol) C 44.78, H 4.08, N 5.97; found C 44.83, H 4.20, N 5.97. HPLC-MS τ_1_ = 3.65 min calculated for C_35_H_38_Cl_2_N_4_OIr^+^ [M-PF_6_]^+^*m*/*z* = 793.2; found *m*/*z* = 793.4, τ_2_ = 7.45 min calculated for C_35_H_38_Cl_2_N_4_OIr^+^ [M-PF_6_]^+^*m*/*z* = 793.2; found *m*/*z* = 793.6. ^1^H NMR (600 MHz, THF-*d*_8_) δ 9.83 (s, 1H, CH^imine^), 9.14 (s, 0.4H), 8.86 (d, *J* = 5.4 Hz, 1H, CH^Ar^), 8.84 (d, *J* = 5.5 Hz, 0.4H, CH^Ar^), 8.42 (d, *J* = 7.6 Hz, 1H, CH^Ar^), 8.22 (d, *J* = 8.5 Hz, 0.6H, CH^Ar^), 8.17 (t, *J* = 7.6 Hz, 1H, CH^Ar^), 8.14 (d, *J* = 8.5 Hz, 1H, CH^Ar^), 7.87 (d, *J* = 2.0 Hz, 0.4H, CH^Ar^), 7.84–7.80 (m, 1.5H, CH^Ar^), 7.79 (d, *J* = 2.0 Hz, 1H, CH^Ar^), 7.57 (dd, *J* = 8.6, 2.0 Hz, 0.5H, CH^Ar^), 7.47 (dd, *J* = 8.5, 2.0 Hz, 1H, CH^Ar^), 7.38 (t, *J* = 7.5 Hz, 2H, CH^Ar^), 7.32–7.26 (m, 4H, CH^Ar^), 7.26–7.22 (m, 1H, CH^Ar^), 7.19 (d, *J* = 7.5 Hz, 1H, CH^Ar^), 5.87 (d, *J* = 17.1 Hz, 1H, CH_2_Ph), 5.78 (d, *J* = 17.2 Hz, 0.4H), 5.24 (d, *J* = 17.1 Hz, 1H, CH_2_Ph), 5.15 (d, *J* = 9.3 Hz, 1H, H-1′), 3.20–3.13 (m, 1H, H-2′), 2.95–2.89 (m, 0.4H, H-2′), 1.69 (s, 15H, Cp*-CH_3_), 1.60 (s, 7H, Cp*-CH_3_), 1.16 (d, *J* = 6.7 Hz, 3H, H-3′), 0.92 (d, *J* = 6.6 Hz, 3H, H-3′). ^13^C{^1^H} NMR (151 MHz, THF-*d*_8_) δ 172.0 (CH^imine^), 161.3 (C^IV^), 157.0 (C^IV^), 156.2 (C^IV^), 153.1 (CH^Ar^), 148.2 (C^IV^), 141.0 (CH^Ar^), 140.8 (CH^Ar^), 136.7 (C^IV^), 131.3 (CH^Ar^), 131.2 (CH^Ar^), 129.6 (CH^Ar^), 129.4 (CH^Ar^), 129.3 (CH^Ar^), 128.4 (CH^Ar^), 128.3 (CH^Ar^), 128.2 (CH^Ar^), 127.7 (CH^Ar^), 127.0 (CH^Ar^), 120.3 (C^IV^), 91.3 (Cp*), 74.8 (C-1′), 48.1 (CH_2_Ph), 35.9 (C-2′), 19.9 (C-3′), 19.2 (C-3′), 8.80 (Cp*-CH_3_).

#### **4a** [(cym)Ru((*S*)-**2**)Cl]PF_6_

Compound **4a** was synthesized in 68% yield (215 mg) according to the general procedure starting from 130 mg (0.38 mmol) of (*S*)-**1**, 79 mg (70 μL, 0.74 mmol) of pyridine-2-carbaldehyde, and 114 mg (0.19 mmol) of [(cym)RuCl_2_]_2_. Elemental analysis calculated for C_35_H_37_Cl_2_F_6_N_4_OPRu (846.64 g/mol) C 49.65, H 4.41, N 6.62; found C 49.41, H 4.59, N 6.80. HPLC-MS τ_1_ = 3.39 min calculated for C_35_H_37_Cl_2_N_4_ORu^+^ [M-PF_6_]^+^*m*/*z* = 701.1; found *m*/*z* = 701.3, τ_2_ = 5.07 min calculated for C_35_H_37_Cl_2_N_4_ORu^+^ [M-PF_6_]^+^*m*/*z* = 701.1; found *m*/*z* = 700.8. ^1^H NMR (600 MHz, CD_2_Cl_2_) δ 9.29 (d, *J* = 5.4 Hz, 1H, CH^Ar^), 9.27 (d, *J* = 5.6 Hz, 0.2H, CH^Ar^), 9.21 (s, 0.2H, CH^imine^), 8.45 (s, 1H, CH^imine^), 8.33 (d, *J* = 8.5 Hz, 1H CH^Ar^), 8.24 (d, *J* = 7.1 Hz, 1H, CH^Ar^), 8.19 (t, *J* = 3.8 Hz, 1H, CH^Ar^), 8.15–8.11 (m, 0.4H, CH^Ar^), 7.92 (d, *J* = 1.9 Hz, 1H, CH^Ar^), 7.80–7.78 (m, 1H, CH^Ar^), 7.74–7.72 (m, 0.2H, CH^Ar^), 7.63 (d, *J* = 1.8 Hz, 0.2H, CH^Ar^), 7.61 (dd, *J* = 8.6, 2.0 Hz, 1H, CH^Ar^), 7.48–7.44 (m, 0.7H, CH^Ar^), 7.41 (d, *J* = 7.4 Hz, 0.2H, CH^Ar^), 7.38 (t, *J* = 7.4 Hz, 2H, CH^Ar^), 7.32 (t, *J* = 6.8 Hz, 2H, CH^Ar^), 7.09 (d, *J* = 7.5 Hz, 2H, CH^Ar^), 6.09 (d, *J* = 16.9 Hz, 0.2H, CH_2_Ph), 5.96 (d, *J* = 6.5 Hz, 0.3H, 4-CH_3_C_6_H_4_CH(CH_3_)_2_), 5.92 (br s, 1H, 4-CH_3_C_6_H_4_CH(CH_3_)_2_), 5.87 (d, *J* = 6.0 Hz, 0.3H, 4-CH_3_C_6_H_4_CH(CH_3_)_2_), 5.80 (d, *J* = 16.8 Hz, 1H, CH_2_Ph), 5.71 (br s, 3H, 4-CH_3_C_6_H_4_CH(CH_3_)_2_), 5.66 (d, *J* = 6.4 Hz, 0.3H, 4-CH_3_C_6_H_4_CH(CH_3_)_2_), 5.48 (d, *J* = 5.9 Hz, 0.2H, H-1′), 5.41 (d, *J* = 10.1 Hz, 0.2H, H-1′), 5.23 (d, *J* = 16.5 Hz, 0.2H, CH_2_Ph), 4.58 (d, *J* = 9.2 Hz, 1H, H-1′), 4.35 (d, *J* = 14.5 Hz, 1H, CH_2_Ph), 3.23–3.17 (m, 1H, H-2′), 2.98–2.94 (m, 0.2H, H-2′), 2.60–2.55 (m, 0.2H, 4-CH_3_C_6_H_4_CH(CH_3_)_2_), 2.45–2.40 (m, 1H, 4-CH_3_C_6_H_4_CH(CH_3_)_2_), 2.38 (s, 0.6H, 4-CH_3_C_6_H_4_CH(CH_3_)_2_), 1.84 (s, 3H, 4-CH_3_C_6_H_4_CH(CH_3_)_2_), 1.21 (d, *J* = 6.6 Hz, 0.8H, H-3′), 1.07 (d, *J* = 6.9 Hz, 0.7H, 4-CH_3_C_6_H_4_CH(CH_3_)_2_), 1.03–0.99 (m, 7H, H-3′, superimposed with 4-CH_3_C_6_H_4_CH(CH_3_)_2_), 0.89 (d, *J* = 6.3 Hz, 3H, H-3′), 0.86 (d, *J* = 7.0 Hz, 3H 4-CH_3_C_6_H_4_CH(CH_3_)_2_), 0.55 (d, *J* = 6.5 Hz, 0.7H, H-3′). ^13^C{^1^H} NMR (151 MHz, CD_2_Cl_2_) δ 170.7 (CH^imine^), 161.5 (C^IV^), 156.0 (CH^Ar^), 153.3 (C^IV^), 153.0 (C^IV^), 147.4 (C^IV^), 141.4 (C^IV^), 140.3 (CH^Ar^), 136.2 (C^IV^), 131.2 (CH^Ar^), 130.3 (CH^Ar^), 129.9 (CH^Ar^), 129.8 (CH^Ar^), 129.2 (CH^Ar^), 128.5 (CH^Ar^), 127.1 (CH^Ar^), 127.0 (CH^Ar^), 126.3 (CH^Ar^), 120.2 (C^IV^), 82.9 (C-1′), 79.5 (4-CH_3_C_6_H_4_CH(CH_3_)_2_), 47.9 (CH_2_Ph), 46.6 (CH_2_Ph), 32.0 (4-CH_3_C_6_H_4_CH(CH_3_)_2_), 31.3 (C-2′), 22.9 (C–H), 22.7 (C–H), 22.6 (C–H), 22.3 (C–H), 21.9 (4-CH_3_C_6_H_4_CH(CH_3_)_2_), 20.4 (C-3′), 19.8 (4-CH_3_C_6_H_4_CH(CH_3_)_2_), 19.2 (4-CH_3_C_6_H_4_CH(CH_3_)_2_).

#### **4b** [(cym)Os((*S*)-**2**)Cl]PF_6_

Compound **4b** was synthesized in 41% yield (144 mg) according to the general procedure starting from 130 mg (0.38 mmol) of (*S*)-**1**, 79 mg (70 μL, 0.74 mmol) of pyridine-2-carbaldehyde, and 149 mg (0.19 mmol) of [(cym)OsCl_2_]_2_. Elemental analysis calculated for C_35_H_37_Cl_2_F_6_N_4_OOsP (935.80 g/mol) C 44.92, H 3.99, N 5.99; found C 44.75, H 4.09, N 5.78. HPLC-MS τ_1_ = 4.24 min calculated for C_35_H_37_Cl_2_N_4_OOs^+^ [M-PF_6_]^+^*m*/*z* = 791.2; found *m*/*z* = 791.4, τ_2_ = 7.04 min calculated for C_35_H_37_Cl_2_N_4_OOs^+^ [M-PF_6_]^+^*m*/*z* = 791.2; found *m*/*z* = 791.6. ^1^H NMR (600 MHz, CD_2_Cl_2_) δ 9.62 (s, 0.25H, CH^Ar^), 9.20 (d, *J* = 5.5 Hz, 1H, CH^Ar^), 8.91 (s, 1H, CH^imine^), 8.40 (d, *J* = 7.5 Hz, 1H, CH^Ar^), 8.31 (d, *J* = 8.5 Hz, 1H, CH^Ar^), 8.27 (d, *J* = 7.8 Hz, 0.25H, CH^Ar^), 8.19 (d, *J* = 8.5 Hz, 0.25H, CH^Ar^), 8.17–8.14 (m, 1H, CH^Ar^), 8.11–8.08 (m, 0.25H, CH^Ar^), 7.90 (d, *J* = 1.9 Hz, 1H, CH^Ar^), 7.74–7.72 (m, 1H, CH^Ar^), 7.68–7.66 (m, 0.5H, CH^Ar^), 7.61 (dd, *J* = 8.2, 2.0 Hz, 1H, CH^Ar^), 7.47–7.45 (m, 0.75H, CH^Ar^), 7.39 (t, *J* = 7.5 Hz, 2H, CH^Ar^), 7.34 (t, *J* = 7.4 Hz, 1H, CH^Ar^), 7.29 (d, *J* = 7.5 Hz, 0.5H, CH^Ar^), 7.12 (d, *J* = 7.5 Hz, 2H, CH^Ar^), 6.24 (d, *J* = 6.1 Hz, 0.4H, 4-CH_3_C_6_H_4_CH(CH_3_)_2_), 6.19 (d, *J* = 5.5 Hz, 1H, 4-CH_3_C_6_H_4_CH(CH_3_)_2_), 6.06 (d, *J* = 16.5 Hz, 0.4H, CH_2_Ph), 5.96 (br s, 1H, 4-CH_3_C_6_H_4_CH(CH_3_)_2_), 5.92–5.84 (m, 2H, 4-CH_3_C_6_H_4_CH(CH_3_)_2_), 5.81 (d, *J* = 16.7 Hz, 1H, CH_2_Ph), 5.69 (d, *J* = 5.5 Hz, 0.25H, 4-CH_3_C_6_H_4_CH(CH_3_)_2_), 5.40 (d, *J* = 10.4 Hz, 0.25H, NCH–CH(CH_3_)_2_), 5.13 (d, *J* = 16.4 Hz, 0.25H, CH_2_Ph), 4.78 (d, *J* = 9.9 Hz, 1H, NCH–CH(CH_3_)_2_), 4.50 (d, *J* = 16.2 Hz, 1H, CH_2_Ph), 3.42 (s, 0.1H), 3.16–3.10 (m, 1H, NCH-CH(CH_3_)_2_), 3.00–2.95 (m, 0.25H, NCH-CH(CH_3_)_2_), 2.43 (s, 1H, 4-CH_3_C_6_H_4_CH(CH_3_)_2_), 2.32–2.27 (m, 1H, 4-CH_3_C_6_H_4_CH(CH_3_)_2_), 1.90 (s, 3H, 4-CH_3_C_6_H_4_CH(CH_3_)_2_), 1.35 (d, *J* = 6.9 Hz, 0.25H), 1.17 (d, *J* = 6.7 Hz, 1H, NCH–CH(CH_3_)_2_), 1.07 (d, *J* = 6.9 Hz, 1H), 1.00 (d, *J* = 6.9 Hz, 3H, 4-CH_3_C_6_H_4_CH(CH_3_)_2_), 0.96 (d, *J* = 6.8 Hz, 3H, NCH–CH(CH_3_)_2_), 0.89 (d, *J* = 6.1 Hz, 3H, NCH–CH(CH_3_)_2_), 0.77 (d, *J* = 6.9 Hz, 3H, 4-CH_3_C_6_H_4_CH(CH_3_)_2_), 0.51 (d, *J* = 6.6 Hz, 0.75H, NCH–CH(CH_3_)_2_). ^13^C{^1^H} NMR (151 MHz, CD_2_Cl_2_) δ 172.4 (CH^imine^), 161.1 (C^IV^), 155.2 (CH^Ar^), 154.2 (C^IV^), 152.3 (C^IV^), 146.9 (C^IV^), 141.1 (C^IV^), 140.0 (CH^Ar^), 135.7 (C^IV^), 130.8 (CH^Ar^), 129.5 (CH^Ar^), 129.4 (CH^Ar^), 128.9 (CH^Ar^), 128.2 (CH^Ar^), 126.7 (CH^Ar^), 126.0 (CH^Ar^), 119.7 (C^IV^), 88.3 (4-CH_3_C_6_H_4_CH(CH_3_)_2_), 83.2 (C-1′), 46.5 (CH_2_), 31.8 (4-CH_3_C_6_H_4_CH(CH_3_)_2_), 30.9 (C-2′), 23.1 (4-CH_3_C_6_H_4_CH(CH_3_)_2_), 21.4 (4-CH_3_C_6_H_4_CH(CH_3_)_2_), 20.1 (C-3′), 19.3 (C-3′), 18.7 (4-CH_3_C_6_H_4_CH(CH_3_)_2_).

#### **4c** [(Cp*)Rh((*S*)-**2**)Cl]PF_6_

Compound **4c** was synthesized in 68% yield (535 mg) according to the general procedure starting from 313 mg (0.91 mmol) of (*S*)-**1**, 195 mg (174 μL, 1.82 mmol) of pyridine-2-carbaldehyde, and 276 mg (0.45 mmol) of [Cp*RhCl_2_]_2_. Elemental analysis calculated for C_35_H_38_Cl_2_F_6_N_4_OPRh (849.49 g/mol) C 49.49, H 4.51, N 6.60; found C 49.53, H 4.49, N 6.55. HPLC-MS τ_1_ = 2.79 min calculated for C_35_H_38_Cl_2_N_4_ORh^+^ [M-PF_6_]^+^*m*/*z* = 703.1; found *m*/*z* = 703.5, τ_2_ = 4.20 min calculated for C_35_H_38_Cl_2_N_4_ORh^+^ [M-PF_6_]^+^*m*/*z* = 703.1; found *m*/*z* = 703.5. ^1^H NMR (600 MHz, THF-*d*_8_) δ 10.77 (s, 0.1H), 9.27 (s, 1H, CH^imine^), 8.90 (d, *J* = 5.3 Hz, 1H, CH^Ar^), 8.86 (d, *J* = 5.1 Hz, 0.1H, CH^Ar^), 8.66 (s, 0.1H), 8.24 (d, *J* = 7.1 Hz, 1H, CH^Ar^), 8.19 (t, *J* = 7.7 Hz, 1H, CH^Ar^), 8.15 (d, *J* = 8.5 Hz, 1H, CH^Ar^), 7.87 (d, *J* = 1.7 Hz, 0.2H, CH^Ar^), 7.84 (t, *J* = 6.0 Hz, 1H, CH^Ar^), 7.73 (d, *J* = 1.8 Hz, 1H, CH^Ar^), 7.56 (dd, *J* = 8.5, 2.0 Hz, 0.1H, CH^Ar^), 7.47 (dd, *J* = 8.5, 2.0 Hz, 1H, CH^Ar^), 7.39 (t, *J* = 7.4 Hz, 2H, CH^Ar^), 7.32–7.28 (m, 3.5H, CH^Ar^), 7.23 (d, *J* = 7.4 Hz, 0.1H, CH^Ar^), 7.20 (d, *J* = 7.7 Hz, 0.3H, CH^Ar^) 5.91 (d, *J* = 16.7 Hz, 1H, CH_2_Ph), 5.80 (d, *J* = 17.2 Hz, 0.1H, CH_2_Ph), 5.33 (d, *J* = 17.1 Hz, 1H, CH_2_Ph), 5.13 (d, *J* = 8.7 Hz, 1H, H-1′), 3.08–3.02 (m, 1H, H-2′), 2.39 (s, 0.5H), 1.73 (s, 15H, Cp*-CH_3_), 1.63 (s, 2H, Cp*-CH_3_), 1.13–1.10 (m, 3H, H-3′ superimposed with the diethyl ether signal), 0.95 (d, *J* = 6.6 Hz, 3H, H-3′). ^13^C{^1^H} NMR (151 MHz, THF-*d*_8_) δ 170.4 (CH^imine^), 161.5 (C^IV^), 154.7 (C^IV^), 153.6 (CH^Ar^), 148.2 (C^IV^), 140.8 (CH^Ar^), 140.7 (C^IV^), 137.0 (C^IV^), 131.1 (CH^Ar^), 130.6 (CH^Ar^), 129.6 (CH^Ar^), 129.5 (CH^Ar^), 129.3 (CH^Ar^), 128.3 (CH^Ar^), 128.3 (CH^Ar^), 127.6 (CH^Ar^), 127.1 (CH^Ar^), 120.4 (C^IV^), 98.6 (d, J_Rh–C_ = 7.8 Hz, Cp*), 73.2 (C-1′), 48.2 (CH_2_Ph), 35.1 (C-2′) 19.9 (C-3′), 18.8 (C-3′), 9.1 (Cp*-CH_3_).

#### **4d** [(Cp*)Ir((*S*)-**2**)Cl]PF_6_

Compound **4d** was synthesized in 72% yield (413 mg) according to the general procedure starting from 208 mg (0.61 mmol) of (*S*)-**1**, 131 mg (115 μL, 1.22 mmol) of pyridine-2-carbaldehyde, and 237 mg (0.30 mmol) of [Cp*IrCl_2_]_2_. Elemental analysis calculated for C_35_H_38_Cl_2_F_6_IrN_4_OP (938.80 g/mol) C 44.78, H 4.08, N 5.97; found C 44.69, H 4.05, N 5.86. HPLC-MS τ_1_ = 3.81 min calculated for C_35_H_38_Cl_2_N_4_OIr^+^ [M-PF_6_]^+^*m*/*z* = 793.2; found *m*/*z* = 793.6, τ_2_ = 7.53 min calculated for C_35_H_38_Cl_2_N_4_OIr^+^ [M-PF_6_]^+^*m*/*z* = 793.2; found *m*/*z* = 793.5. ^1^H NMR (600 MHz, THF-*d*_8_) δ 9.82 (s, 0.7H, CH^imine^) 9.14 (s, 1H, CH^imine^), 8.87 (d, *J* = 5.4 Hz, 0.7H, CH^Ar^), 8.84 (d, *J* = 5.4 Hz, 1H, CH^Ar^), 8.41 (d, *J* = 7.7 Hz, 0.7H, CH^Ar^), 8.22 (d, *J* = 8.5 Hz, 1.3H, CH^Ar^), 8.18–8.16 (m, 1.4H, CH^Ar^), 8.14 (d, *J* = 8.5 Hz, 1H, CH^Ar^), 7.87 (d, *J* = 1.9 Hz, 1H, CH^Ar^), 7.83–7.80 (m, 1.7H, CH^Ar^), 7.79 (d, *J* = 1.9 Hz, 0.7H, CH^Ar^), 7.57 (dd, *J* = 8.6, 1.9 Hz, 1H, CH^Ar^), 7.47 (dd, *J* = 8.5, 2.0 Hz, 0.7H, CH^Ar^), 7.38 (t, *J* = 7.5 Hz, 1.5H, CH^Ar^), 7.33–7.28 (m, 4H, CH^Ar^), 7.24 (t, *J* = 7.3 Hz, 1H, CH^Ar^), 7.19 (d, *J* = 7.5 Hz, 2H, CH^Ar^), 5.87 (d, *J* = 17.0 Hz, 0.7H, CH_2_Ph), 5.78 (d, *J* = 17.1 Hz, 1H, CH_2_Ph), 5.24 (d, *J* = 17.1 Hz, 0.8H, CH_2_Ph), 5.15 (d, *J* = 9.3 Hz, 1H, H-1′), 4.74 (br s, 0.7H, CH_2_Ph), 3.19–3.13 (m, 0.7H, H-2′), 2.95–2.87 (m, 1H, H-2′), 1.70 (s, 11.5H, Cp*-CH_3_), 1.61 (s, 15H, Cp*-CH_3_), 1.19 (br s, 3H, H-3′) superimposed with 1.16 (d, *J* = 6.7 Hz, 3H, H-3′), 0.93 (d, *J* = 6.6 Hz, 2.3H, H-3′). ^13^C{^1^H} NMR (151 MHz, THF-*d*_8_) δ 172.0 (CH^imine^), 161.4 (C^IV^), 161.3 (C^IV^), 156.9 (C^IV^), 156.2 (C^IV^), 153.1 (CH^Ar^), 152.7 (CH^Ar^), 148.2 (C^IV^), 147.9 (C^IV^), 141.1 (CH^Ar^), 140.8 (CH^Ar^), 140.6 (C^IV^), 136.7 (C^IV^), 131.3 (CH^Ar^), 131.2 (CH^Ar^), 129.6 (CH^Ar^), 129.4 (CH^Ar^), 129.3 (CH^Ar^), 128.7 (CH^Ar^), 128.4 (CH^Ar^), 128.3 (CH^Ar^), 128.2 (CH^Ar^), 127.7 (CH^Ar^), 127.0 (CH^Ar^), 126.7 (CH^Ar^), 121.1 (C^IV^), 120.4 (C^IV^), 91.4 (Cp*), 91.3 (Cp*), 74.8 (C-1′), 48.1 (CH_2_Ph), 35.9 (C-2′), 20.2 (C-3′), 19.9 (C-3′), 19.2 (C-3′), 19.1 (C-3′), 8.8 (Cp*-CH_3_), 8.8 (Cp*-CH_3_).

### Stability Studies

The stability of **3a**–**d** was studied in the presence of l-cysteine or l-histidine. **3a**–**d** were dissolved in DMSO and added to 0.2 mM aqueous solution of l-cysteine or l-histidine to achieve the complex concentration of 20 μM while keeping the DMSO concentration at 0.5 vol %. UV–vis spectra were recorded over 2 h with 10 min intervals. HPLC-MS analysis with them using UV–vis spectroscopy and HPLC-MS analysis were performed on a Phenomenex XB-C18 column (50 × 4.6 mm, 2.1 mm, 1.7 μm) using a mixture of 55% water with 0.01% HCOOH (eluent A), 22.5% methanol with 0.01% HCOOH (eluent B), and 22.5% acetonitrile with 0.01% HCOOH (eluent C) with a flow rate of 0.4 mL·min^–1^.

### Cell Lines

Cell lines used in this study were purchased from the American Type Culture Collection via LGC Standards. Human normal lung fibroblasts (MRC-5), alveolar basal epithelial cell adenocarcinoma (A549), colorectal adenocarcinoma (Colo205), hepatocellular carcinoma (HepG2), breast adenocarcinoma (MCF7), and colorectal adenocarcinoma (SW620) and its MDR variants^55^ were cultured in standard conditions (37 °C, 5% CO_2_, 100% relative humidity) in high glucose DMEM medium supplemented with GlutaMax, HEPES (ThermoFisher Scientific) and 10% fetal bovine serum (EURx, Poland). All cell lines were tested for *Mycoplasma* contamination using a MycoProbe mycoplasma detection kit (R&D System).

### Assaying the Antiproliferative Potential

For this purpose, neutral red uptake assay was performed. 10^4^ of cells were seeded per well of a 96-well plate and left overnight to allow cells to attach to the surface. Then, the cells were exposed to a desired concentration of tested compounds. Stock solutions were prepared in DMSO and were used immediately after preparation. The final concentration of DMSO was constant and nontoxic (0.1% v/v). After 70 h of culture, neutral red was added to the final concentration of 1 mM. After 2 h of incubation with the dye, the medium was aspirated and cells were washed with ice-cold PBS. The dye was released using 100 μL of the solubilizer (1% acetic acid in 50% ethanol) on an orbital shaker (10 min). The absorbance at 540 nm was measured using an EnVision multilabel plate reader (PerkinElmer). The results were presented as a percentage of control. The IC_90_ and IC_50_ parameters were calculated using GraphPad Prism v9 software using the five-parameter nonlinear logistic regression model.

### Cell Cycle

SW620 and SW620E cells lines (vulnerable and resistant variants, respectively) were seeded in 6-well plates at a density of 10^5^ cells per well. After the time necessary for the cells to attach to the surface, the cells were treated with tested compounds at a concentration equal to IC_90_ for parent compounds (15 nM for (*R*) series and 23 nM for (*S*) series). After 24 h, the cells were trypsinized and fixed with ice-cold 70% v/v ethanol. The cells were stained with 75 μM propidium iodide with 50 Kunitz units of RNase A in PBS for 30 min at 37 °C. All samples were analyzed using a LSRII flow cytometer (Becton Dickinson) at a PE channel (526/26 nm). Cell cycle phase distribution was determined using a built-in cell cycle module (Watson pragmatic algorithm) by FlowJo 7.6.1 software.

### Reactive Oxygen Species Assay

Dihydrorhodamine 123 oxidation was used as an indicator of intracellular ROS production. For this purpose, SW620 cells were seeded in 6-well plates at a density of 10^5^ cells per well. The cells were left overnight (time needed for them to attach to the surface). Then, 1 μM tested compounds were added along with 1 μM DHR123. Additionally, since DHR123 is a substrate of ABCB1 (which may interfere in this assay), 10 μM verapamil, an inhibitor of this protein, was added. The cells were cultured for an additional 4 h at 37 °C, and then the cells were harvested by trypsinization, resuspended in a complete medium, and analyzed using a LSRII flow cytometer (Becton Dickinson) in a FITC channel (530/30 nm). The results are presented as a percentage of control (median fluorescence in the presence of DMSO).

### Kinesin ATPase Inhibition Assay

The potential kinesin modulatory activity of tested compounds was performed using a Kinesin ATPase end-point biochem kit (Cytoskeleton, Inc.). Compounds were dissolved in DMSO (the final concentration did not exceed 0.1%). The experiment was performed according to the manufacturer’s instructions. One μg of tested kinesin (KSP) was used per reaction. Phosphate release was measured at the absorbance 650 nm using an EnVision multilabel plate reader (PerkinElmer).

## Data Availability

The crystal structure of **4a** was deposited with CCDC and assigned deposition number 2207900. It can be accessed free of charge via www.ccdc.cam.ac.uk/data_request/cif, by emailing at data_request@ccdc.cam.ac.uk, or by contacting the Cambridge Crystallographic Data Centre, 12 Union Road, Cambridge CB2 1EZ, U.K.; fax: + 44 1223 336033.

## Supporting Information Available

Additional figures and tables illustrating the HPLC-MS analysis of final products, UV–vis spectra, copies of ^1^H and ^13^C{^1^H} NMR spectra, ^1^H–^13^C HSQC NMR spectra and ^1^H DOSY spectra, the cell cycle phase distribution table for SW620 cells, and the graphical representation of IC_50_ values (PDF)

## References

[ref1] Giaquinto A. N.; Sung H.; Miller K. D.; Kramer J. L.; Newman L. A.; Minihan A.; Jemal A.; Siegel R. L. Breast Cancer Statistics, 2022. Ca-Cancer J. Clin. 2022, 72, 524–541. 10.3322/caac.21754. 36190501

[ref2] De Silva F.; Alcorn J. A Tale of Two Cancers: A Current Concise Overview of Breast and Prostate Cancer. Cancers 2022, 14 (12), 2954 10.3390/cancers14122954. 35740617 PMC9220807

[ref3] Miller K. D.; Nogueira L.; Devasia T.; Mariotto A. B.; Yabroff K. R.; Jemal A.; Kramer J.; Siegel R. L. Cancer treatment and survivorship statistics, 2022. Ca-Cancer J. Clin. 2022, 72 (5), 409–436. 10.3322/caac.21731. 35736631

[ref4] Zugazagoitia J.; Guedes C.; Ponce S.; Ferrer I.; Molina-Pinelo S.; Paz-Ares L. Current Challenges in Cancer Treatment. Clin. Ther. 2016, 38 (7), 1551–1566. 10.1016/j.clinthera.2016.03.026. 27158009

[ref5] Vale R. D. The Molecular Motor Toolbox for Intracellular Transport. Cell 2003, 112 (4), 467–480. 10.1016/S0092-8674(03)00111-9. 12600311

[ref6] Howard J.; Hyman A. A. Microtubule polymerases and depolymerases. Curr. Opin. Cell Biol. 2007, 19 (1), 31–35. 10.1016/j.ceb.2006.12.009. 17184986

[ref7] Cella D.; Peterman A.; Hudgens S.; Webster K.; Socinski M. A. Measuring the side effects of taxane therapy in oncology: the functional assesment of cancer therapy-taxane (FACT-taxane). Cancer 2003, 98 (4), 822–831. 10.1002/cncr.11578. 12910528

[ref8] Shahin R.; Aljamal S. Kinesin spindle protein inhibitors in cancer: from high throughput screening to novel therapeutic strategies. Future Sci. OA 2022, 8 (3), FSO778 10.2144/fsoa-2021-0116. 35251692 PMC8890118

[ref9] Chen Y.; Hancock W. O. Kinesin-5 is a microtubule polymerase. Nat. Commun. 2015, 6 (1), 8160 10.1038/ncomms9160. 26437877 PMC4600729

[ref10] Mann B. J.; Wadsworth P. Kinesin-5 Regulation and Function in Mitosis. Trends Cell Biol. 2019, 29 (1), 66–79. 10.1016/j.tcb.2018.08.004. 30220581

[ref11] Maliga Z.; Kapoor T. M.; Mitchison T. J. Evidence that Monastrol Is an Allosteric Inhibitor of the Mitotic Kinesin Eg5. Chem. Biol. 2002, 9 (9), 989–996. 10.1016/S1074-5521(02)00212-0. 12323373

[ref12] Bongero D.; Paoluzzi L.; Marchi E.; Zullo K. M.; Neisa R.; Mao Y.; Escandon R.; Wood K.; O′Connor O. A. The novel kinesin spindle protein (KSP) inhibitor SB-743921 exhibits marked activity in in vivo and in vitro models of aggressive large B-cell lymphoma. Leuk. Lymphoma 2015, 56 (10), 2945–2952. 10.3109/10428194.2015.1020058. 25860245

[ref13] Novais P.; Silva P. M. A.; Amorim I.; Bousbaa H. Second-Generation Antimitotics in Cancer Clinical Trials. Pharmaceutics 2021, 13 (7), 1011 10.3390/pharmaceutics13071011. 34371703 PMC8309102

[ref14] Cox C. D.; Coleman P. J.; Breslin M. J.; Whitman D. B.; Garbaccio R. M.; Fraley M. E.; Buser C. A.; Walsh E. S.; Hamilton K.; Schaber M. D.; et al. Kinesin Spindle Protein (KSP) Inhibitors. 9. Discovery of (2S)-4-(2,5-Difluorophenyl)-N-[(3R,4S)-3-fluoro-1-methylpiperidin-4-yl]-2-(hydroxymethyl)-N-methyl-2-phenyl-2,5-dihydro-1H-pyrrole-1-carboxamide (MK-0731) for the Treatment of Taxane-Refractory Cancer. J. Med. Chem. 2008, 51 (14), 4239–4252. 10.1021/jm800386y. 18578472

[ref15] Kim K. H.; Xie Y.; Tytler E. M.; Woessner R.; Mor G.; Alvero A. B. KSP inhibitor ARRY-520 as a substitute for Paclitaxel in Type I ovarian cancer cells. J. Transl. Med. 2009, 7 (1), 63 10.1186/1479-5876-7-63. 19619321 PMC2719595

[ref16] Woessner R.; Tunquist B.; Lemieux C.; Chlipala E.; Jackinsky S.; Dewolf W. Jr.; Voegtli W.; Cox A.; Rana S.; Lee P.; Walker D. ARRY-520, a novel KSP inhibitor with potent activity in hematological and taxane-resistant tumor models. Anticancer Res. 2009, 29 (11), 4373–4380. 10.1186/1479-5876-7-63. 20032381

[ref17] Purcell J. W.; Davis J.; Reddy M.; Martin S.; Samayoa K.; Vo H.; Thomsen K.; Bean P.; Kuo W. L.; Ziyad S.; et al. Activity of the Kinesin Spindle Protein Inhibitor Ispinesib (SB-715992) in Models of Breast Cancer. Clin. Cancer Res. 2010, 16 (2), 566–576. 10.1158/1078-0432.CCR-09-1498. 20068098 PMC2844774

[ref18] Khoury H. J.; Garcia-Manero G.; Borthakur G.; Kadia T.; Foudray M. C.; Arellano M.; Langston A.; Bethelmie-Bryan B.; Rush S.; Litwiler K.; et al. A phase 1 dose-escalation study of ARRY-520, a kinesin spindle protein inhibitor, in patients with advanced myeloid leukemias. Cancer 2012, 118 (14), 3556–3564. 10.1002/cncr.26664. 22139909 PMC4984525

[ref19] Carter B. Z.; Mak D. H.; Woessner R.; Gross S.; Schober W. D.; Estrov Z.; Kantarjian H.; Andreeff M. Inhibition of KSP by ARRY-520 induces cell cycle block and cell death via the mitochondrial pathway in AML cells. Leukemia 2009, 23 (10), 1755–1762. 10.1038/leu.2009.101. 19458629 PMC3593228

[ref20] Institute N. C. SB-715992 in Treating Patients with Recurrent or Metastatic Head and Neck Cancer, 2005. https://ClinicalTrials.gov/show/NCT00095628.

[ref21] McQuitty R. J. Metal-based drugs. Sci. Prog. 2014, 97 (Pt 1), 1–19. 10.3184/003685014x13898980185076. 24800466 PMC10365534

[ref22] Boros E.; Dyson P. J.; Gasser G. Classification of Metal-Based Drugs according to Their Mechanisms of Action. Chem. 2020, 6 (1), 41–60. 10.1016/j.chempr.2019.10.013. 32864503 PMC7451962

[ref23] Allardyce C. S.; Dyson P. J. Metal-based drugs that break the rules. Dalton Trans. 2016, 45 (8), 3201–3209. 10.1039/C5DT03919C. 26820398

[ref24] Jaouen G.; Vessières A.; Top S. Ferrocifen type anti cancer drugs. Chem. Soc. Rev. 2015, 44 (24), 8802–8817. 10.1039/C5CS00486A. 26486993

[ref25] Simović A. R.; Masnikosa R.; Bratsos I.; Alessio E. Chemistry and reactivity of ruthenium(II) complexes: DNA/protein binding mode and anticancer activity are related to the complex structure. Coord. Chem. Rev. 2019, 398, 113011 10.1016/j.ccr.2019.07.008.

[ref26] Meier-Menches S. M.; Gerner C.; Berger W.; Hartinger C. G.; Keppler B. K. Structure–activity relationships for ruthenium and osmium anticancer agents – towards clinical development. Chem. Soc. Rev. 2018, 47 (3), 909–928. 10.1039/C7CS00332C. 29170783

[ref27] Steel T. R.; Walsh F.; Wieczorek-Błauż A.; Hanif M.; Hartinger C. G. Monodentately-coordinated bioactive moieties in multimodal half-sandwich organoruthenium anticancer agents. Coord. Chem. Rev. 2021, 439, 213890 10.1016/j.ccr.2021.213890.

[ref28] Tremlett W. D. J.; Goodman D. M.; Steel T. R.; Kumar S.; Wieczorek-Błauż A.; Walsh F. P.; Sullivan M. P.; Hanif M.; Hartinger C. G. Design concepts of half-sandwich organoruthenium anticancer agents based on bidentate bioactive ligands. Coord. Chem. Rev. 2021, 445, 213950 10.1016/j.ccr.2021.213950.

[ref29] Hanif M.; Babak M. V.; Hartinger C. G. Development of anticancer agents: wizardry with osmium. Drug Discovery Today 2014, 19 (10), 1640–1648. 10.1016/j.drudis.2014.06.016. 24955838

[ref30] Geldmacher Y.; Oleszak M.; Sheldrick W. S. Rhodium(III) and iridium(III) complexes as anticancer agents. Inorg. Chim. Acta 2012, 393, 84–102. 10.1016/j.ica.2012.06.046.

[ref31] Pettinari R.; Marchetti F.; Condello F.; Pettinari C.; Lupidi G.; Scopelliti R.; Mukhopadhyay S.; Riedel T.; Dyson P. J. Ruthenium(II)–Arene RAPTA Type Complexes Containing Curcumin and Bisdemethoxycurcumin Display Potent and Selective Anticancer Activity. Organometallics 2014, 33 (14), 3709–3715. 10.1021/om500317b.

[ref32] Pettinari R.; Marchetti F.; Pettinari C.; Condello F.; Petrini A.; Scopelliti R.; Riedel T.; Dyson P. J. Organometallic rhodium(iii) and iridium(iii) cyclopentadienyl complexes with curcumin and bisdemethoxycurcumin co-ligands. Dalton Trans. 2015, 44 (47), 20523–20531. 10.1039/C5DT03037D. 26548708

[ref33] Plażuk D.; Wieczorek A.; Ciszewski W. M.; Kowalczyk K.; Błauż A.; Pawlędzio S.; Makal A.; Eurtivong C.; Arabshahi H. J.; Reynisson J.; et al. Synthesis and in vitro Biological Evaluation of Ferrocenyl Side-Chain-Functionalized Paclitaxel Derivatives. ChemMedChem 2017, 12 (22), 1882–1892. 10.1002/cmdc.201700576. 28941201

[ref34] Plażuk D.; Wieczorek A.; Błauż A.; Rychlik B. Synthesis and biological activities of ferrocenyl derivatives of paclitaxel. MedChemComm 2012, 3 (4), 498–501. 10.1039/c2md00315e.

[ref35] Nicolaus N.; Zapke J.; Riesterer P.; Neudörfl J.-M.; Prokop A.; Oschkinat H.; Schmalz H.-G. Azides Derived from Colchicine and their Use in Library Synthesis: a Practical Entry to New Bioactive Derivatives of an Old Natural Drug. ChemMedChem 2010, 5 (5), 661–665. 10.1002/cmdc.201000063. 20229567

[ref36] Wardle N. J.; Kalber T.; Bell J. D.; Bligh S. W. A. Synthesis and characterisation of a novel tubulin-directed DO3A–colchicine conjugate with potential theranostic features. Bioorg. Med. Chem. Lett. 2011, 21 (11), 3346–3348. 10.1016/j.bmcl.2011.04.014. 21524911

[ref37] Kowalczyk K.; Błauż A.; Ciszewski W. M.; Wieczorek A.; Rychlik B.; Plażuk D. Colchicine metallocenyl bioconjugates showing high antiproliferative activities against cancer cell lines. Dalton Trans. 2017, 46 (48), 17041–17052. 10.1039/C7DT03229C. 29185574

[ref38] Ang W. H.; De Luca A.; Chapuis-Bernasconi C.; Juillerat-Jeanneret L.; Lo Bello M.; Dyson P. J. Organometallic ruthenium inhibitors of glutathione-S-transferase P1–1 as anticancer drugs. ChemMedChem 2007, 2 (12), 1799–1806. 10.1002/cmdc.200700209. 17918761

[ref39] Ang W. H.; Parker L. J.; De Luca A.; Juillerat-Jeanneret L.; Morton C. J.; Lo Bello M.; Parker M. W.; Dyson P. J. Rational design of an organometallic glutathione transferase inhibitor. Angew. Chem., Int. Ed. 2009, 48 (21), 3854–3857. 10.1002/anie.200900185. 19396894

[ref40] Schmid W. F.; John R. O.; Arion V. B.; Jakupec M. A.; Keppler B. K. Highly Antiproliferative Ruthenium(II) and Osmium(II) Arene Complexes with Paullone-Derived Ligands. Organometallics 2007, 26 (26), 6643–6652. 10.1021/om700813c.

[ref41] Beaupérin M.; Polat D.; Roudesly F.; Top S.; Vessières A.; Oble J.; Jaouen G.; Poli G. Approach to ferrocenyl-podophyllotoxin analogs and their evaluation as anti-tumor agents. J. Organomet. Chem. 2017, 839, 83–90. 10.1016/j.jorganchem.2017.02.005.

[ref42] Wieczorek A.; Błauż A.; Makal A.; Rychlik B.; Plażuk D. Synthesis and evaluation of biological properties of ferrocenyl-podophyllotoxin conjugates. Dalton Trans. 2017, 46 (33), 10847–10858. 10.1039/C7DT02107K. 28752867

[ref43] Kowalczyk K.; Błauż A.; Moscoh Ayine-Tora D.; Hartinger C. G.; Rychlik B.; Plażuk D. Design, Synthesis, and Evaluation of Biological Activity of Ferrocene-Ispinesib Hybrids: Impact of a Ferrocenyl Group on the Antiproliferative and Kinesin Spindle Protein Inhibitory Activity. Chem. - Eur. J. 2023, 29 (49), e202300813 10.1002/chem.202300813. 37332065

[ref44] Łomzik M.; Hanif M.; Budniok A.; Błauż A.; Makal A.; Tchoń D. M.; Leśniewska B.; Tong K. K. H.; Movassaghi S.; Söhnel T.; et al. Metal-Dependent Cytotoxic and Kinesin Spindle Protein Inhibitory Activity of Ru, Os, Rh, and Ir Half-Sandwich Complexes of Ispinesib-Derived Ligands. Inorg. Chem. 2020, 59 (20), 14879–14890. 10.1021/acs.inorgchem.0c00957. 33003697 PMC7584371

[ref45] Łomzik M.; Błauż A.; Głodek M.; Makal A.; Tchoń D.; Ayine-Tora D. M.; Hartinger C.; Rychlik B.; Plażuk D. Organometallic Ru, Os, Rh and Ir half-sandwich conjugates of ispinesib – impact of the organometallic group on the antimitotic activity. Dalton Trans. 2023, 52 (34), 11859–11874. 10.1039/D3DT01217D. 37464882

[ref46] Chow M. J.; Licona C.; Pastorin G.; Mellitzer G.; Ang W. H.; Gaiddon C. Structural tuning of organoruthenium compounds allows oxidative switch to control ER stress pathways and bypass multidrug resistance. Chem. Sci. 2016, 7 (7), 4117–4124. 10.1039/C6SC00268D. 30155055 PMC6013925

[ref47] Gichumbi J. M.; Friedrich H. B.; Omondi B. Synthesis and characterization of piano-stool ruthenium complexes with N,N′-pyridine imine bidentate ligands and their application in styrene oxidation. J. Organomet. Chem. 2016, 808, 87–96. 10.1016/j.jorganchem.2016.02.015.

[ref48] Gichumbi J. M.; Friedrich H. B.; Omondi B. Synthesis and characterization of half-sandwich ruthenium(II) complexes with N-alkyl pyridyl-imine ligands and their application in transfer hydrogenation of ketones. Transit. Met. Chem. 2016, 41 (8), 867–877. 10.1007/s11243-016-0089-5.

[ref49] Burris H. A. 3rd; Jones S. F.; Williams D. D.; Kathman S. J.; Hodge J. P.; Pandite L.; Ho P. T.; Boerner S. A.; Lorusso P. A phase I study of ispinesib, a kinesin spindle protein inhibitor, administered weekly for three consecutive weeks of a 28-day cycle in patients with solid tumors. Invest. New Drugs 2011, 29 (3), 467–472. 10.1007/s10637-009-9374-x. 20069338

[ref50] El Zouhairi M.; Charabaty A.; Pishvaian M. J. Molecularly targeted therapy for metastatic colon cancer: proven treatments and promising new agents. Gastrointest Cancer Res. 2011, 4 (1), 15–21. 21464866 PMC3070284

[ref51] Kandioller W.; Balsano E.; Meier S. M.; Jungwirth U.; Göschl S.; Roller A.; Jakupec M. A.; Berger W.; Keppler B. K.; Hartinger C. G. Organometallic anticancer complexes of lapachol: metal centre-dependent formation of reactive oxygen species and correlation with cytotoxicity. Chem. Commun. 2013, 49 (32), 3348–3350. 10.1039/c3cc40432c. 23505633

[ref52] Kiel W. A.; Ball R. G.; Graham W. A. G. Carbonyl-η-hexamethylbenzene complexes of osmium. Carbon-hydrogen activation by (η-C6Me6)Os(CO)(H)2. J. Organomet. Chem. 1990, 383 (1–3), 481–496. 10.1016/0022-328X(90)85149-S.

[ref53] White C.; Yates A.; Maitlis P. M.; Heinekey D. M. (η5-Pentamethylcyclopentadienyl)Rhodium and -Iridium Compounds. Inorg. Synth. 1992, 29, 228–234. 10.1002/9780470132609.ch53.

[ref54] Holland J. P.; Jones M. W.; Cohrs S.; Schibli R.; Fischer E. Fluorinated quinazolinones as potential radiotracers for imaging kinesin spindle protein expression. Bioorg. Med. Chem. 2013, 21 (2), 496–507. 10.1016/j.bmc.2012.11.013. 23245569

[ref55] Błauż A.; Rychlik B. Drug-selected cell line panels for evaluation of the pharmacokinetic consequences of multidrug resistance proteins. J. Pharmacol. Toxicol. Methods 2017, 84, 57–65. 10.1016/j.vascn.2016.11.001. 27838457

