# Supplementary material for: Development of Half-Sandwich Ru, Os, Rh, and Ir Complexes Bearing the Pyridine-2-ylmethanimine Bidentate Ligand Derived from 7-Chloroquinazolin-4(3H)-one with Enhanced Antiproliferative Activity
Source: ACS Omega. 2024 Apr 13;9(16):18224–37. doi: 10.1021/acsomega.3c10482 (PMC11044151; doi:10.1021/acsomega.3c10482)
Supplement: Supplementary file 1 — ao3c10482_si_001.pdf [file ao3c10482_si_001.pdf]

Development of half-sandwich Ru, Os, Rh and Ir complexes bearing pyridine-2-ylmethanimine bidentate ligand derived from 7-chloroquinazolin-4(3H)-one with enhanced antiproliferative activity

Michał Łomzik[1,], Andrzej Błauż[2], Daniel Tchoń[3,4], Anna Makal[3], Błażej Rychlik[2], Damian Plażuk[1,]**

[1] University of Lodz, Faculty of Chemistry, Department of Organic Chemistry, ul. Tamka 12, 91-403 Łódź, Poland

[2] University of Lodz, Faculty of Biology and Environmental Protection, Department of Oncobiology and Epigenetics, Cytometry Lab, ul. Pomorska 141/143, 90-236 Łódź, Poland

[3] Laboratory for Structural and Biochemical Research (LBSBio), Biological and Chemical Research Centre, Department of Chemistry, University of Warsaw, ul. Zwirki i Wigury 101, 02-089 Warszawa, Poland

[4] Molecular Biophysics and Integrated Bioimaging Division, Lawrence Berkeley National Laboratory, Berkeley, CA 94720, USA

Diffusion-ordered spectroscopy (DOSY) experiments 2

VT-NMR spectra 4

HPLC-MS analysis 9

X-ray diffraction experimental details 17

Stability studies - UV-Vis spectra 23

NMR Spectra 34

Bibliography 57

Diffusion-ordered spectroscopy (DOSY) experiments

The DOSY ^{1}H NMR spectra were recorded in DMSO-d_6 at 300 K (VT-regulated) with 24036 time domain points and 64 scans per spectrum using the dstebpgp3s pulse sequence with convection compensation[1, 2] as delivered with the Bruker Topspin software. 64 dummy scans were performed to equilibrate the sample and avoid artifacts in the first 1D spectrum. The gradient was incremented linearly in 64 steps from 2% to 95%. P30 and D20 were set to 1800 μs and 1200 ms respectively.

The 2D DOSY maps were generated by the built-in Topspin dosy2d routine using exponential fitting and displayed in linear F1 scale.

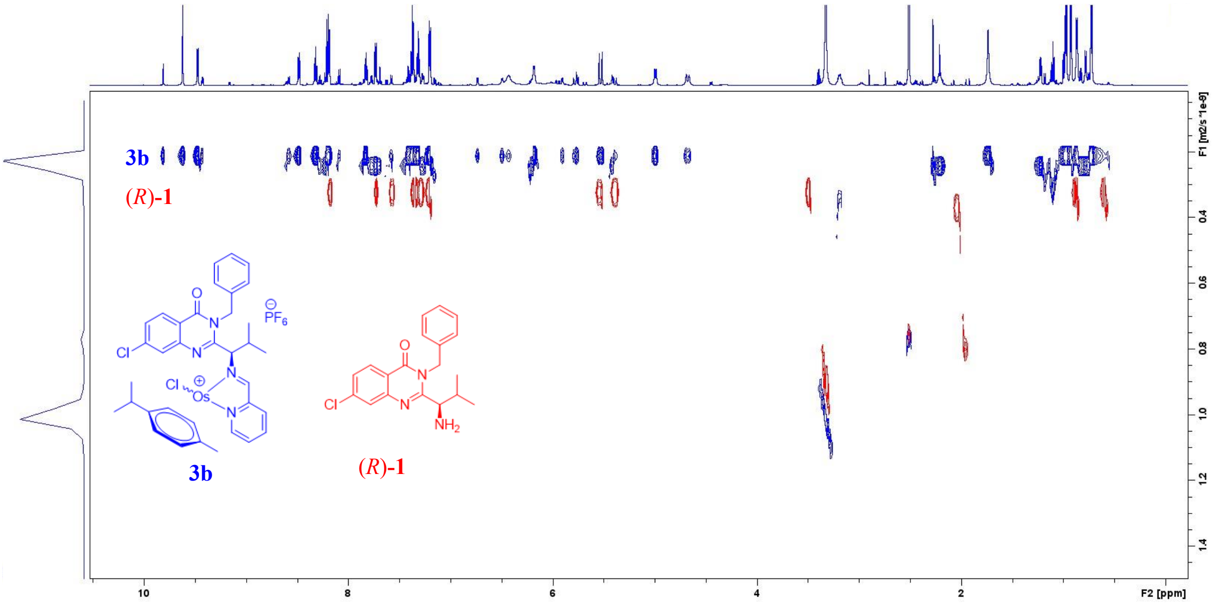

Figure S1. ^{1}H DOSY spectra of **3b (blue)** with overlapped spectra of (*R*)-**1 (red)** in DMSO-d_6

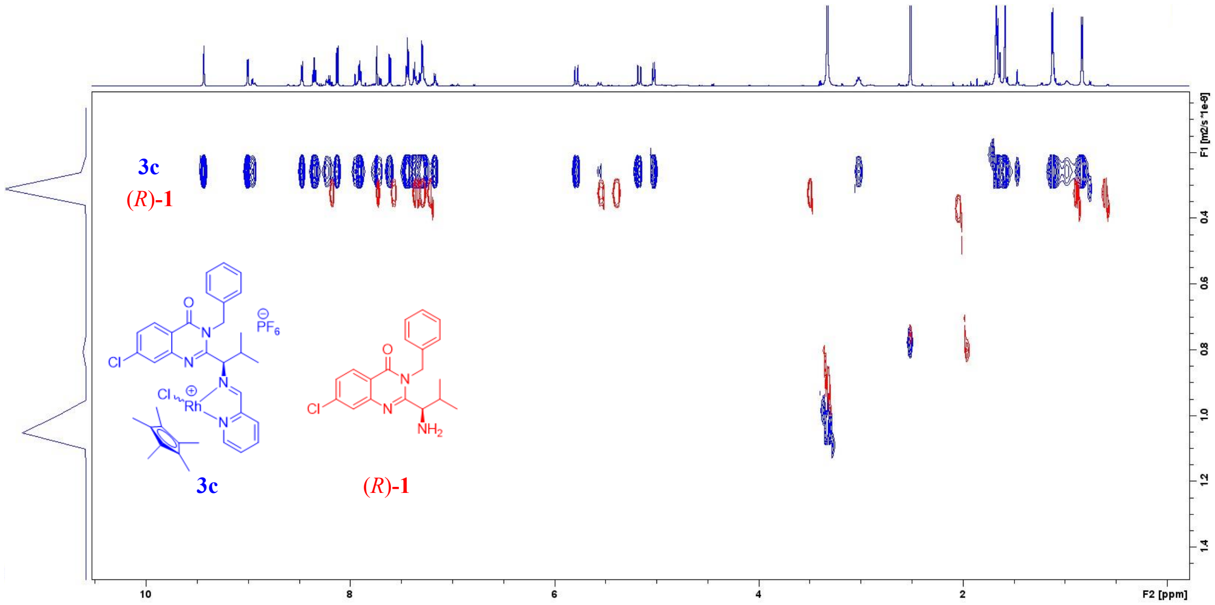

Figure S2. ^{1}H DOSY spectra of **3c (blue)** with overlapped spectra of (*R*)-**1 (red)** in DMSO-d_6

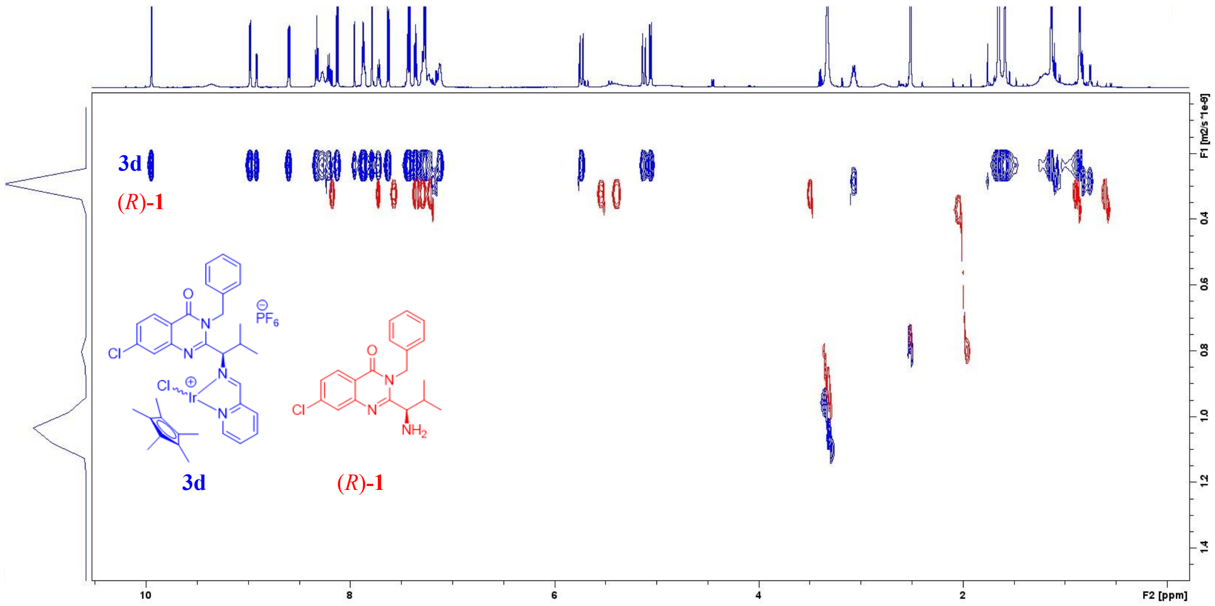

Figure S3. ^{1}H DOSY spectra of **3d (blue)** with overlapped spectra of (*R*)-**1 (red)** in DMSO-d_6

VT-NMR spectra

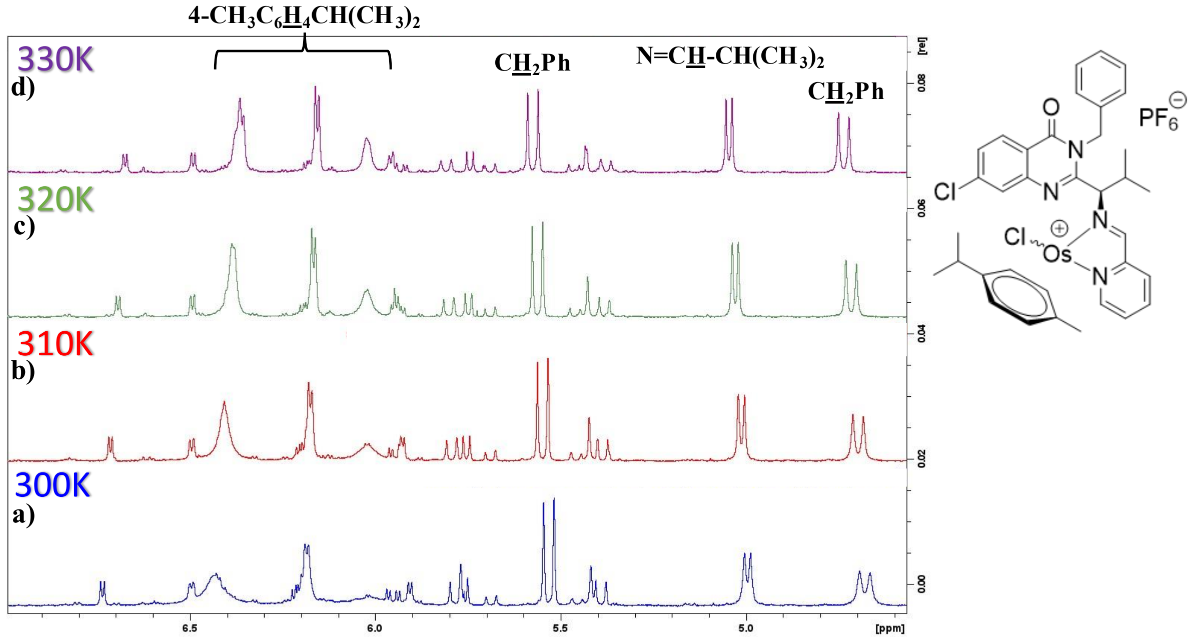

Figure S4. VT-NMR experiments for **3b**. ^{1}H NMR spectra in DMSO-d_6 (range 7.0 ppm to 4.6 ppm) at (a) 300K, (b) 310K, (c) 320K, (d) 330K; *denoted signals assigned to solvated compound.

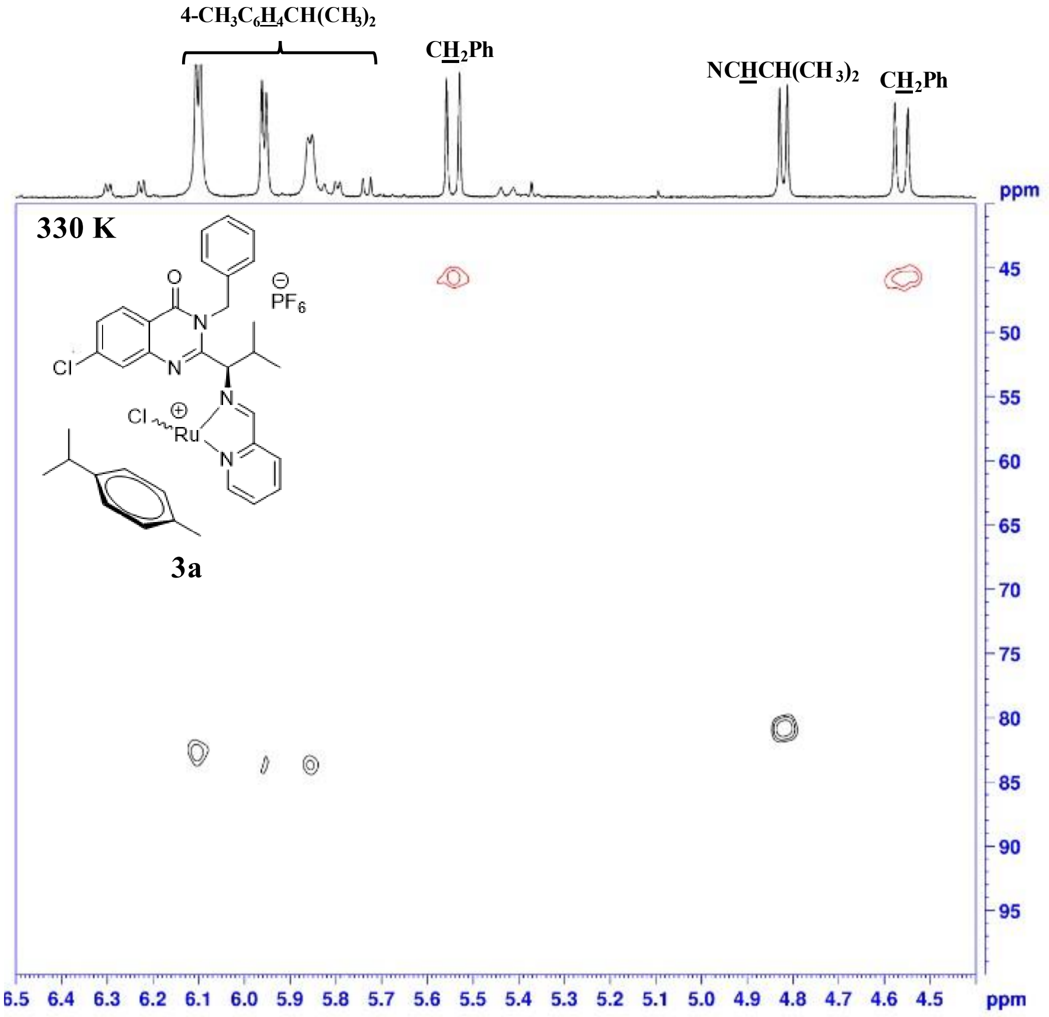

Figure S5. ^{1}H-^{13}C HSQC NMR spectrum of **3a** in DMSO-d_6 recorded at 330K.

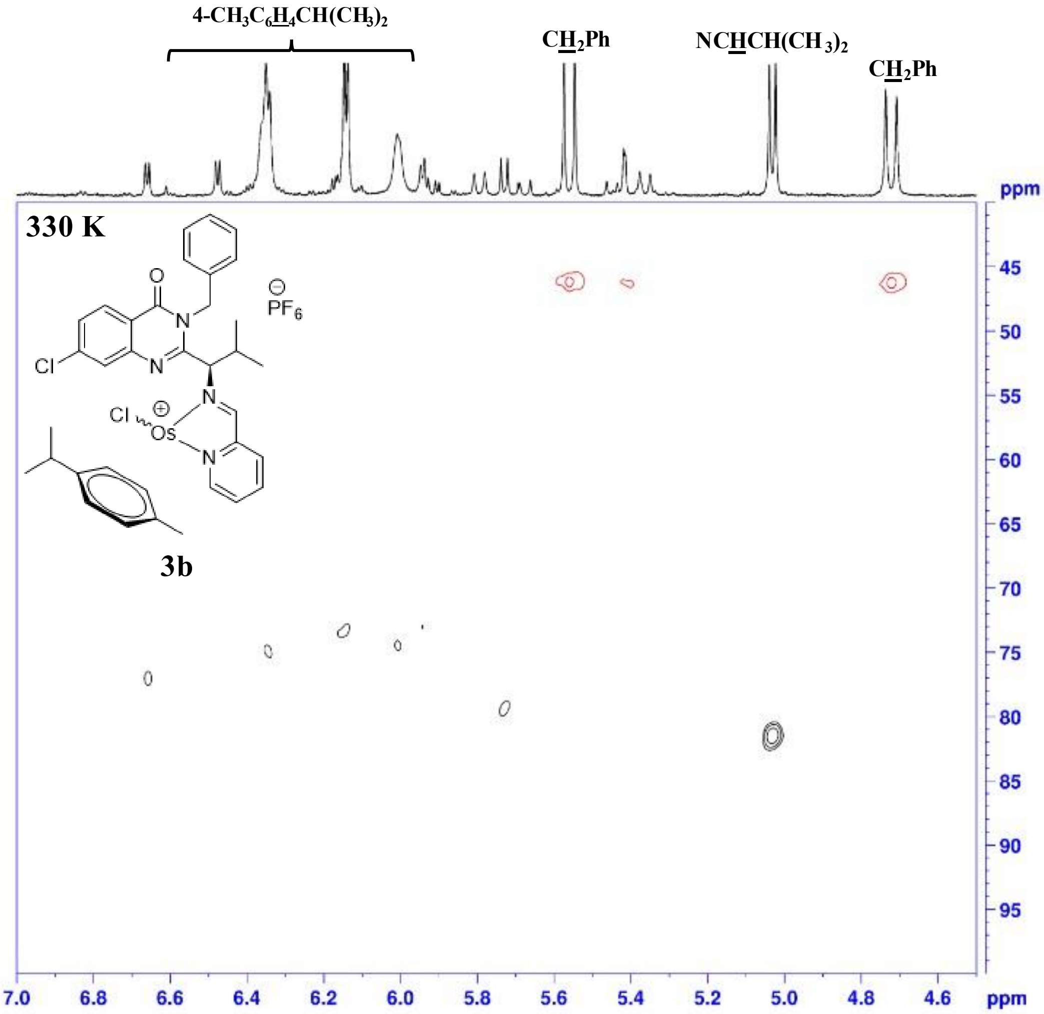

Figure S6. ^{1}H-^{13}C HSQC NMR spectrum of **3b** in DMSO-d_6 recorded at 330K.

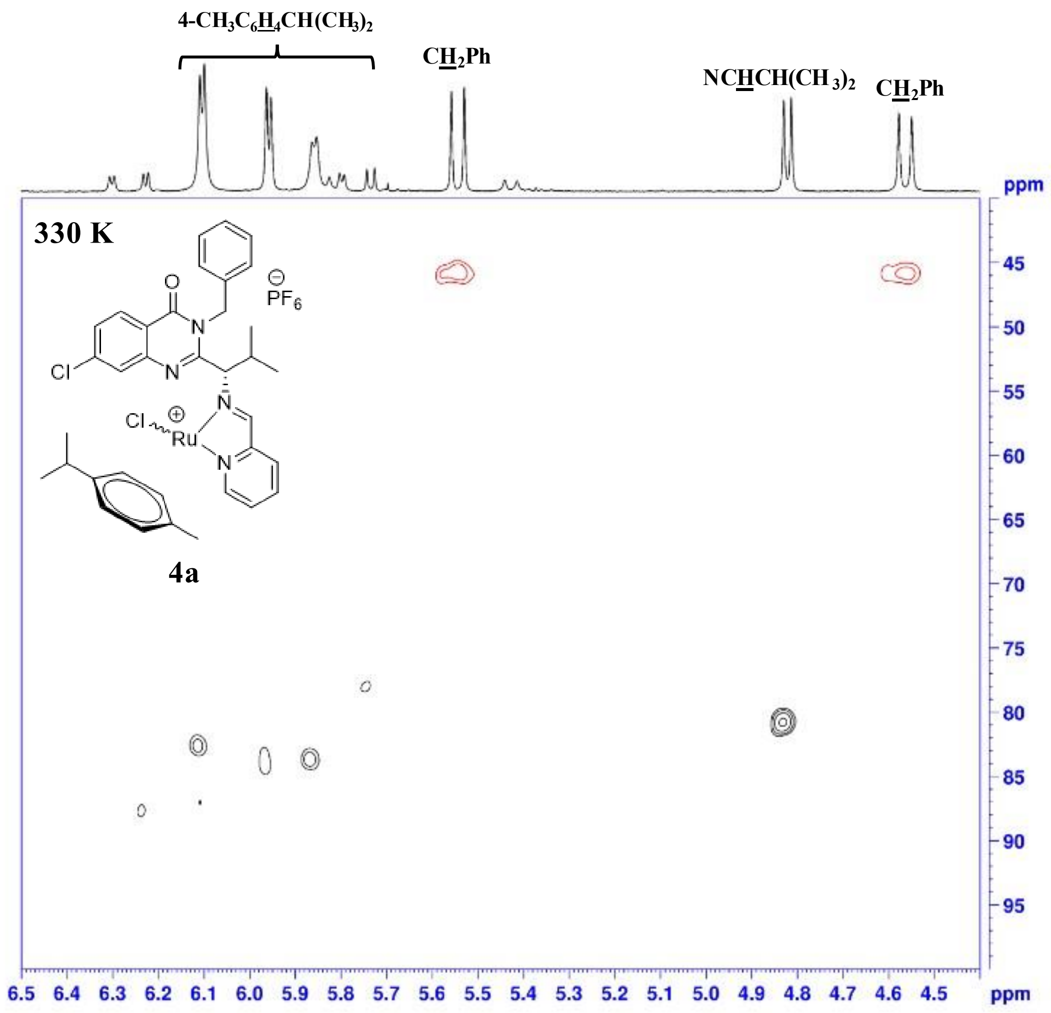

Figure S7. ^{1}H-^{13}C HSQC NMR of **4a** in DMSO-d$_6$ recorded at 330K.

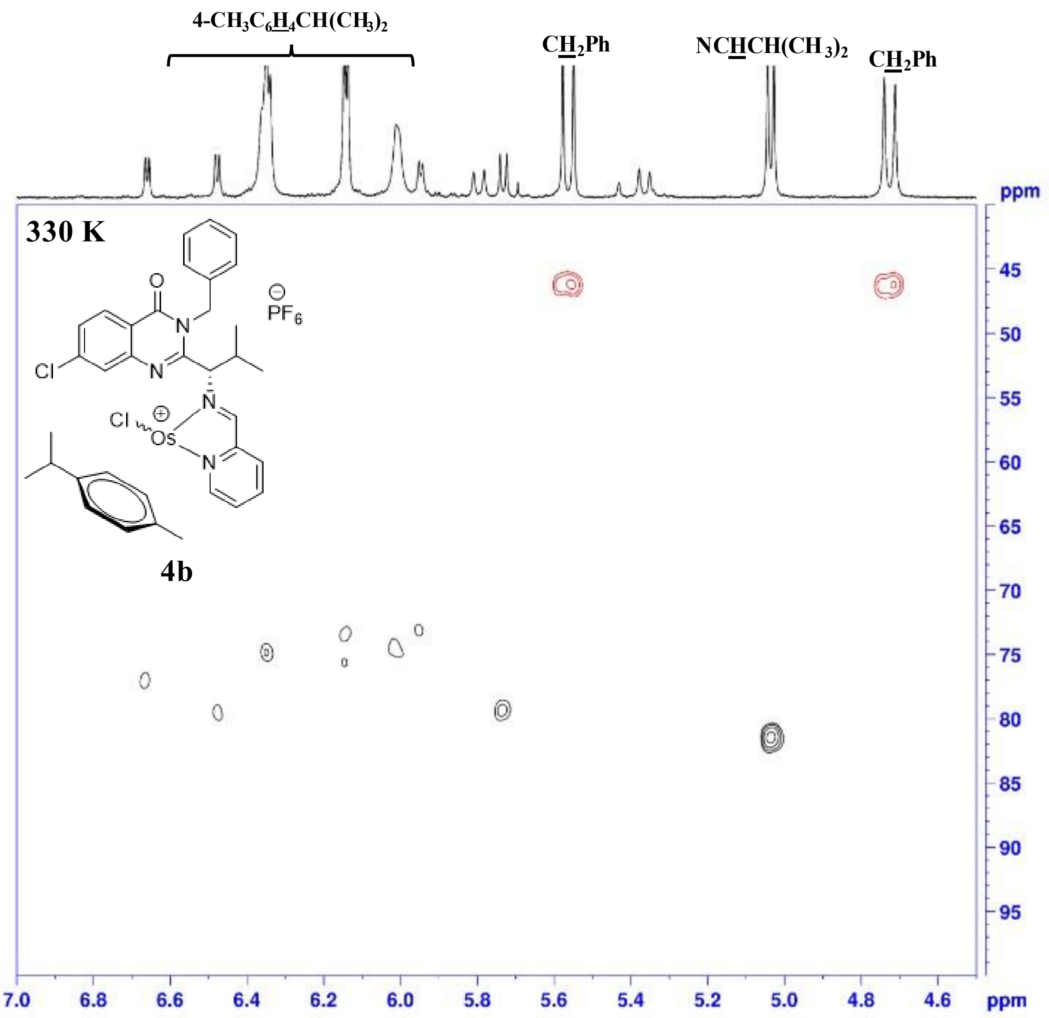

Figure S8. ^{1}H-^{13}C HSQC NMR of **4b** in DMSO-d_6 recorded at 330K.

HPLC-MS analysis

Peak#	Ret. Time	Area	Area%
1	0.755	3132	1.444
2	3.502	36327	16.752
3	5.223	177390	81.803
Total		216849	100.000

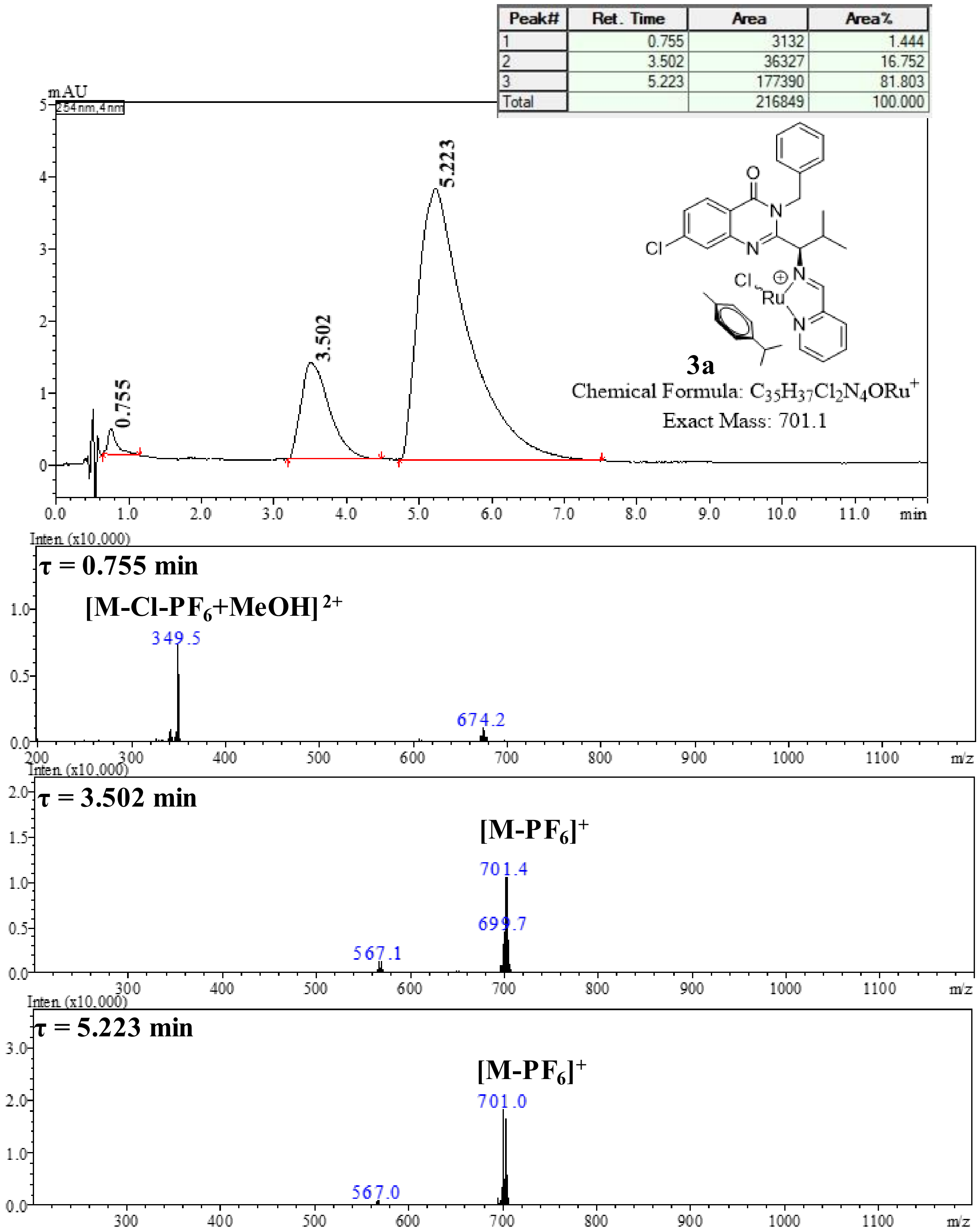

Figure S9. HPLC-MS analysis of **3a**

Peak#	Ret. Time	Area	Area%
1	0.755	1052	0.689
2	3.399	29517	19.346
3	5.076	122010	79.965
Total		152579	100.000

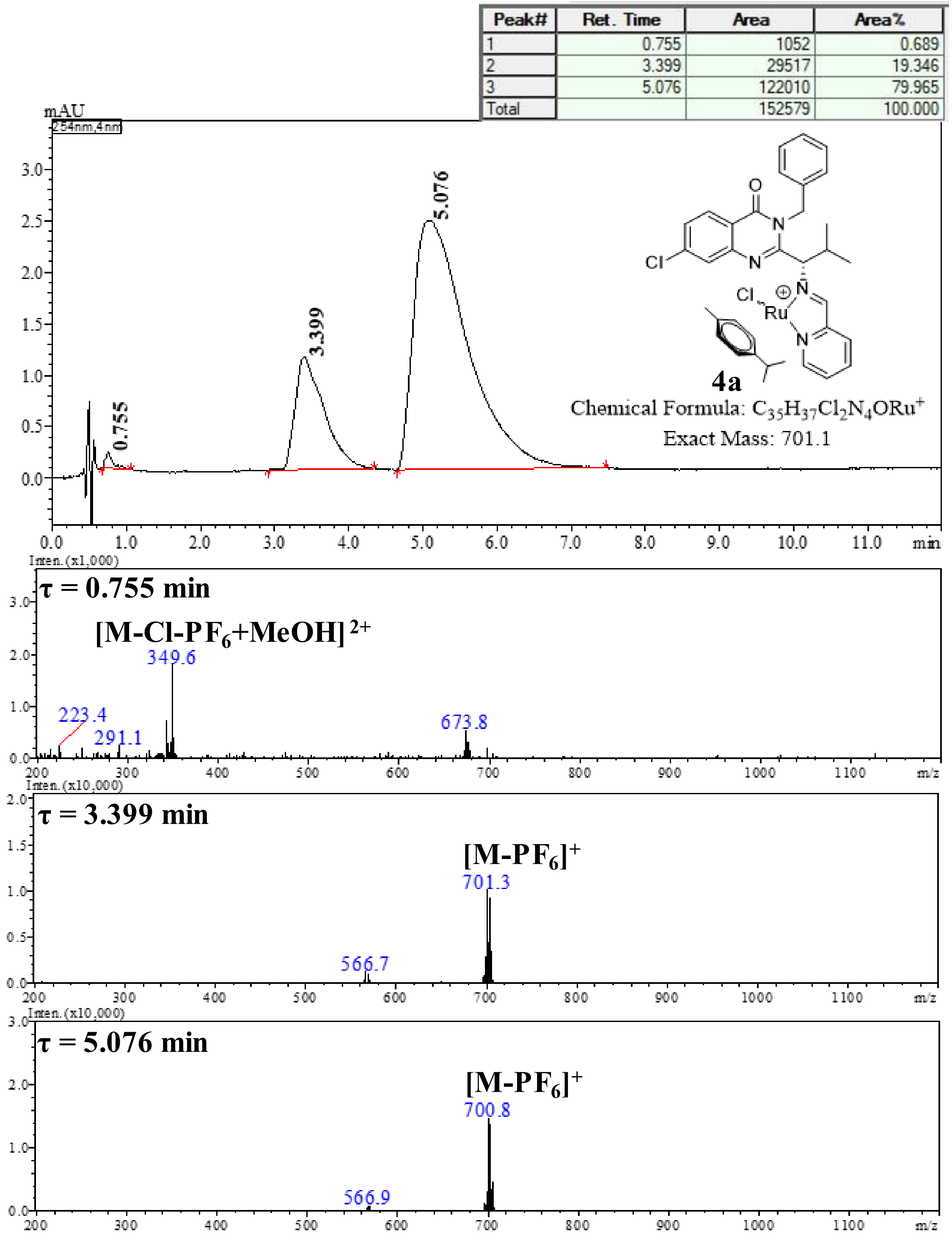

Figure S10. HPLC-MS analysis of **4a**

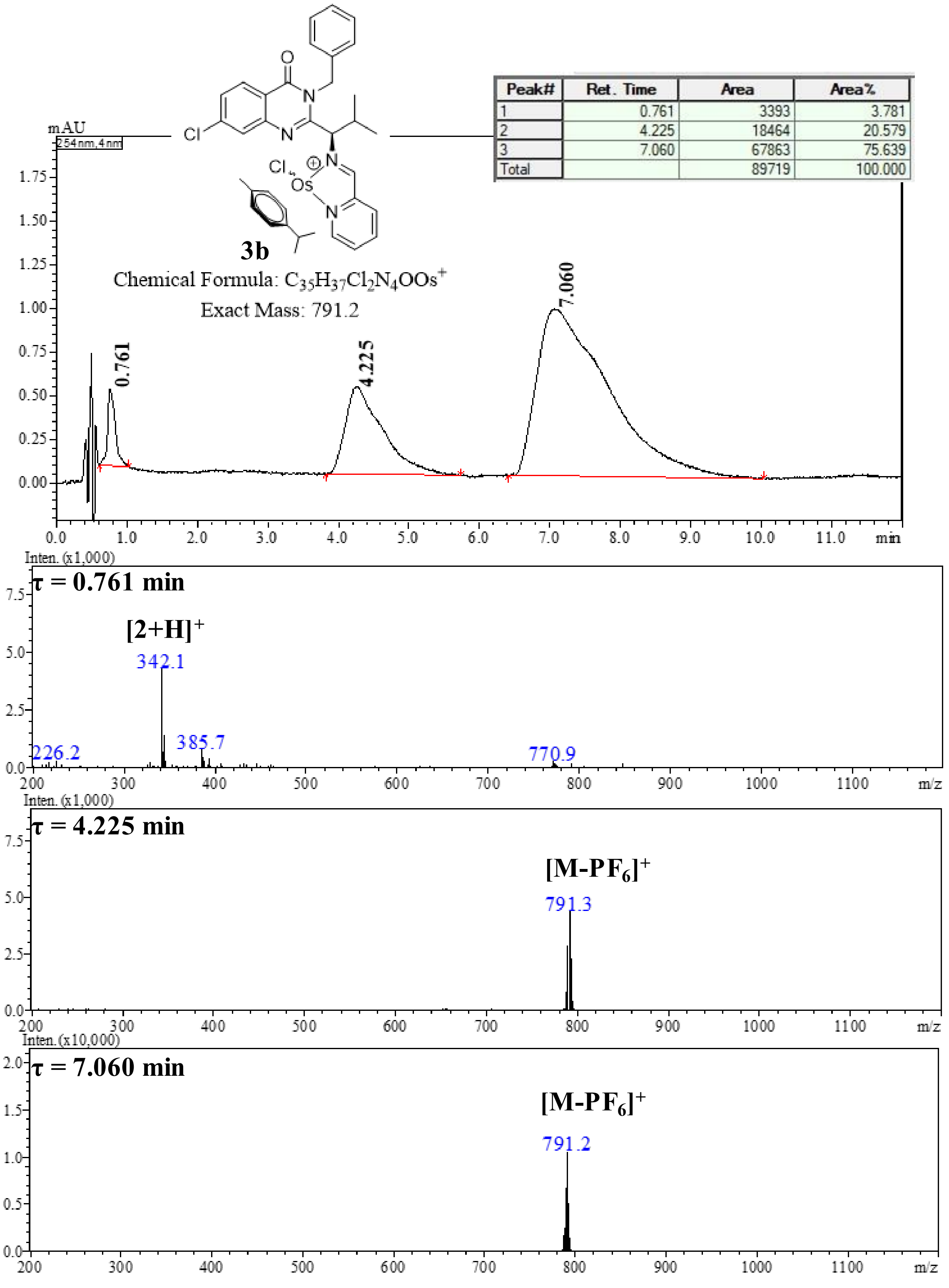

Peak#	Ret. Time	Area	Area%
1	0.761	3393	3.781
2	4.225	18464	20.579
3	7.060	67863	75.639
Total		89719	100.000

Figure S11. HPLC-MS analysis of **3b**

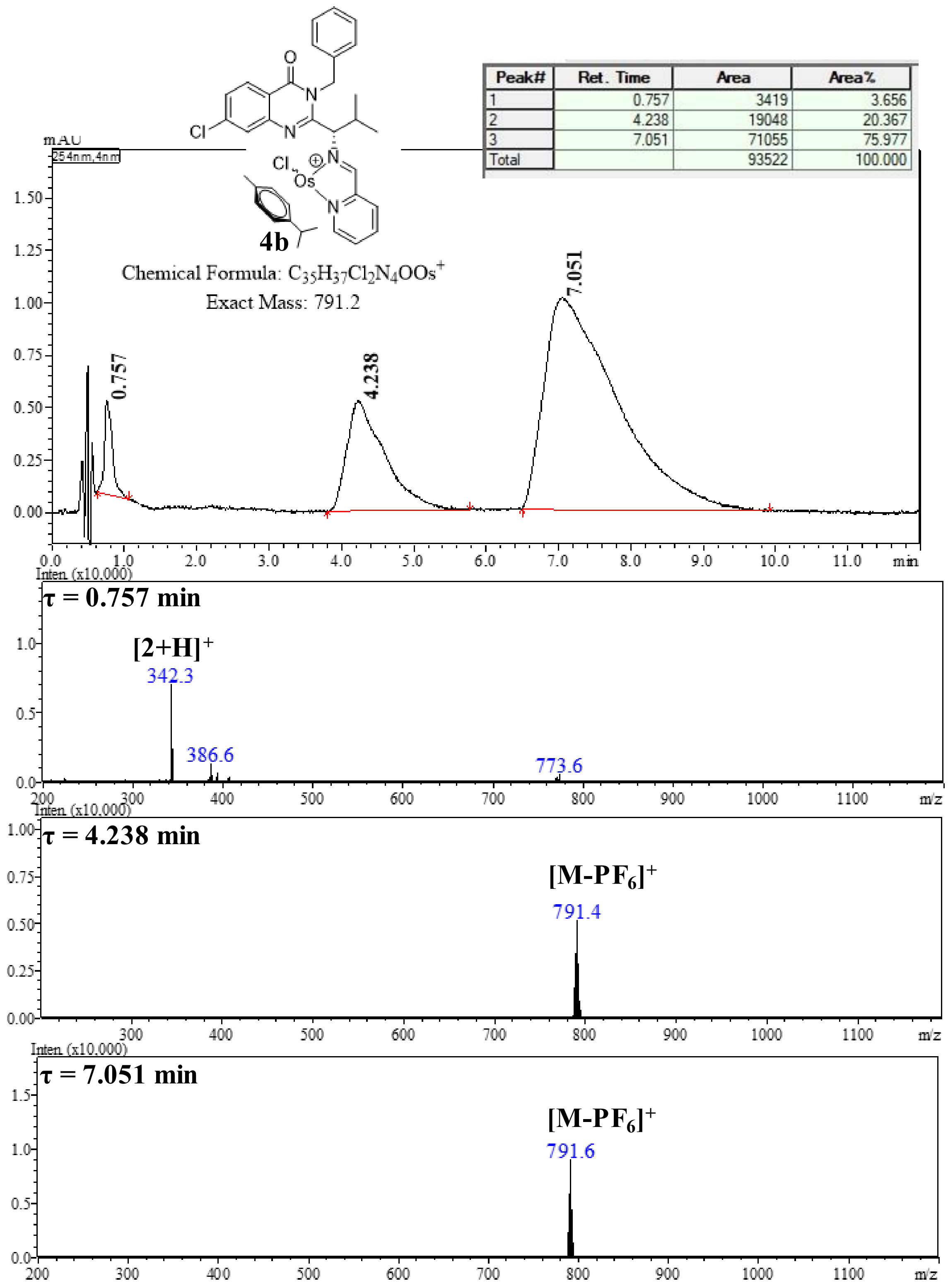

Peak#	Ret. Time	Area	Area%
1	0.757	3419	3.656
2	4.238	19048	20.367
3	7.051	71055	75.977
Total		93522	100.000

Figure S12. HPLC-MS analysis of **4b**

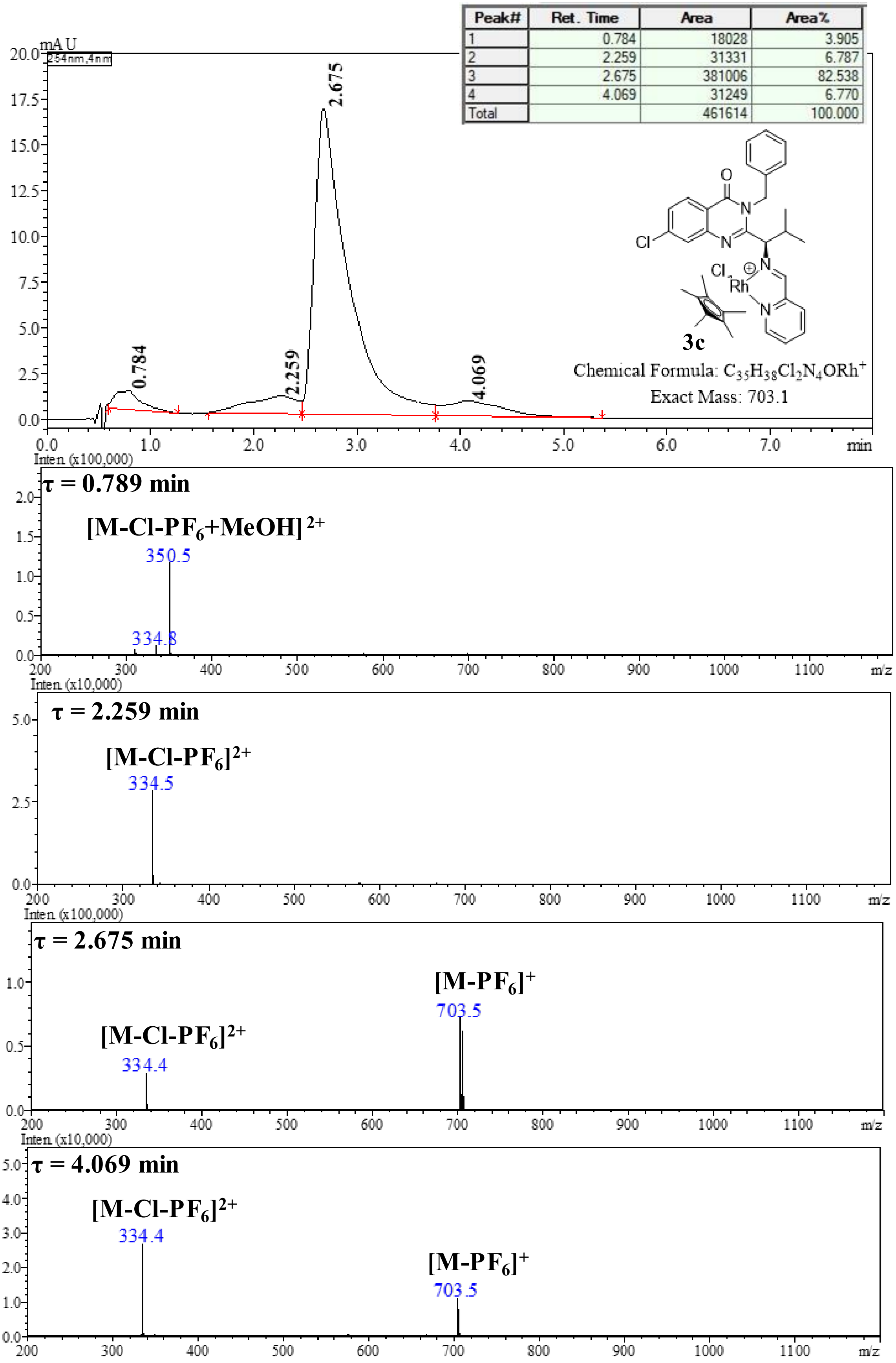

Peak#	Ret. Time	Area	Area%
1	0.784	18028	3.905
2	2.259	31331	6.787
3	2.675	381006	82.538
4	4.069	31249	6.770
Total		461614	100.000

Figure S13. HPLC-MS analysis of **3c**

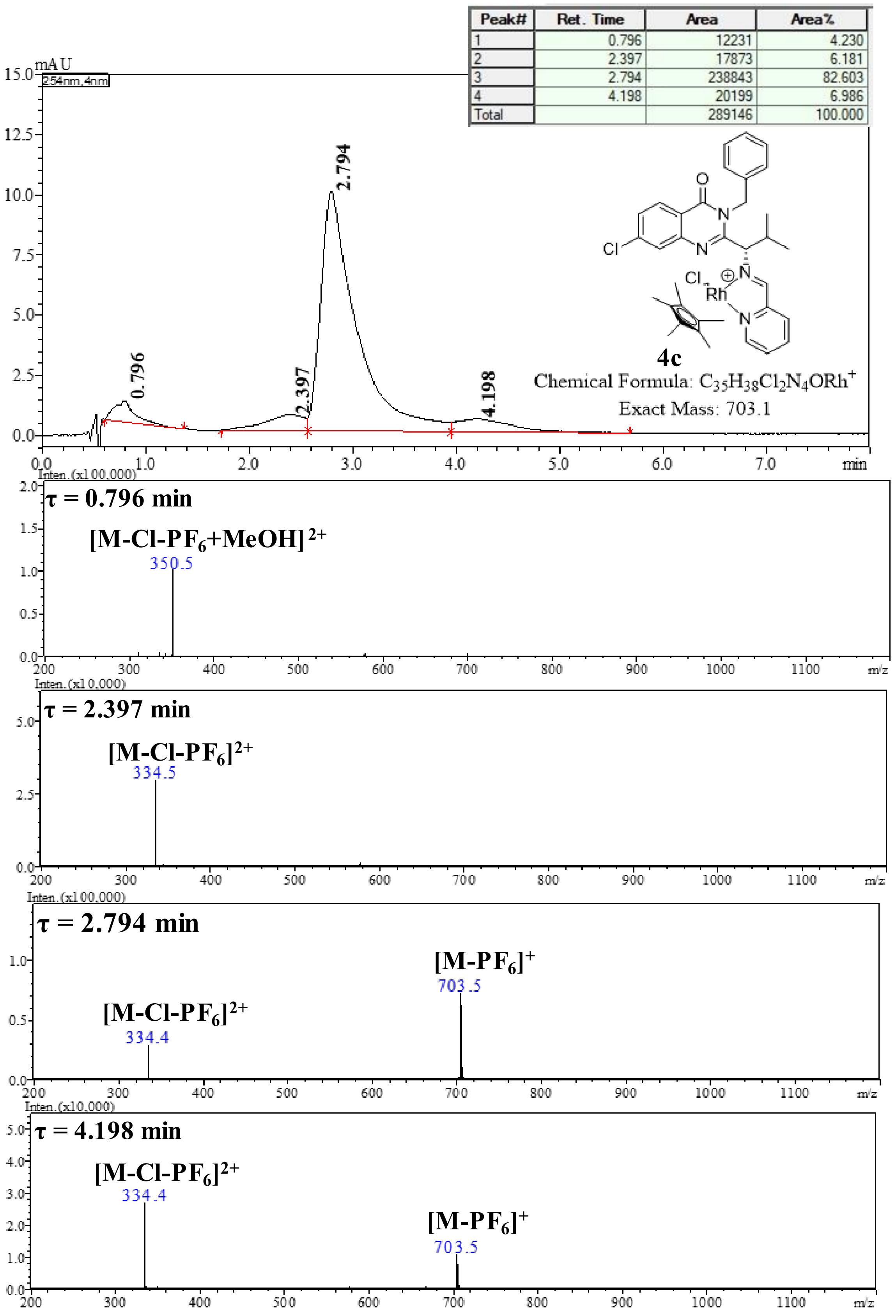

Peak#	Ret. Time	Area	Area%
1	0.796	12231	4.230
2	2.397	17873	6.181
3	2.794	238843	82.603
4	4.198	20199	6.986
Total		289146	100.000

Figure S14. HPLC-MS analysis of **4c**

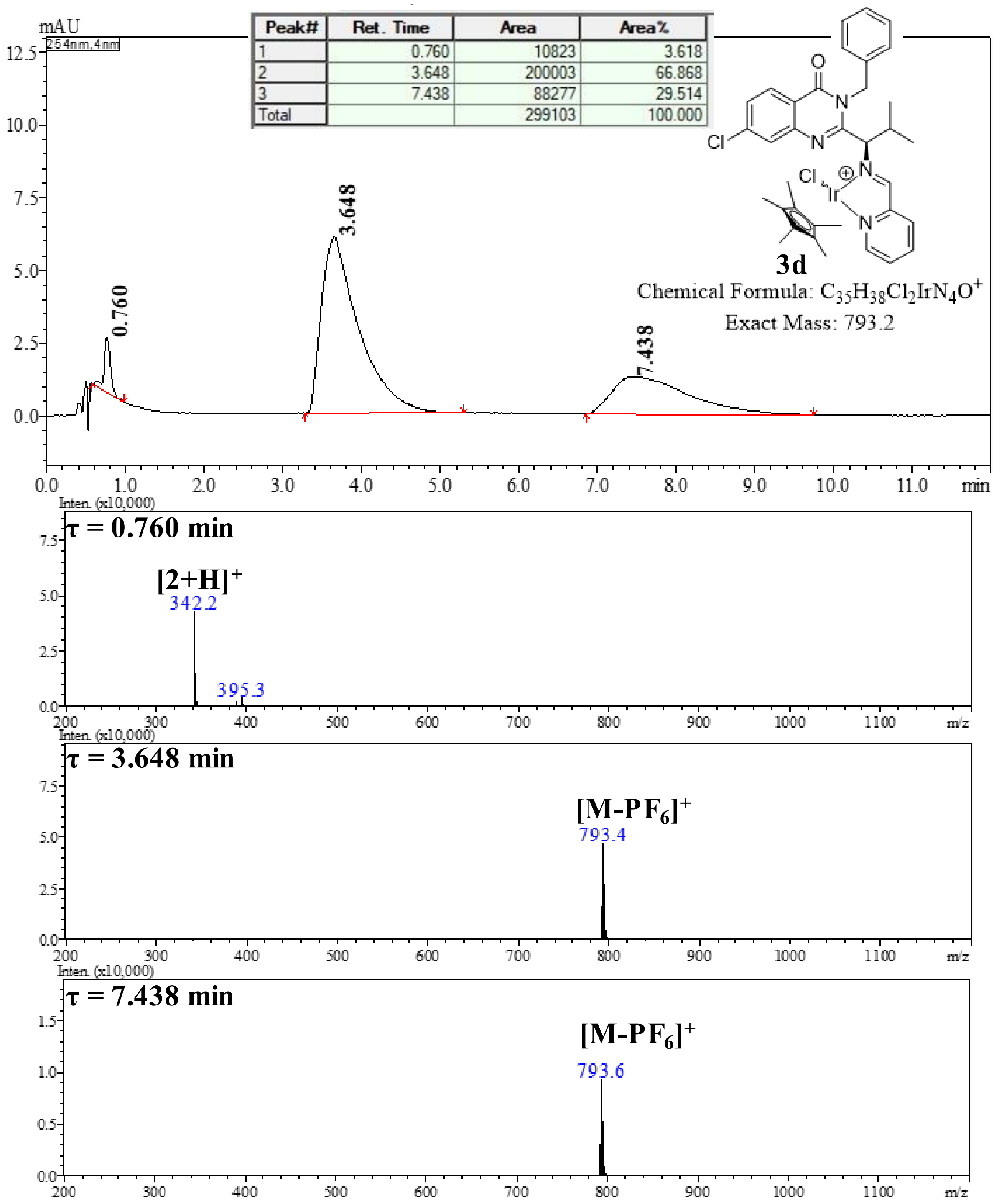

Peak#	Ret. Time	Area	Area%
1	0.760	10823	3.618
2	3.648	200003	66.868
3	7.438	88277	29.514
Total		299103	100.000

Figure S15. HPLC-MS analysis of **3d**

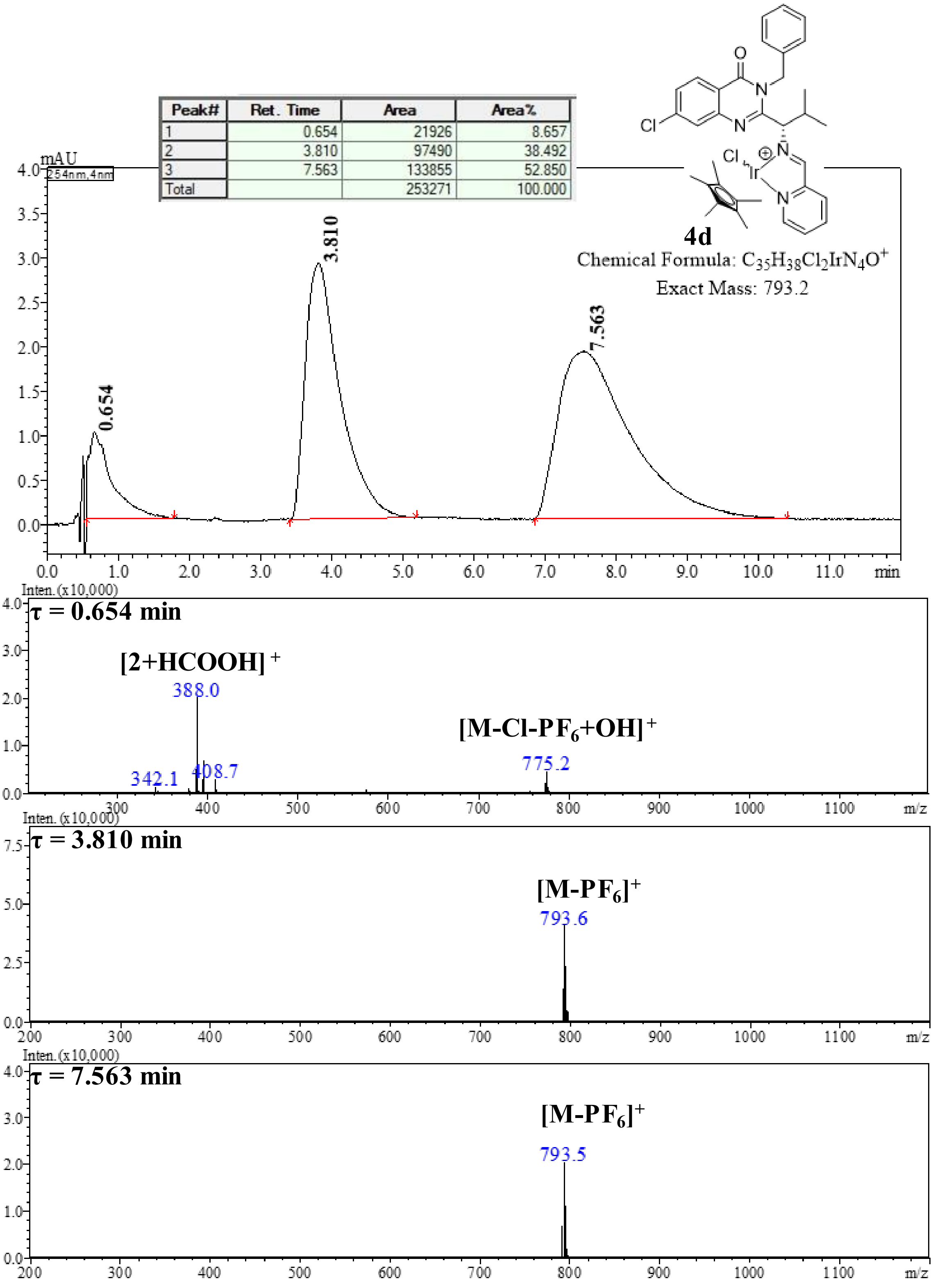

Peak#	Ret. Time	Area	Area%
1	0.654	21926	8.657
2	3.810	97490	38.492
3	7.563	133855	52.850
Total		253271	100.000

Figure S16. HPLC-MS analysis of **4d**

X-ray diffraction experimental details

Compound **4a**$^{S,S_{Ru}}$ has been investigated in terms of crystal structure and absolute configuration by the means of X-ray diffraction. The diffraction data were collected using a series of ω-scans on a four-circle SuperNova diffractometer utilizing a CuKα rotating anode, CCD-type Atlas detector, and Oxford Cryosystem cooling device. Clear honey-yellow block crystal sized 115 x 83 x 40 µm was mounted on a capillary using a trace amount of parabar oil and cooled to 100K. Data collection and refinement were performed using CrysAlisPro version 1.171.40.84a[3]. Numerical absorption correction based on gaussian integration as well as an empirical correction using spherical harmonics were performed via SCALE3 ABSPACK algorithm[3]. Absolute structure was determined using 7446 quotients, yielding the value of Flack parameter equal -0.0123(18)[4].

Initial model was obtained using a dual-space algorithm in SHELXT[5] and further refined assuming independent atom model by the means of least-squares refinement in SHELXL[6]. Apart from the disordered pentane molecule, all non-hydrogen atoms were refined anisotropically without restraints nor constraints. Hydrogen atoms were initially placed in idealized positions and afterwards the methyl groups were allowed to rotate freely. The displacement of all hydrogen atoms was refined with riding approximation, with U_{iso} fixed to 120% / 150% of U_{eq} of parent methylene or methine / methyl group. The crystal structure was refined within Olex2 1.5 GUI[7], whereas the visualization and further analysis were conducted using Mercury 2020.2.0[8].

Discussed crystal structure of **4a**$^{S,S_{Ru}}$ was deposited with CCDC and assigned deposition number 2207900. It can be accessed free of charge via www.ccdc.cam.ac.uk/data_request/cif,

by emailing data_request@ccdc.cam.ac.uk, or by contacting The Cambridge Crystallographic Data Centre, 12 Union Road, Cambridge CB2 1EZ, UK; fax: +44 1223 336033.

Table S1. Summary of the most important data and structure for **4a**$^{S,S_{Ru}}$**.**

**Compound**	**4a**$^{S,S_{Ru}}$
Empirical formula	$C_{35}H_{37}Cl_2N_4ORu,F_6P$, 0.5($C_5H_{12}$)
CCDC number	2207900
Formula weight	882.70
Crystal system	monoclinic
Space group	$P2_1$
a/Å	10.94530(10)
b/Å	31.3295(2)
c/Å	11.47270(10)
α/°	90
β/°	100.8570(10)
γ/°	90
Volume/Å^3	3863.69(6)
Z	4
ρ_{calc}/mg·mm^{-3}	1.517
F(000)	1804
μ/mm^{-1}	5.512

Max. transmission	0.922
Min. transmission	0.634
Absorption corr.	gaussian
Crystal color	clear dark yellow
Crystal habit	block
	0.115
	0.083
Crystal size/mm	0.040
R_{int}	0.0278
R_{sigma}	0.0183
Index ranges: h	<-13; 13>
k	<-39; 39>
l	<-14; 14>
Reflections collected	85464
2Θ range : max	74.773
min	2.821
Temperature/K	100.0(1)
X-ray wavelength/Å	1.54184
Independent refl. I > 2 σ(I)	15601

all	15726
Largest diff.:	
peak /e Å^{-3}	3.83
hole /e Å^{-3}	-1.27
Goodness-of-fit on F^2	1.05
Parameters	986
Data	15726
Restraints	16
R1 all data	0.0344
R1 [I>=2σ (I)]	0.0341
wR2 all data	0.0871
wR2 [I>=2σ (I)]	0.0874
Flack parameter	-0.0123(18)

Crystal structure validation

Mogul geometry check performed against the CSD database on the crystal structure of **4a**$^{S,S_{Ru}}$ has found no parameters to differ significantly from other deposited structures[8]. The only outlier found outside the statistical 2σ range was identified as the C–C–N angle (called henceforth ξ) between the iminium nitrogen and the quinazoline moiety in one of the molecules. Due to steric constraints, ξ assumes quite a low value of 107.8(3)°, whereas the only 5 other structures featuring this fragment in CSD exhibit ξ of 110.892(747)° , with minimum of 109.628° and

maximum of 111.494°. The value of ξ in the other independent molecule of $\mathbf{4a}^{S,S_{Ru}}$ equals 109.4(4) and is within the expected range.

General crystal structure validation performed for $\mathbf{4a}^{S,S_{Ru}}$ by checkCIF has raised no unexpected significant alerts[9]. While the conditions for three type "A" alerts were met, they stemmed from known experimental issues, i. e. disorder of the solvent pentane molecule and Fourier cutoff artifacts in close proximity to the ruthenium cation.

Detailed description of the crystal structure

Compound $\mathbf{4a}^{S,S_{Ru}}$ was crystallized from DCM/n-pentane mixture by slow evaporation in -20°C. The crystals grown in a chiral space group $P2_1$, with two $\mathbf{4a}^{S,S_{Ru}}$ cations, two PF_6^- anions and one disordered pentane moiety in the asymmetric unit. Both $\mathbf{4a}^{S,S_{Ru}\prime}$ and $\mathbf{4a}^{S,S_{Ru}\prime\prime}$ units assume a relatively similar geometry, with overlay RMSD equal 0.9274Å and maximum displacement of 2.146Å. The largest differences come from a different orientation of the chloride/p-cymene ligands as well as rotation of the phenyl group. With these moieties disregarded, the RMSD and displacement drop to 0.452Å and 0.834Å, respectively. The overlays of $\mathbf{4a}^{S,S_{Ru}\prime}$ and $\mathbf{4a}^{S,S_{Ru}\prime\prime}$ were presented in Figure S20.

While no theoretical calculations were performed for this system due to its size and content, the crystal structure of $\mathbf{4a}^{S,S_{Ru}}$ appears to be stabilized mostly by a mix of coulombic and weak directional interactions. Apart from a potential interaction between the *p*-cymene and pyridine moiety, no clear instances of π-stacking could be found. The disordered nature and large separation of the n-pentane from the rest of the structure suggest its small role in the overall stabilization. On the other hand, the hydrogen bonds, while all unconventional and thus potentially weak, are numerous. The PF_6^- ions appear to play a crucial role here, as not only do

they balance the positive charge of $\mathbf{4a}^{S,S_{Ru}}$ molecules, but also mediate 23 out of 35 independent hydrogen-involving contacts found automatically using geometric criteria (distance below sum of vdW radii, D–H...A angle below 100°).

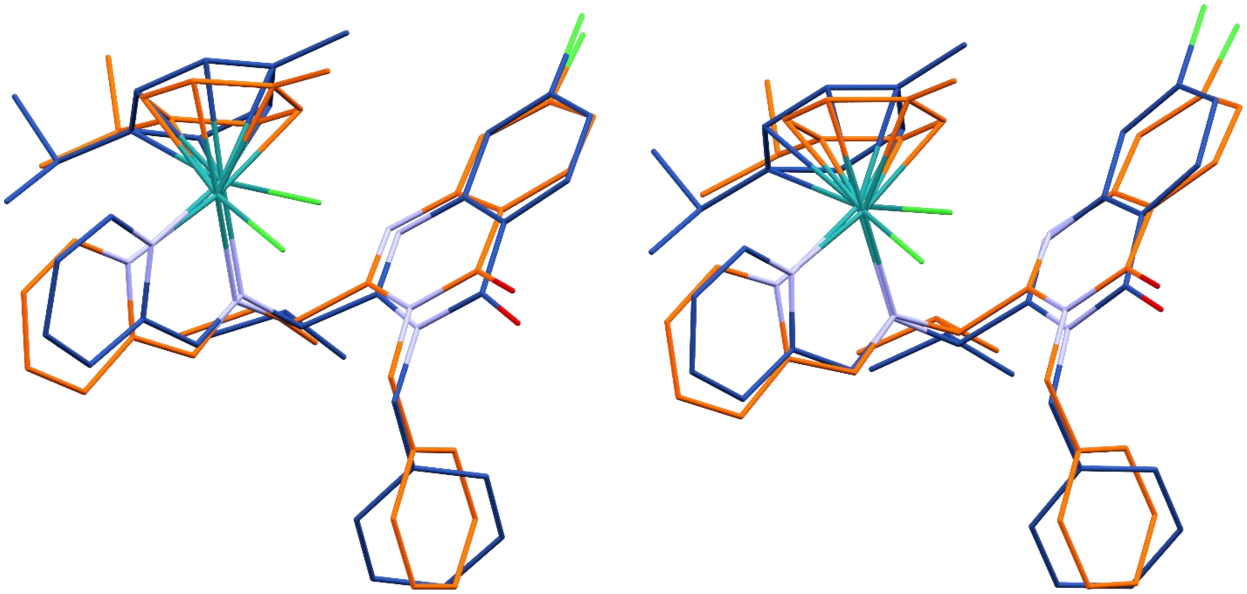

Figure S17. Overlays of two independent molecules of $\mathbf{4a}^{S,S_{Ru}}$, $\mathbf{4a}^{S,S_{Ru}\prime}$ (blue) and $\mathbf{4a}^{S,S_{Ru}\prime\prime}$ (orange), found in the crystal lattice with side groups regarded (left) and disregarded (right).

Stability studies - UV-Vis spectra

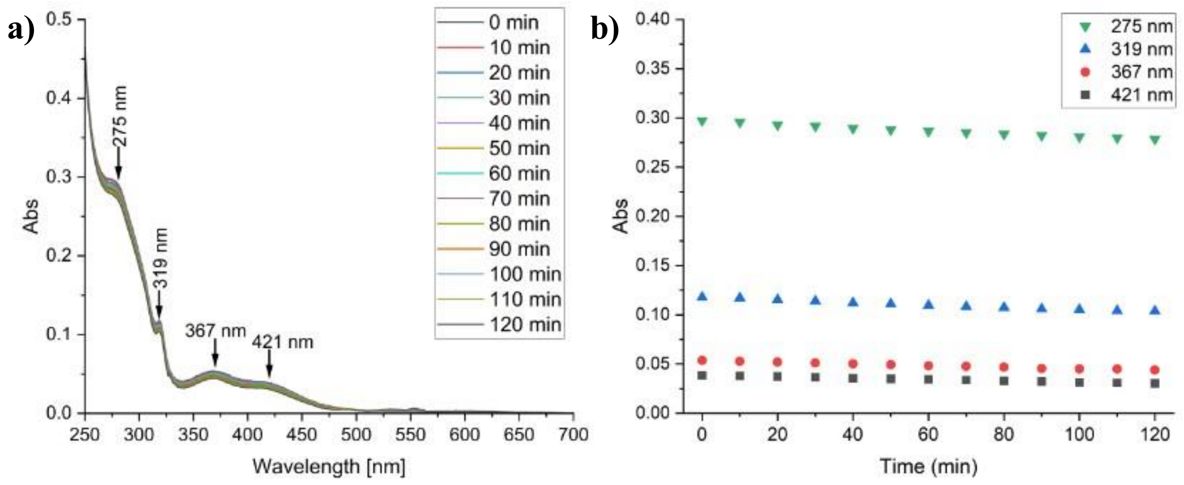

Figure S18. UV-Vis spectra of **3a** in DMSO-water solutions in the presence of L-cysteine (0.2 mM concentration) (a). The absorbance maxima value changes vs. time in the presence of L-cysteine (b).

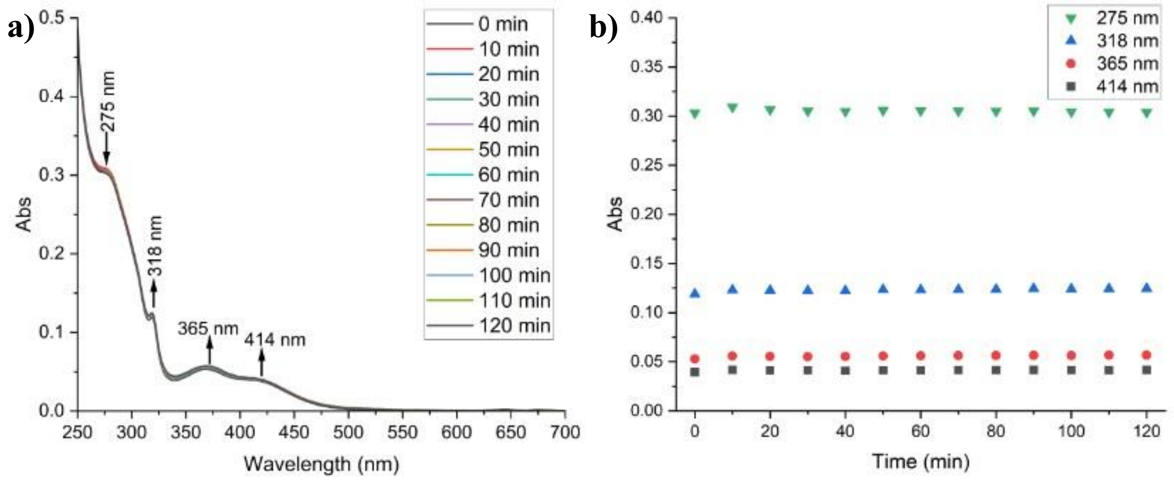

Figure S19. UV-Vis spectra of **3a** in DMSO-water solutions in the presence of L-histidine (0.2 mM concentration) (a). The absorbance maxima value changes vs. time in the presence of L-histidine (b)

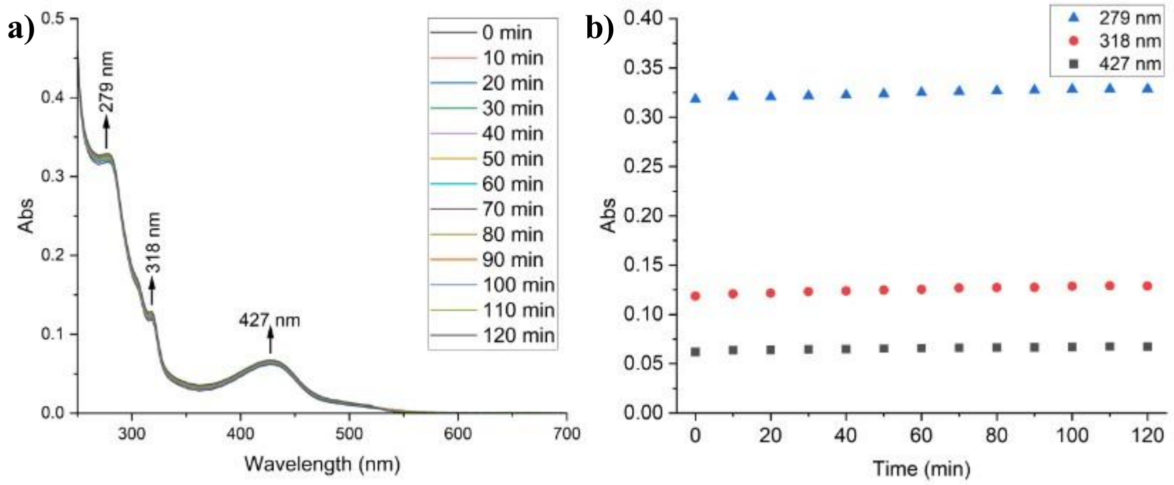

Figure S20. UV-Vis spectra of **3b** in DMSO-water solutions in the presence of L-cysteine (0.2 mM concentration) (a). The absorbance maxima value changes vs. time in the presence of L-cysteine (b).

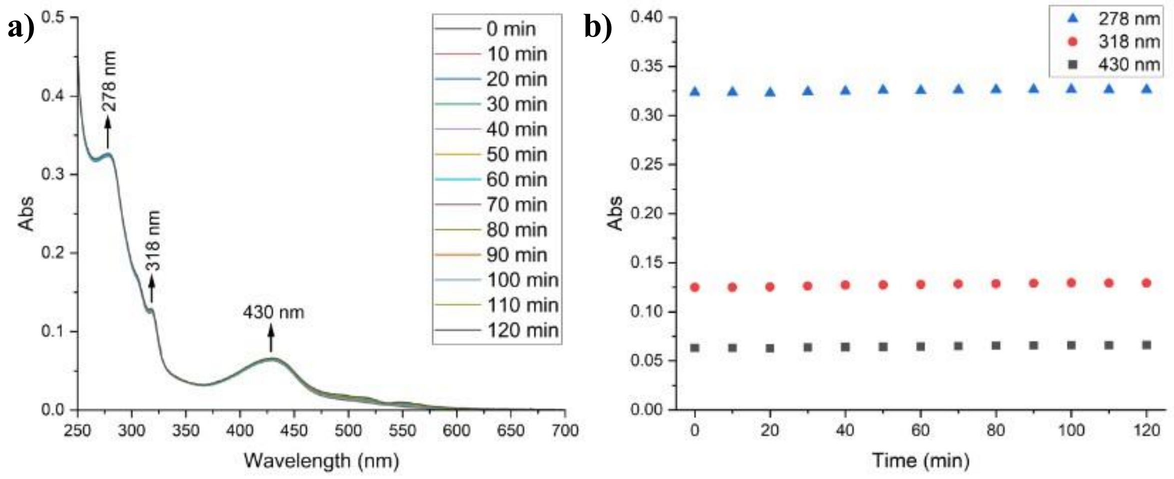

Figure S21. UV-Vis spectra of **3b** in DMSO-water solutions in the presence of L-histidine (0.2 mM concentration) (a). The absorbance maxima value changes vs. time in the presence of L-histidine (b).

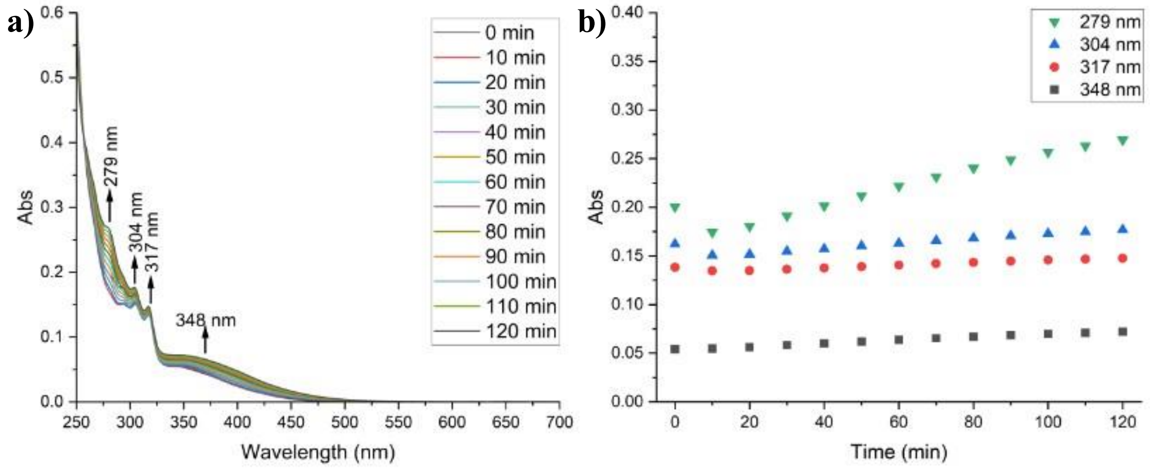

Figure S22. UV-Vis spectra of **3c** in DMSO-water solutions in the presence of L-cysteine (0.2 mM concentration) (a). The absorbance maxima value changes vs. time in the presence of L-cysteine (b).

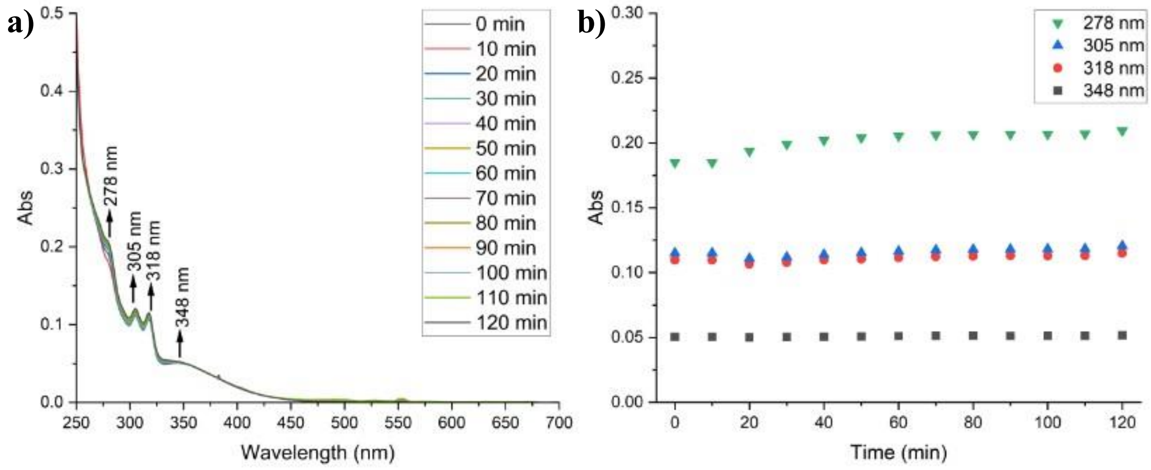

Figure S23. UV-Vis spectra of **3c** in DMSO-water solutions in the presence of L-histidine (0.2 mM concentration) (a). The absorbance maxima value changes vs. time in the presence of L-histidine (b).

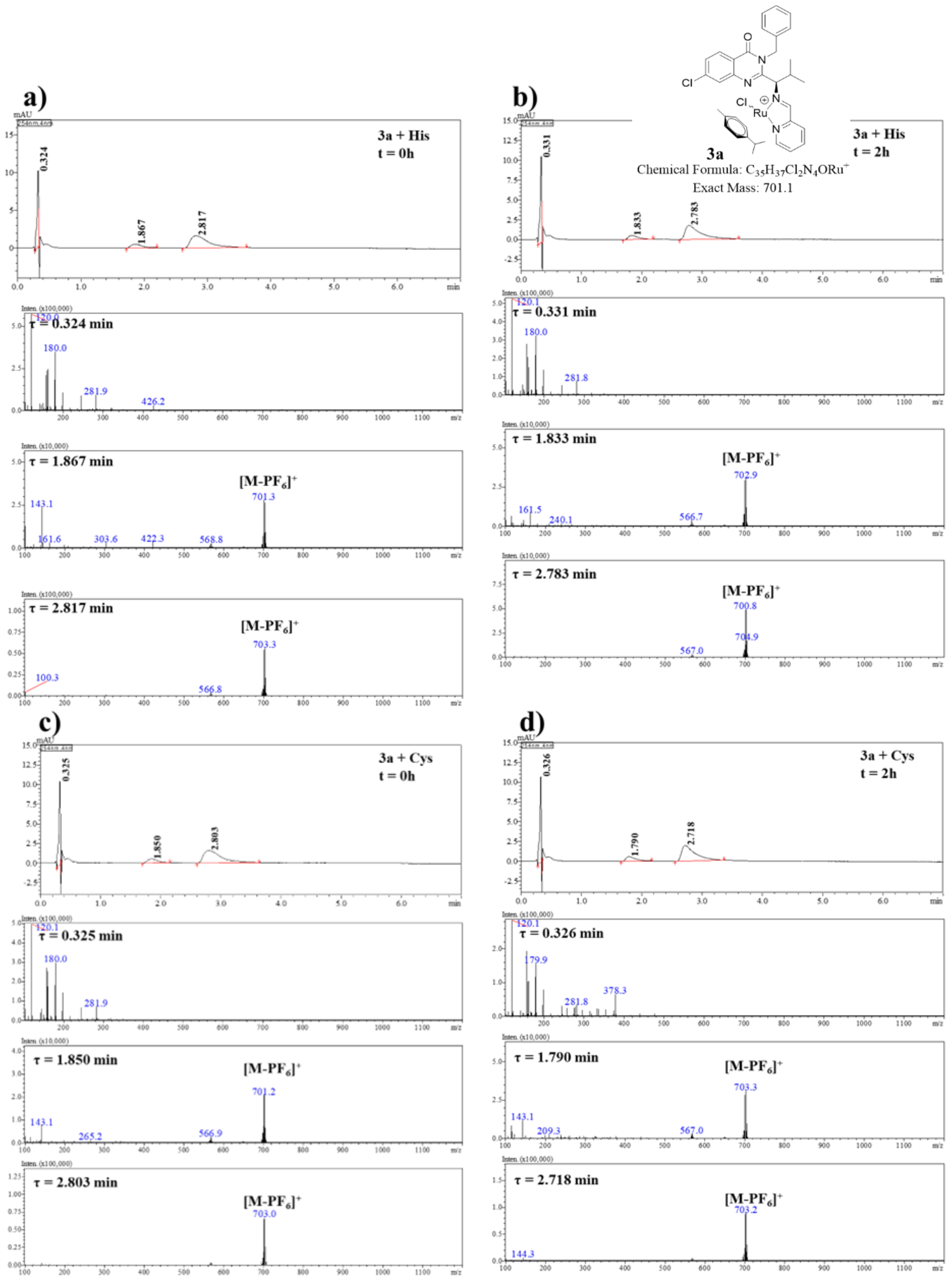

Figure S24. HPLC-MS analysis of **3a** in DMSO-water solutions in the presence of L-histidine (0.2 mM concentration) in t = 0 (a); in t = 2h (b) and in the presence of L-cysteine (0.2 mM concentration) in t = 0 (c); in t = 2h (d).

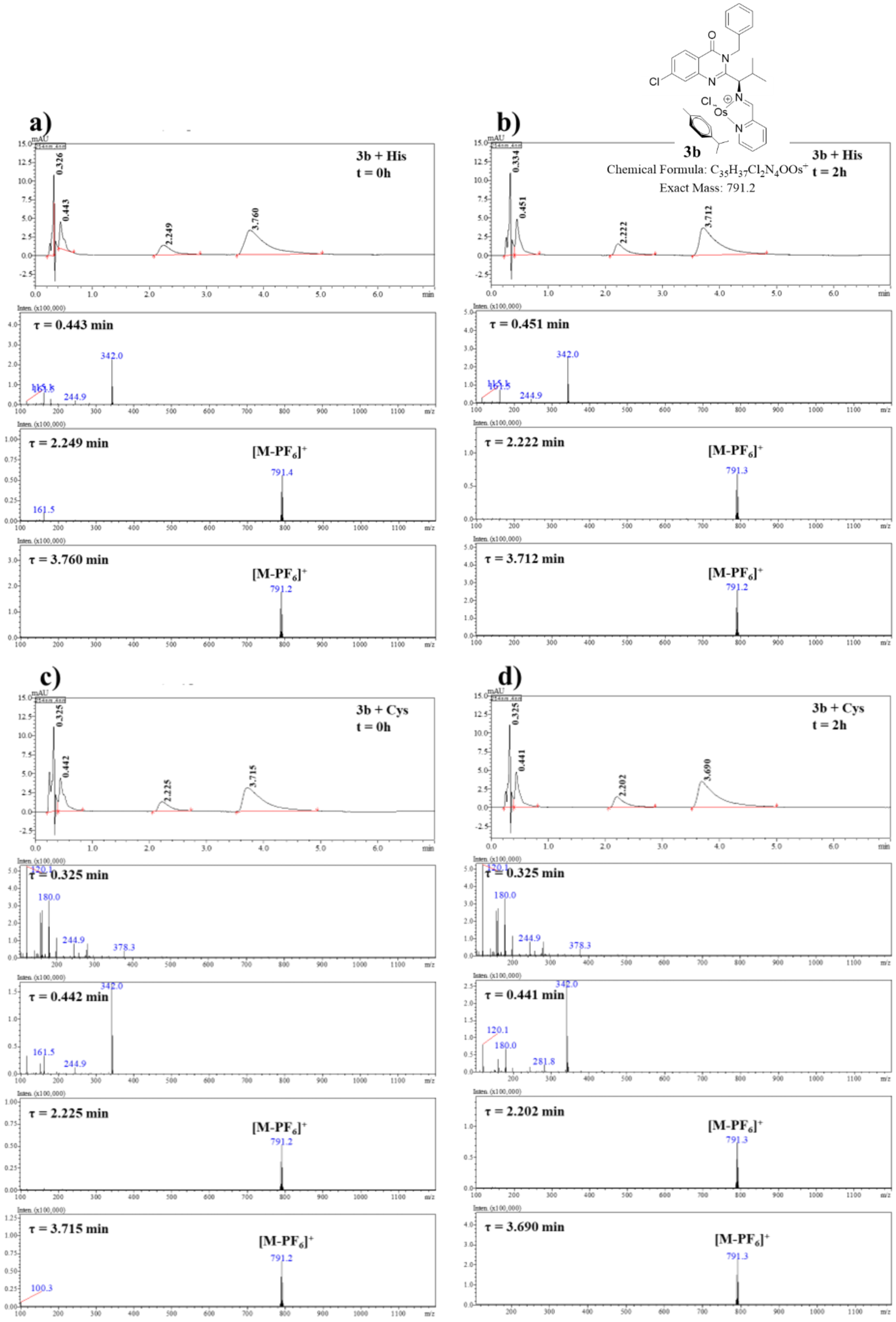

Figure S25. HPLC-MS analysis of **3b** in DMSO-water solutions in the presence of L-histidine (0.2 mM concentration) in t = 0 (a); in t = 2h (b) and in the presence of L-cysteine (0.2 mM concentration) in t = 0 (c); in t = 2h (d).

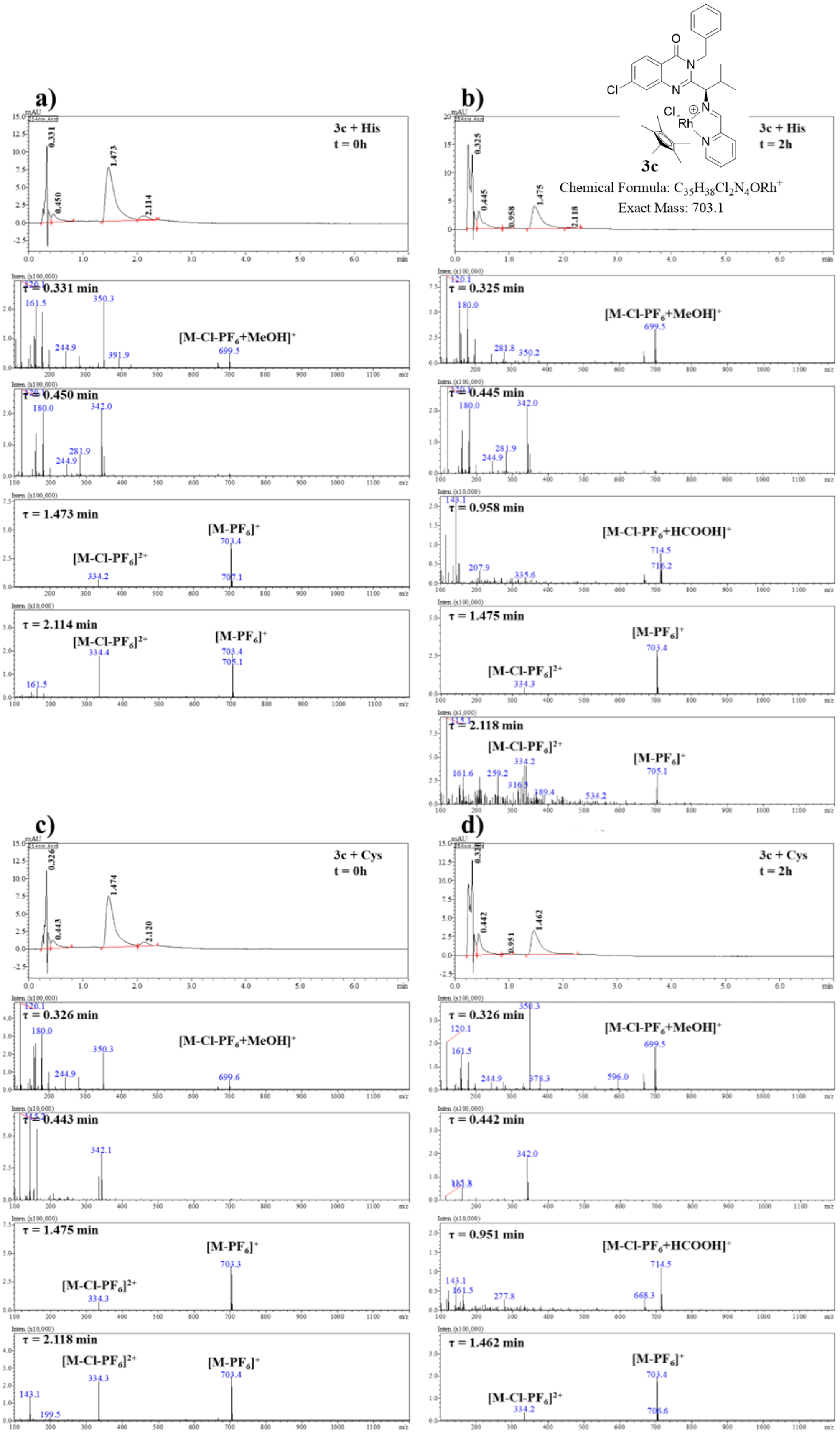

Figure S26. HPLC-MS analysis of **3c** in DMSO-water solutions in the presence of L-histidine (0.2 mM concentration) in t = 0 (a); in t = 2h (b) and in the presence of L-cysteine (0.2 mM concentration) in t = 0 (c); in t = 2h (d).

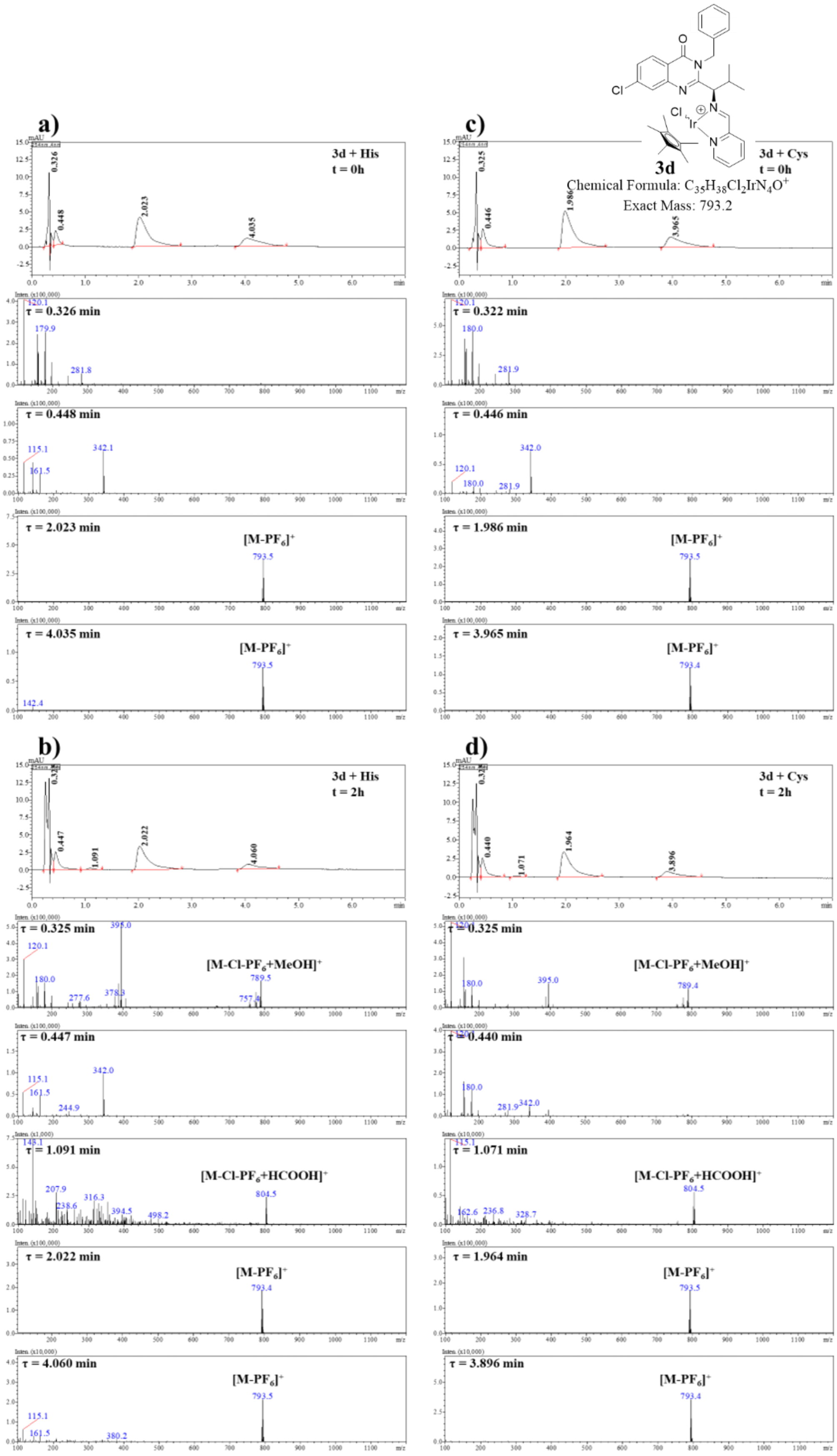

Figure S27. HPLC-MS analysis of **3d** in DMSO-water solutions in the presence of L-histidine (0.2 mM concentration) in t = 0 (a); in t = 2h (b) and in the presence of L-cysteine (0.2 mM concentration) in t = 0 (c); in t = 2h (d).

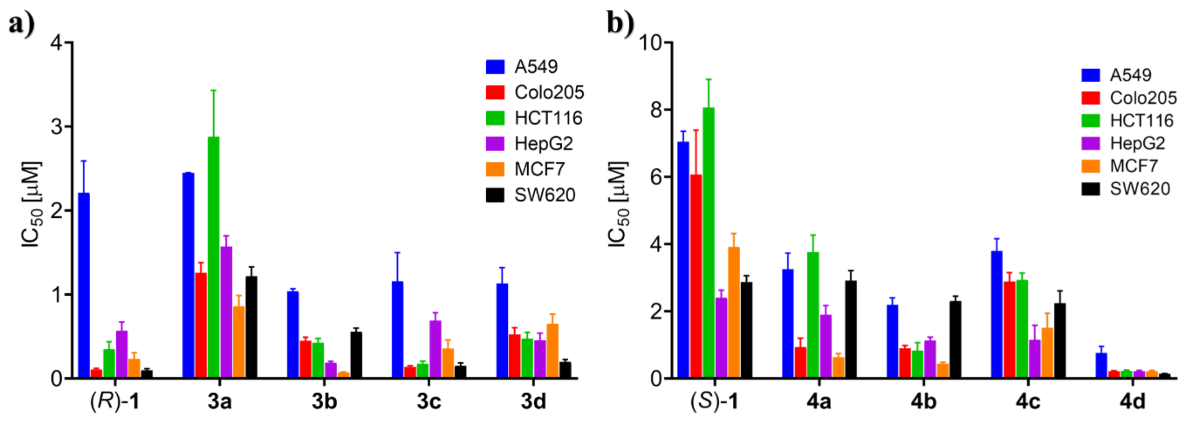

Figure S28. Graphical presentation of antiproliferative activity of: a) (*R*)-**1** and the organometallic complexes **3a-d**; b) (*S*)-**1** and organometallic analogues **4a-4d** in human cancer cell lines expressed as IC_{50} values. IC_{50} values are presented along with the corresponding 95% confidence intervals (n = 3).

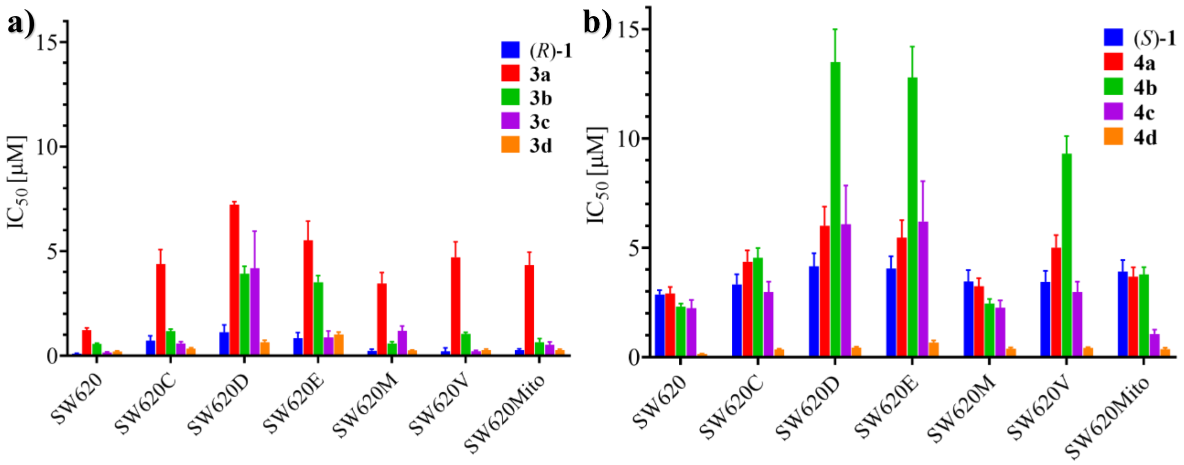

Figure S29. Graphical presentation of antiproliferative activity of: a) (*R*)-**1** and the organometallic complexes **3a-d**; b) (*S*)-**1** and organometallic analogues **4a-4d** in multidrug resistant (MDR) cancer cell lines expressed as IC_{50} values. IC_{50} values are presented along with the corresponding 95% confidence intervals (n = 3).

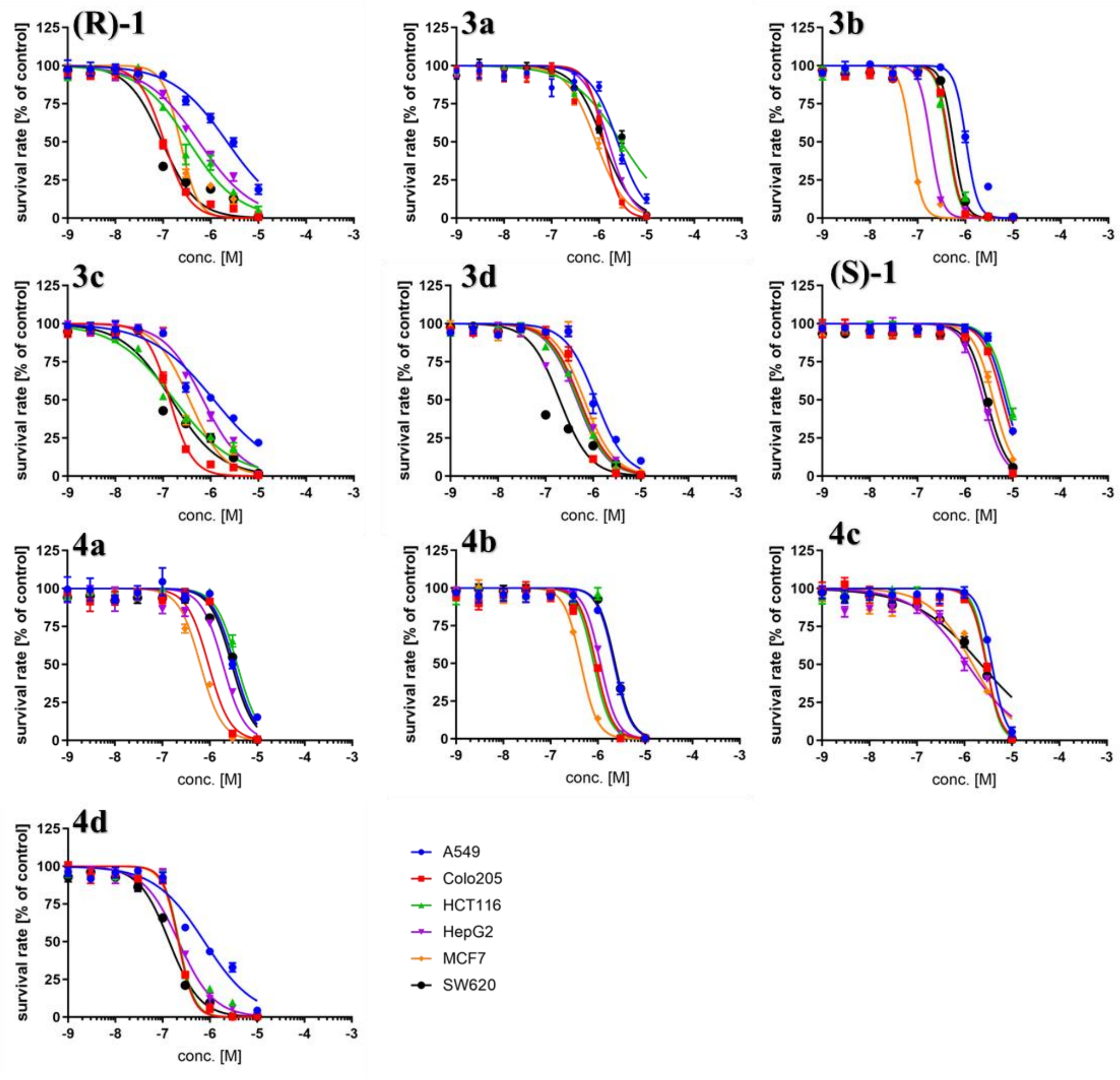

Figure S30. Inhibitory curves in human cancer cell lines.

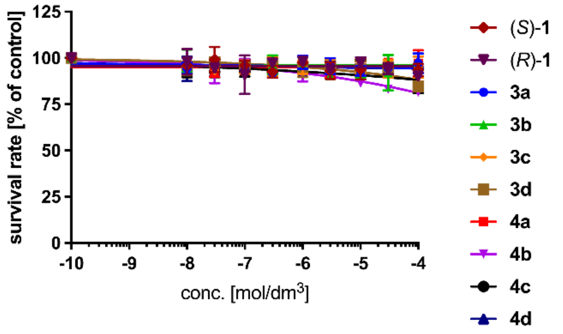

Figure S31. Inhibitory curves in normal MRC-5 cell line.

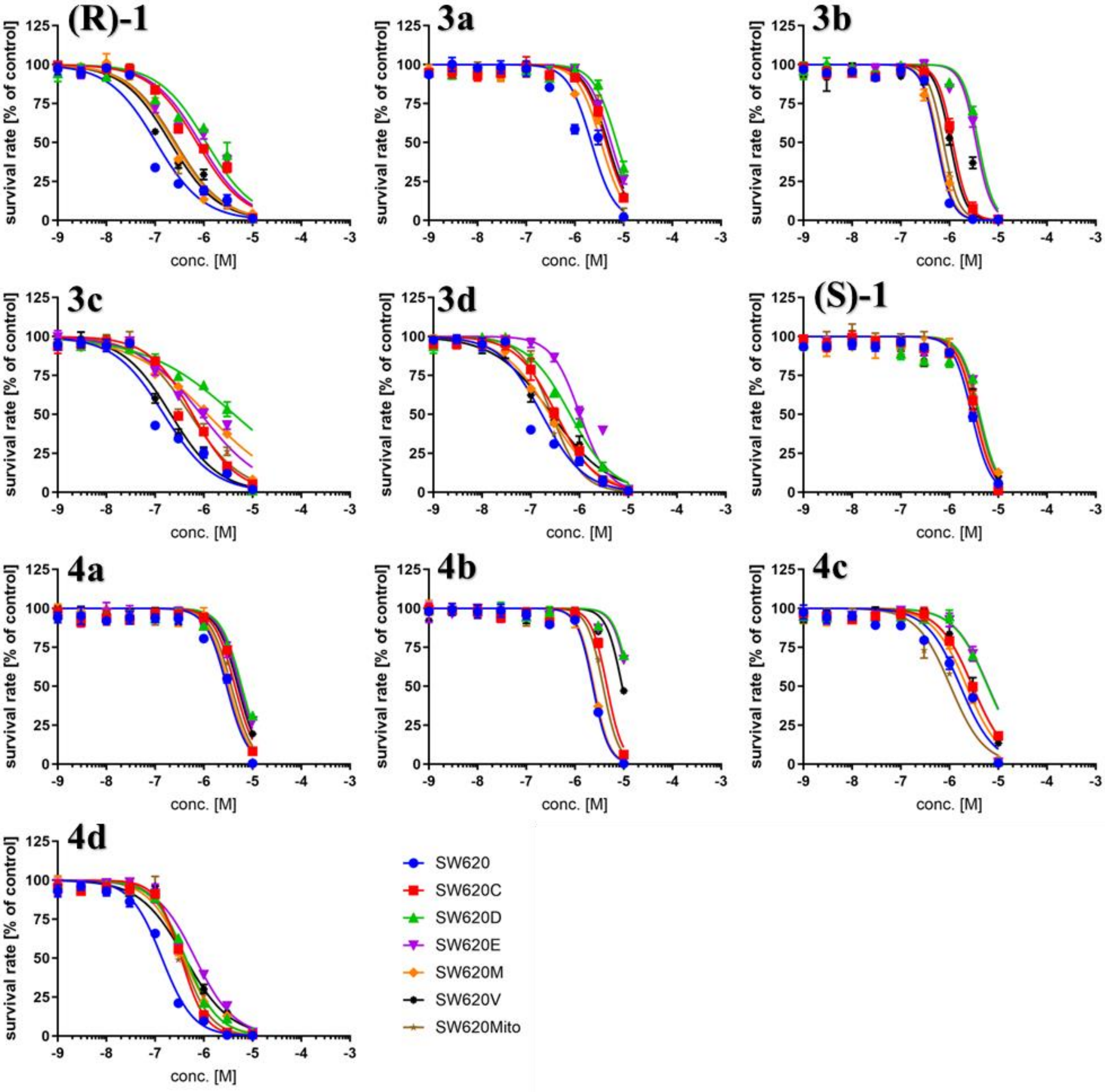

Figure S32. Inhibitory curves in multidrug resistant (MDR) cancer cell lines.

Table S2. Cell cycle phase distribution for SW620 cells exposed for 24 h and 48 h to (*S*)-**1** and **(*R*)-1** and the corresponding synthesized metal complexes **3a–d** and **4a–d** at concentrations equal to the IC_{75} values of (*S*)-**1** and (*R*)-**1**, respectively. Data are presented as mean ± SEM, n = 3

	Cell cycle fractions/%					
	24 h			48 h		
**Compound**	G_1	S	G_2/M	G_1	S	G_2/M
Ctrl	62.4 ± 1.7	16.1 ± 0.4	21.3 ± 1.4	61.7 ± 1.3	15.8 ± 0.2	21.9 ± 0.7
DMSO	61.5 ± 0.9	15.6 ± 0.7	22.1 ± 1.0	62.0 ± 1.8	15.9 ± 0.7	22.0 ± 2.1
(*R*)-**1**	45.4 ± 1.0	17.2 ± 1.3	36.6 ± 2.2	36.2 ± 1.4	19.0 ± 0.6	42.1 ± 0.4
**3a**	51.9 ± 1.0	14.9 ± 0.6	32.5 ± 1.5	44.8 ± 0.7	14.3 ± 0.4	40.2 ± 1.1
**3b**	51.4 ± 1.2	12.5 ± 2.1	35.3 ± 1.4	41.4 ± 0.7	12.7 ± 1.1	45.0 ± 0.5
**3c**	33.9 ± 2.5	24.2 ± 1.4	40.9 ± 3.5	23.1 ± 1.7	20.8 ± 2.2	54.5 ± 1.8
**3d**	30.8 ± 0.8	25.4 ± 0.8	43.4 ± 1.5	24.3 ± 1.2	19.3 ± 0.4	55.9 ± 1.1
(*S*)-**1**	41.8 ± 1.2	19.5 ± 0.3	37.1 ± 1.2	35.2 ± 1.1	19.9 ± 0.4	42.5 ± 0.6
**4a**	52.8 ± 1.2	13.5 ± 0.4	33.3 ± 1.0	46.8 ± 0.7	13.5 ± 0.8	39.1 ± 0.3
**4b**	51.8 ± 0.2	15.0 ± 0.5	32.6 ± 0.3	41.8 ± 0.4	14.9 ± 0.4	42.7 ± 0.6
**4c**	52.6 ± 1.9	13.6 ± 0.3	32.7 ± 2.0	41.6 ± 1.0	13.7 ± 0.4	43.8 ± 1.4
**4d**	51.7 ± 1.2	14.3 ± 0.2	28.9 ± 0.4	44.1 ± 0.5	13.7 ± 0.4	40.6 ± 1.3

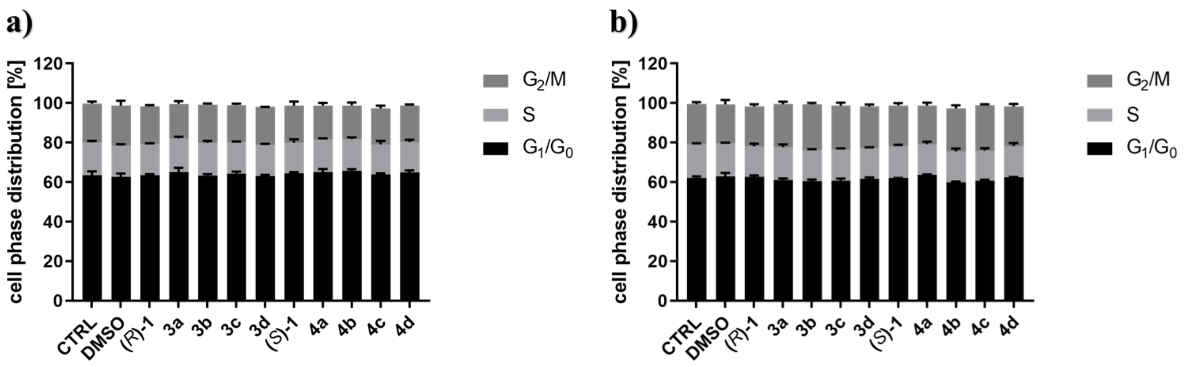

Figure S33. Graphical presentation of cell cycle distribution in SW620E cells: a) after 24; b) after 48 h.

NMR Spectra

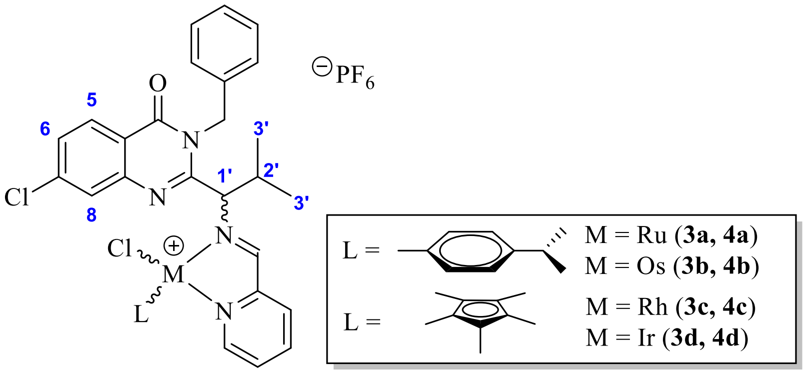

Figure S34. General structure of compounds **3a-4d**

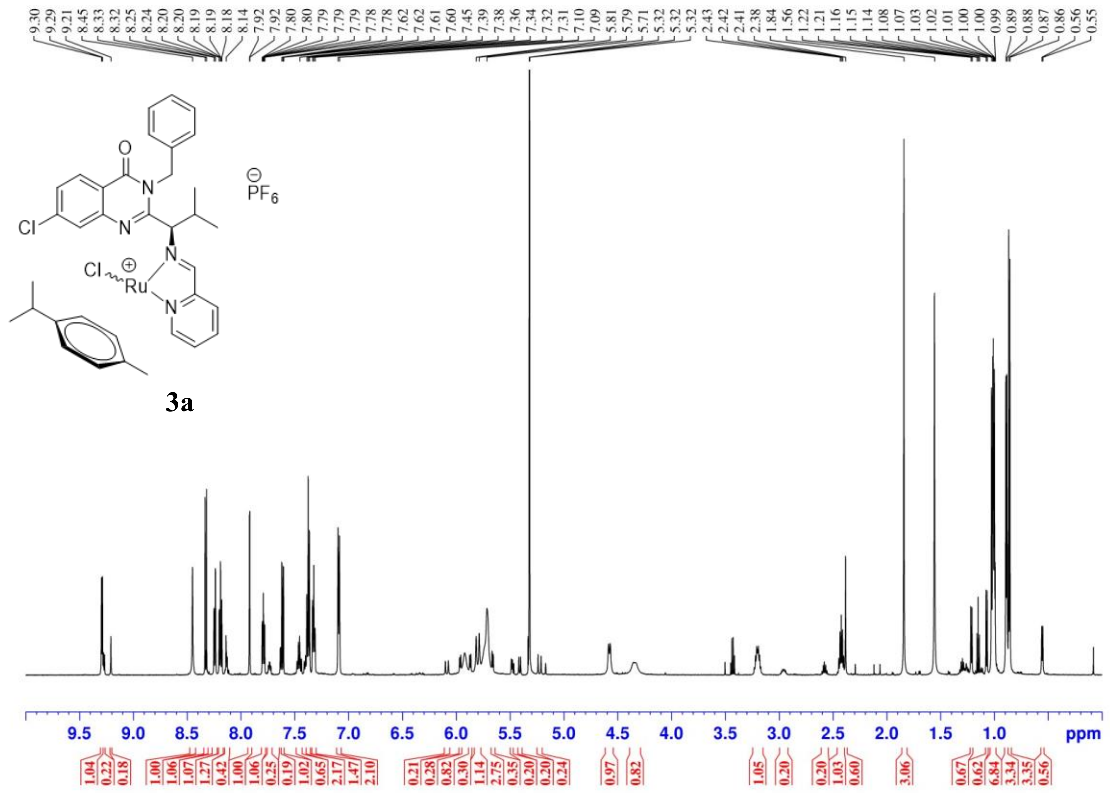

Figure S35. ^{1}H NMR spectra of **3a** in CD_2Cl_2

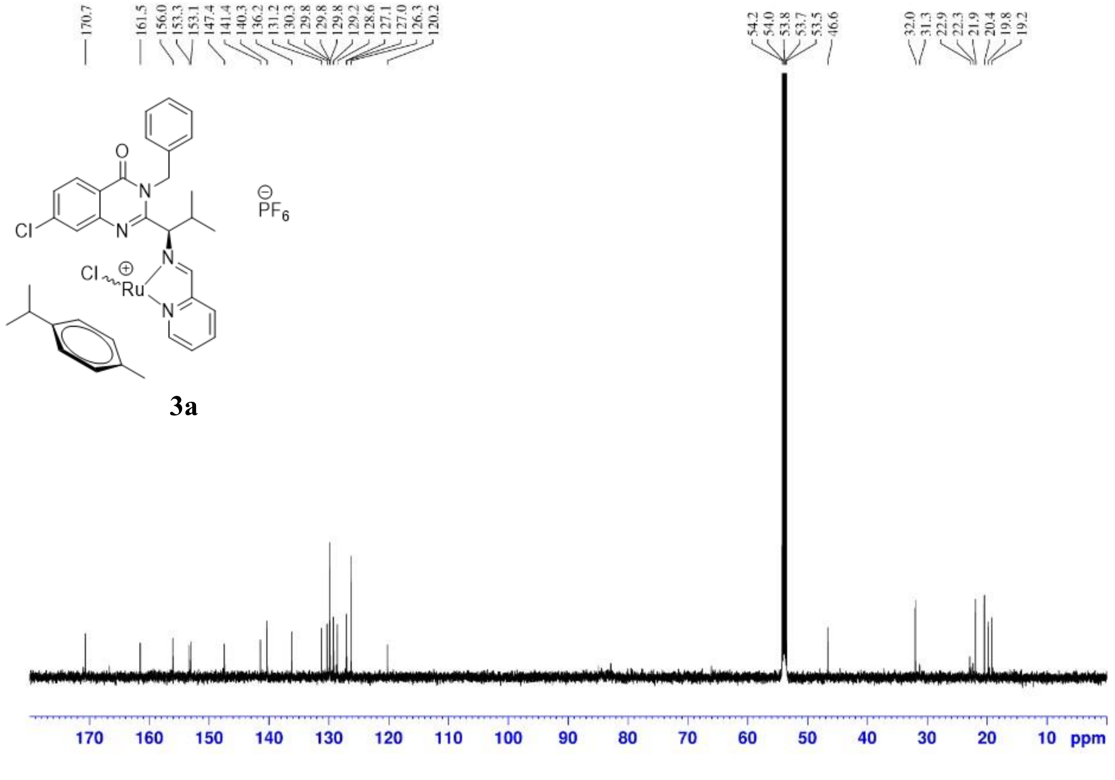

Figure S36. $^{13}C\{^{1}H\}$ NMR spectra of **3a** in CD_2Cl_2

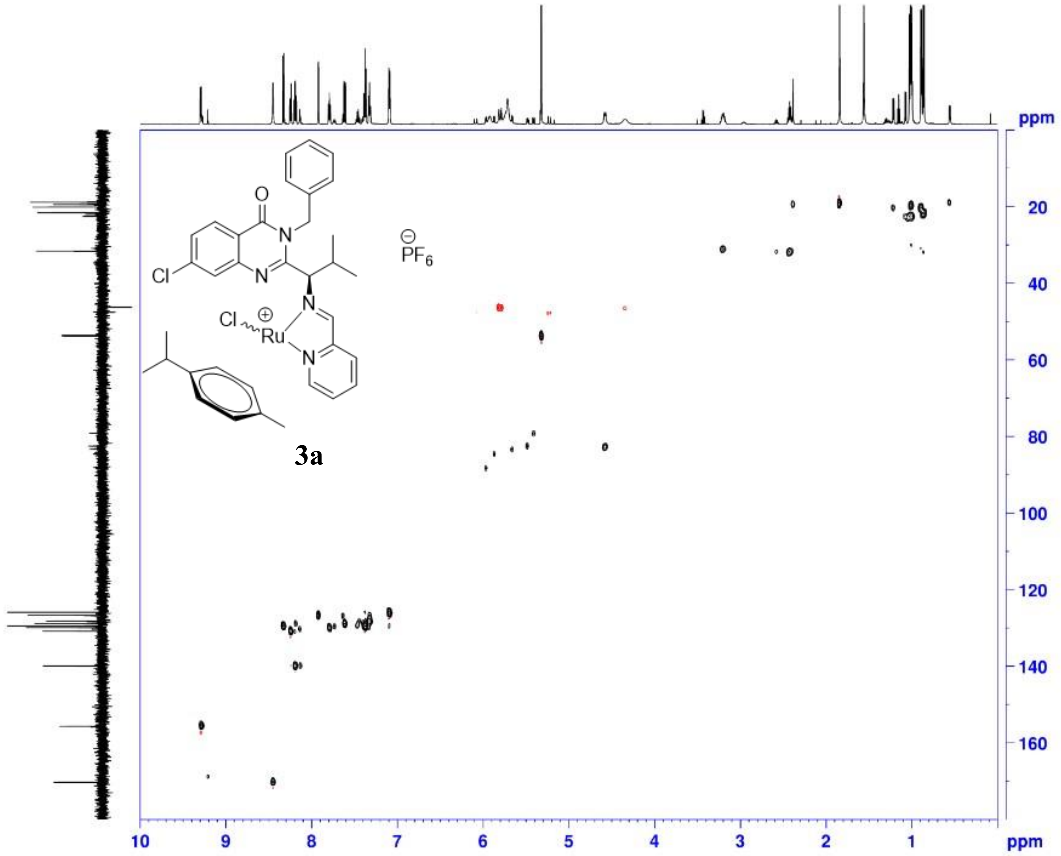

Figure S37. ^{1}H-^{13}C HSQC NMR spectra of **3a** in CD_2Cl_2

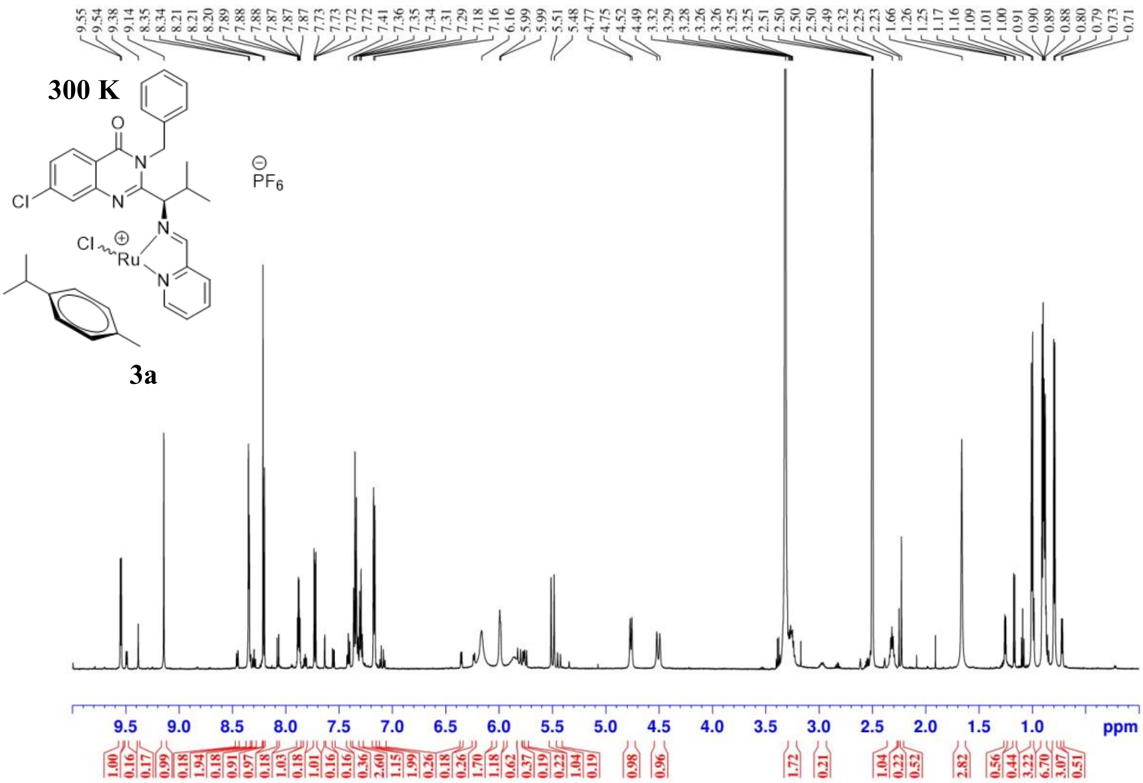

Figure S38. ^{1}H NMR spectra of **3a** in DMSO-d$_6$ at 300 K

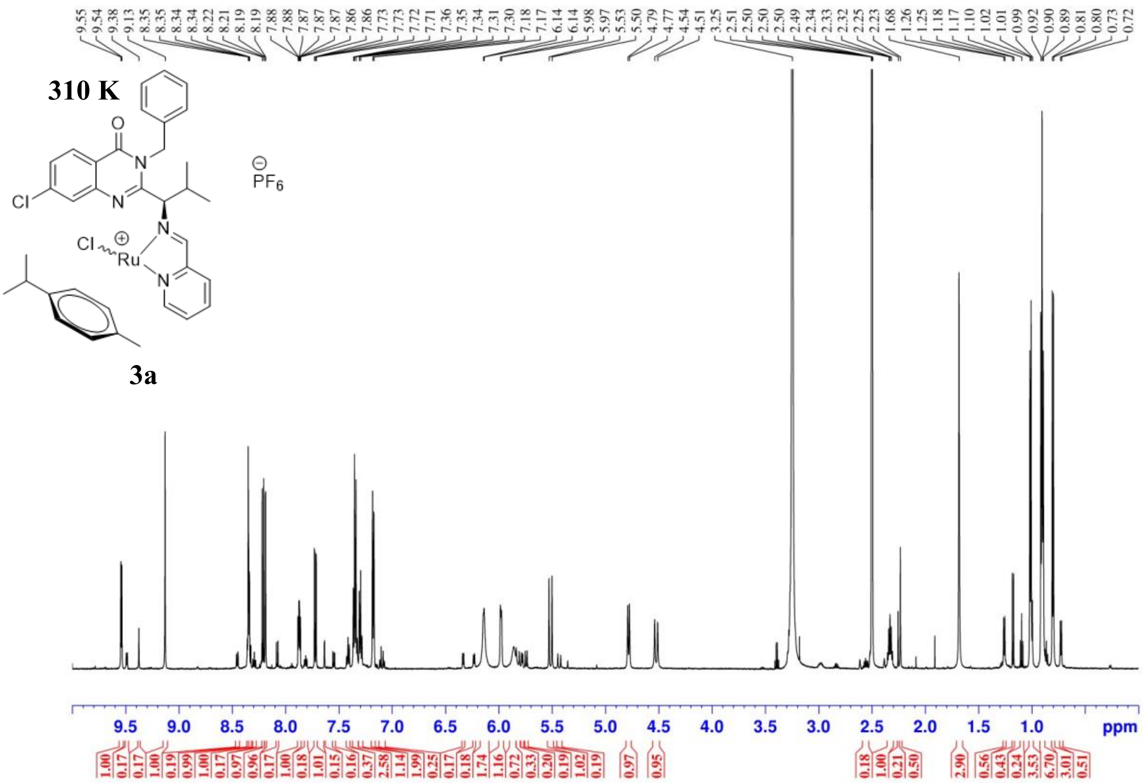

Figure S39. ^{1}H NMR spectra of **3a** in DMSO-d$_6$ at 310 K

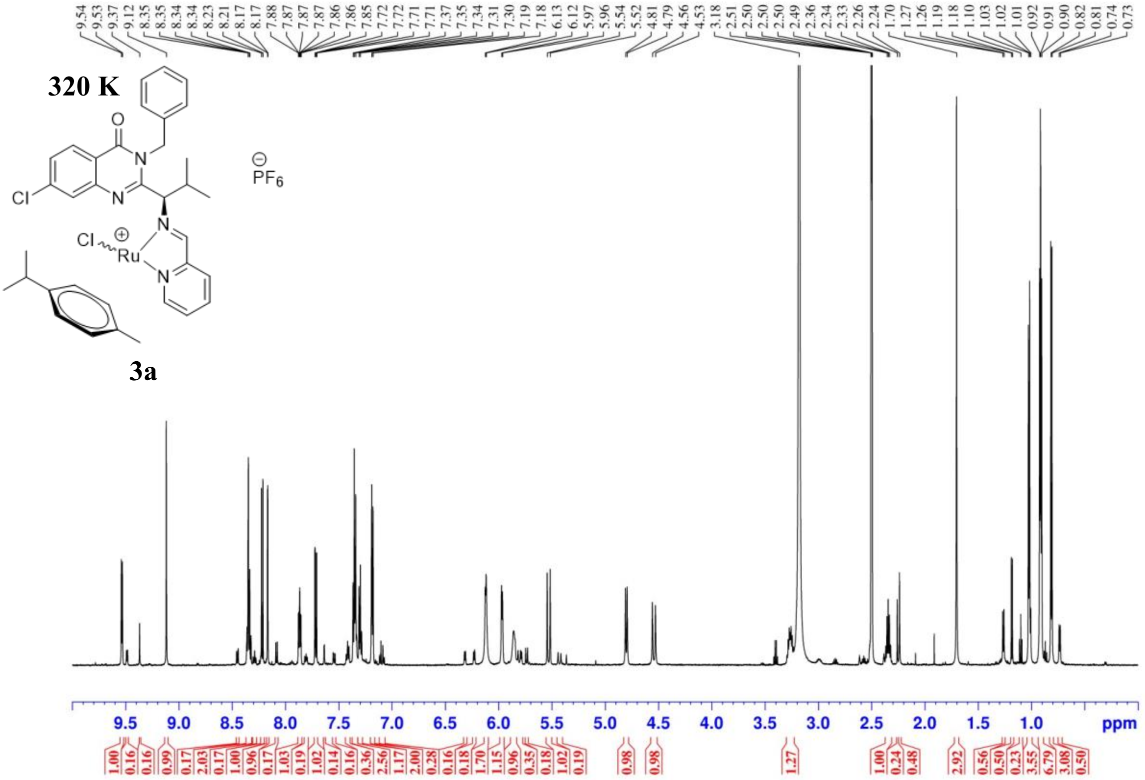

Figure S40. ^{1}H NMR spectra of **3a** in DMSO-d_6 at 320 K

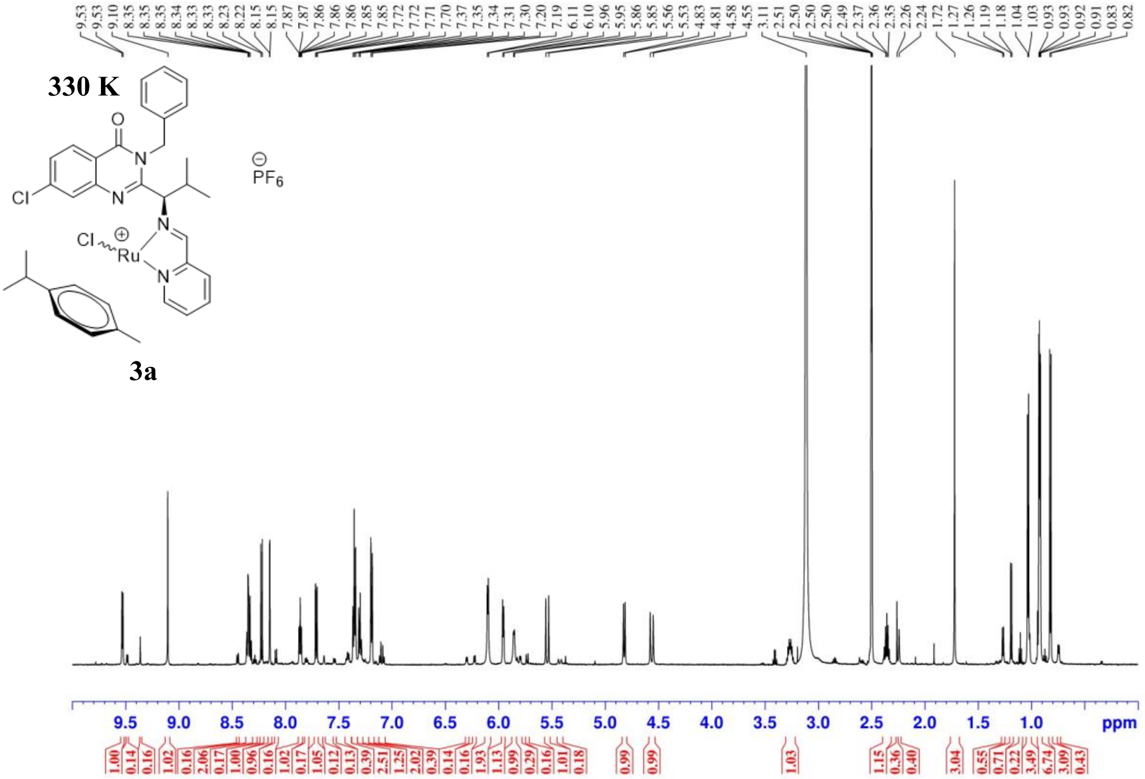

Figure S41. ^{1}H NMR spectra of **3a** in DMSO-d_6 at 330 K

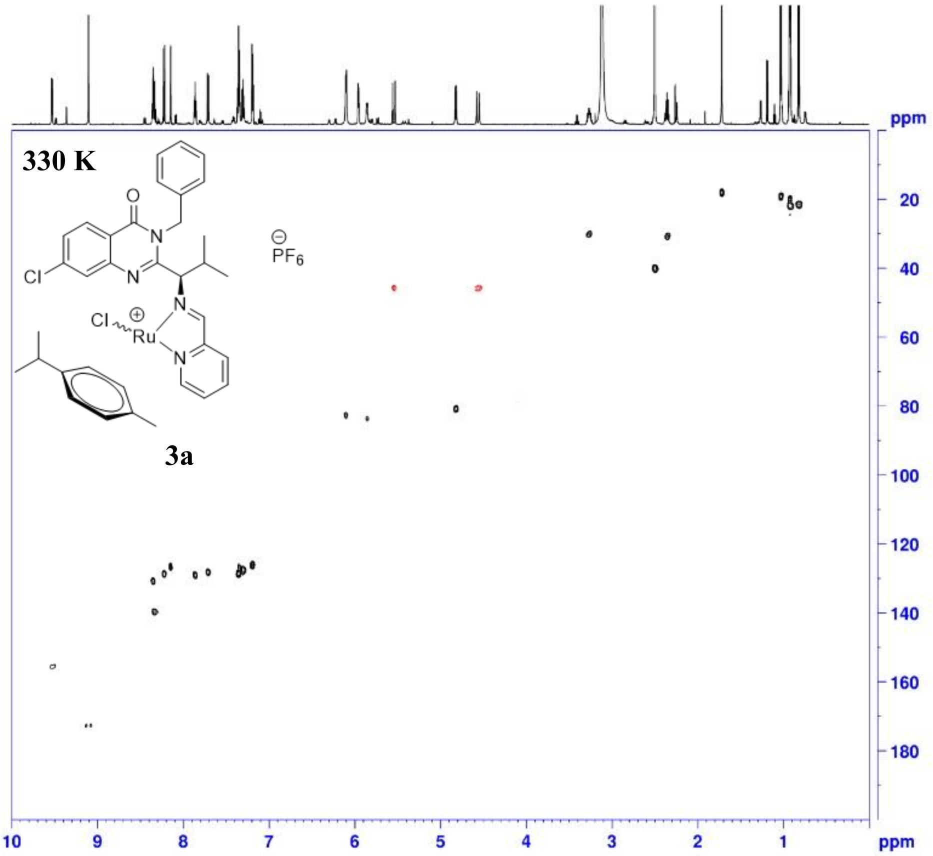

Figure S42. ^{1}H-^{13}C HSQC NMR spectra of **3a** in DMSO-d_6 at 330 K

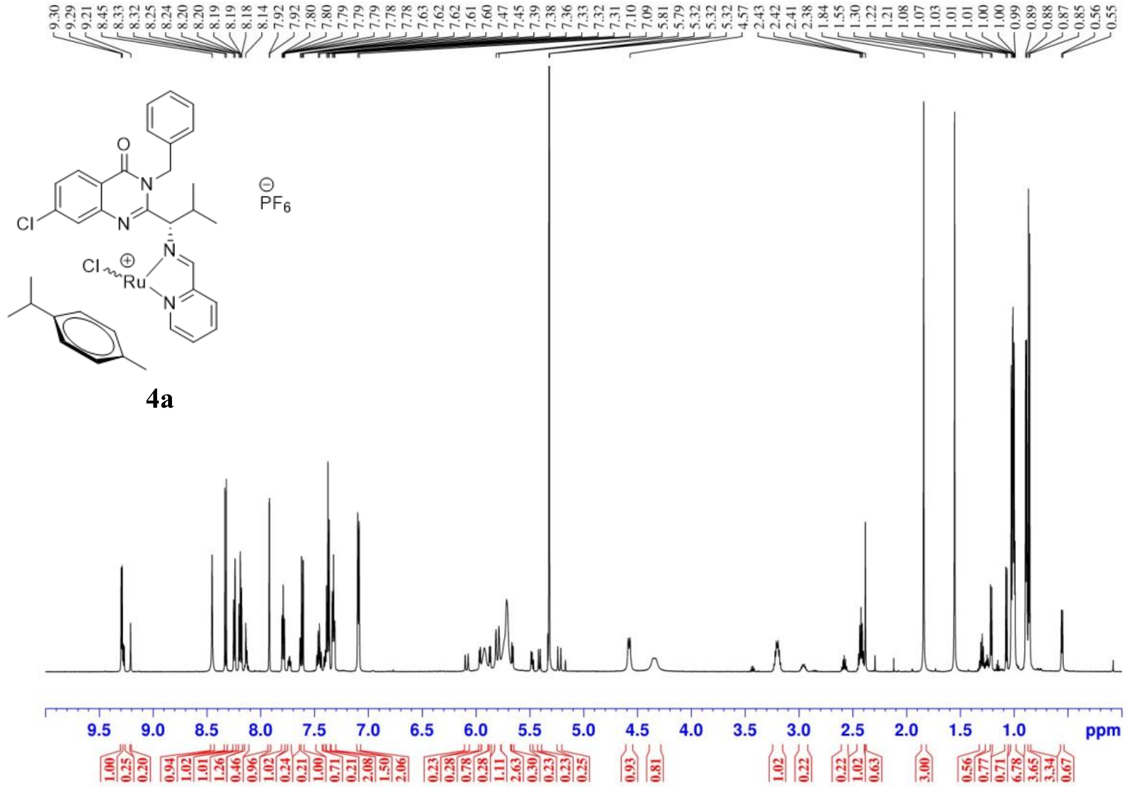

Figure S43. ^{1}H NMR spectra of **4a** in CD_2Cl_2

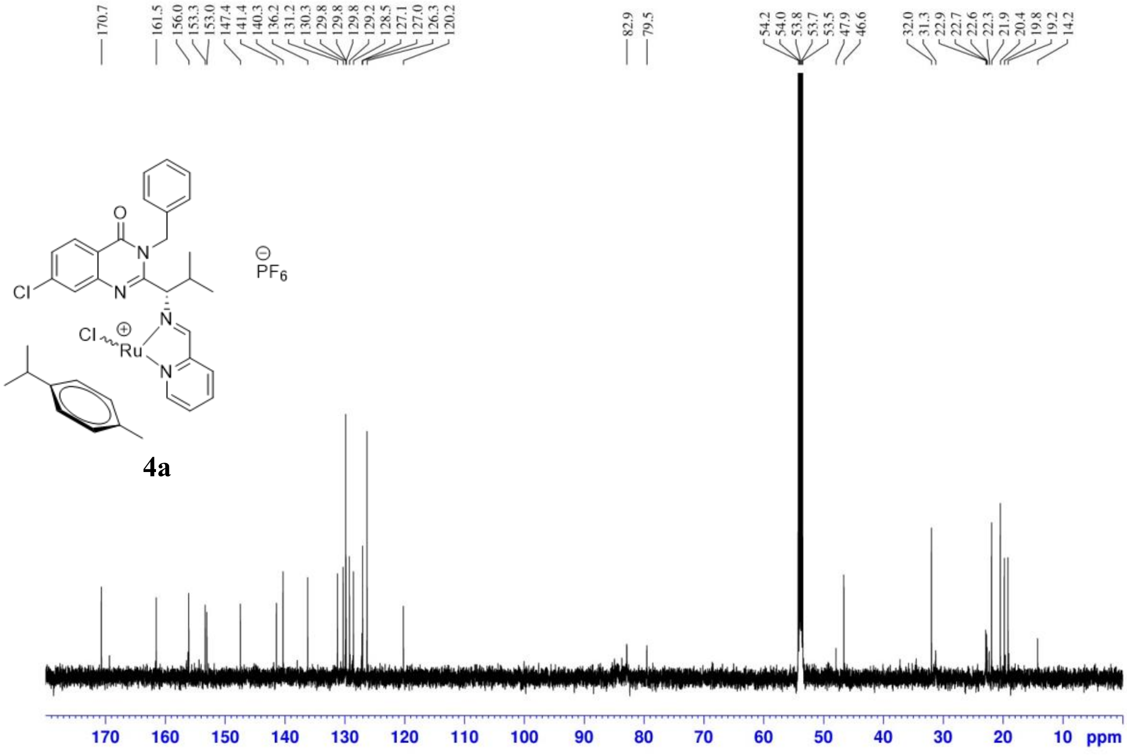

Figure S44. $^{13}C\{^1H\}$ NMR spectra of **4a** in CD_2Cl_2

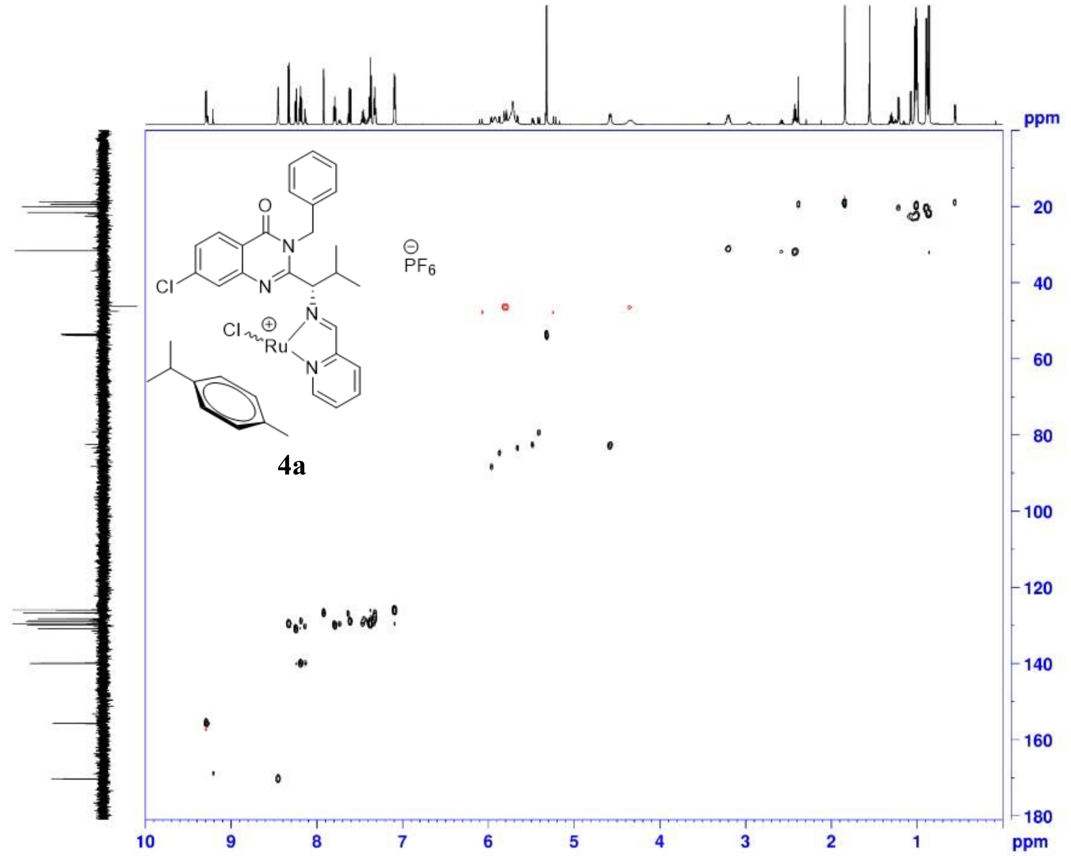

Figure S45. ^{1}H-^{13}C HSQC NMR spectra of **4a** in CD_2Cl_2

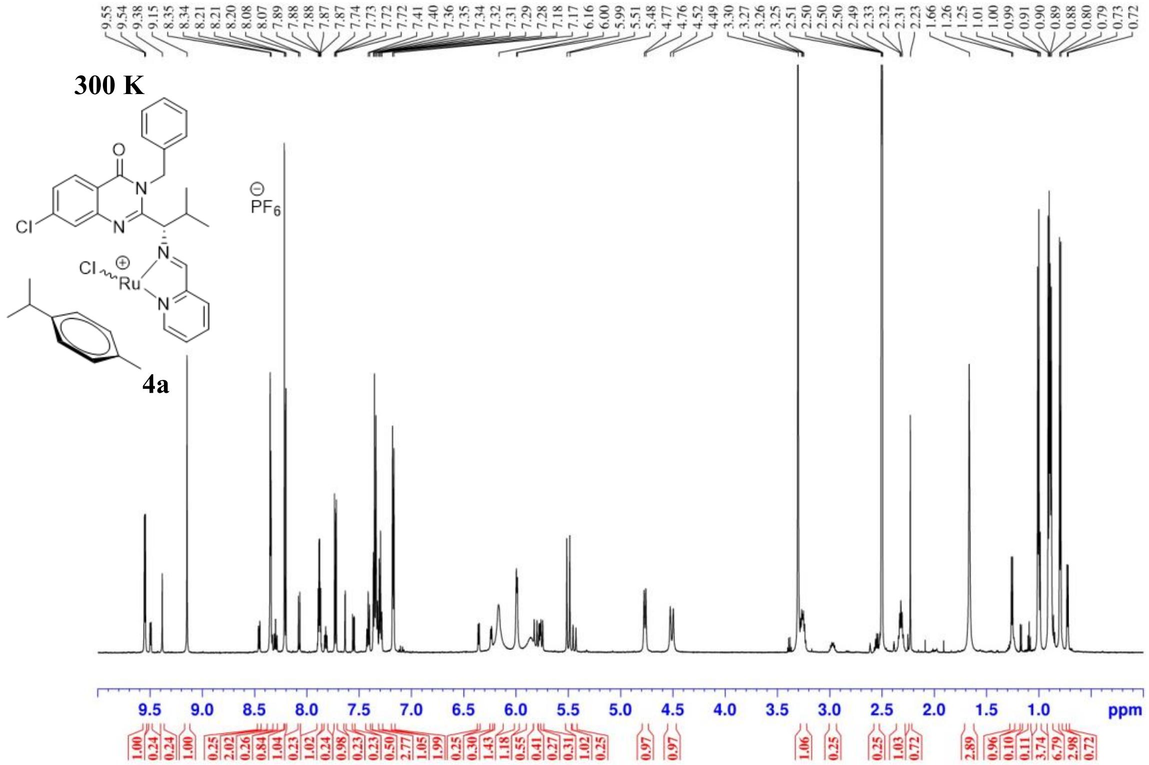

Figure S46. ^{1}H NMR spectra of **4a** in DMSO-d_6 at 300K

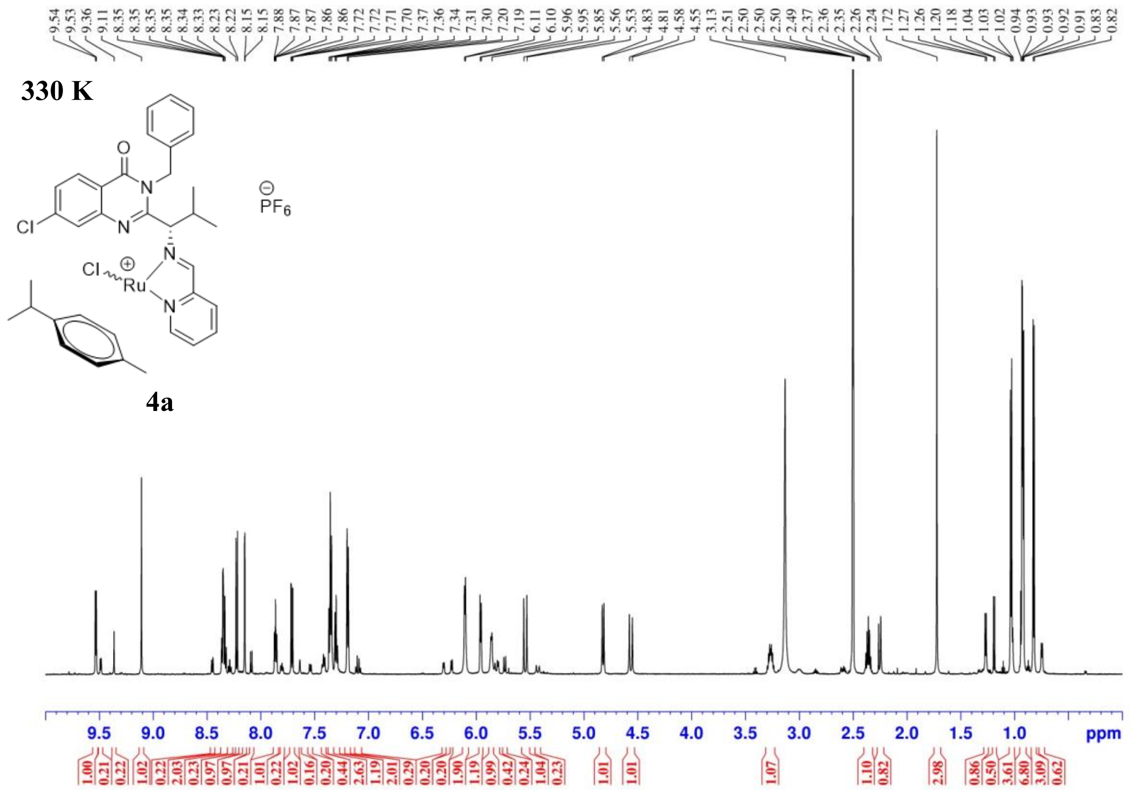

Figure S47. ^{1}H NMR spectra of **4a** in DMSO-d_6 at 330K

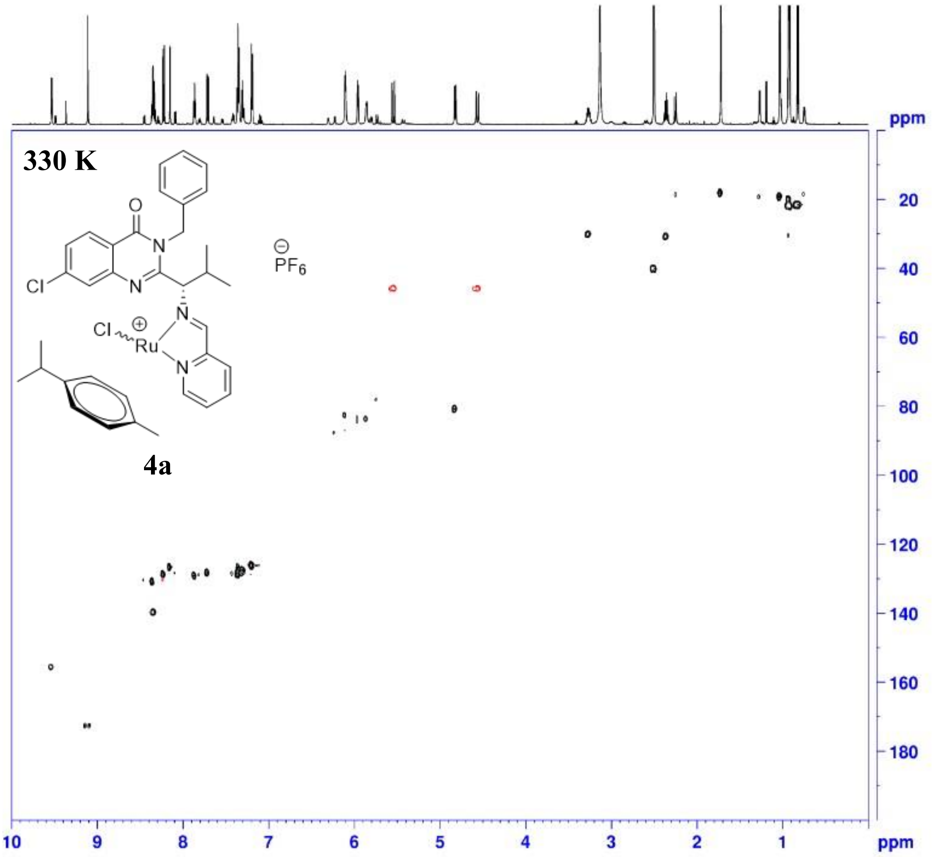

Figure S48. ^{1}H-^{13}C HSQC NMR spectra of **4a** in DMSO-d_6 at 330K

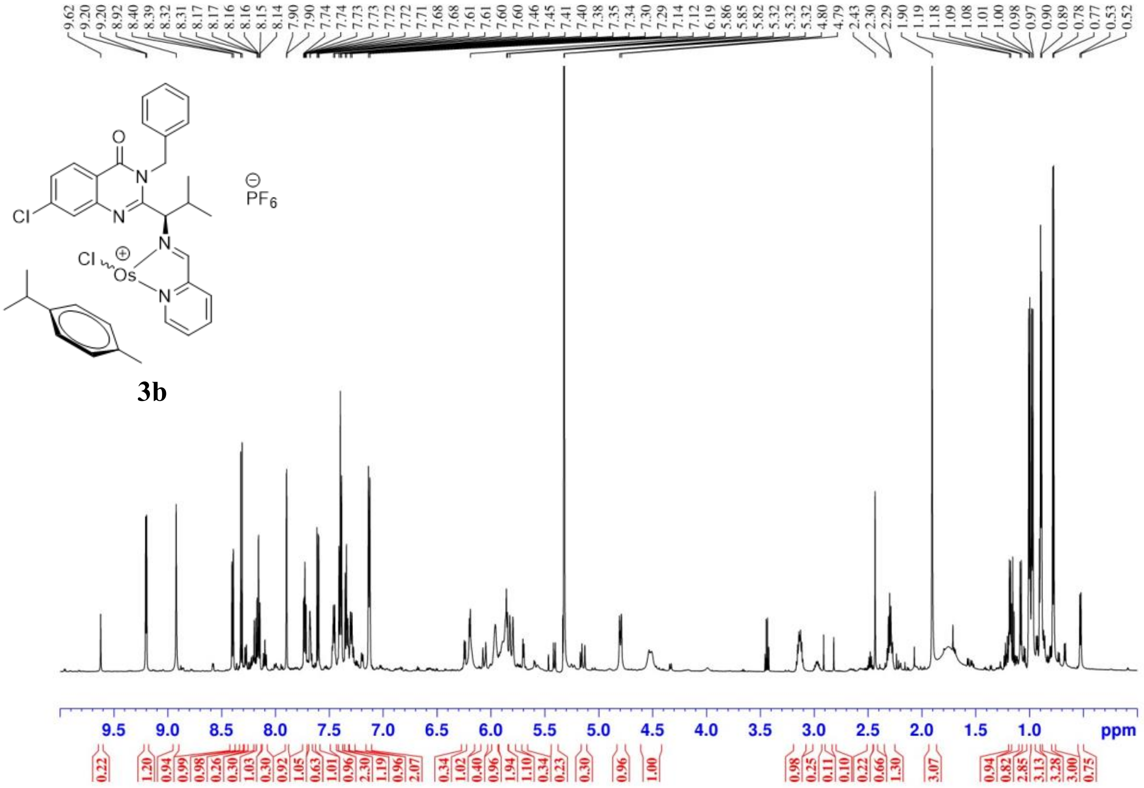

Figure S49. ^{1}H NMR spectra of **3b** in CD_2Cl_2

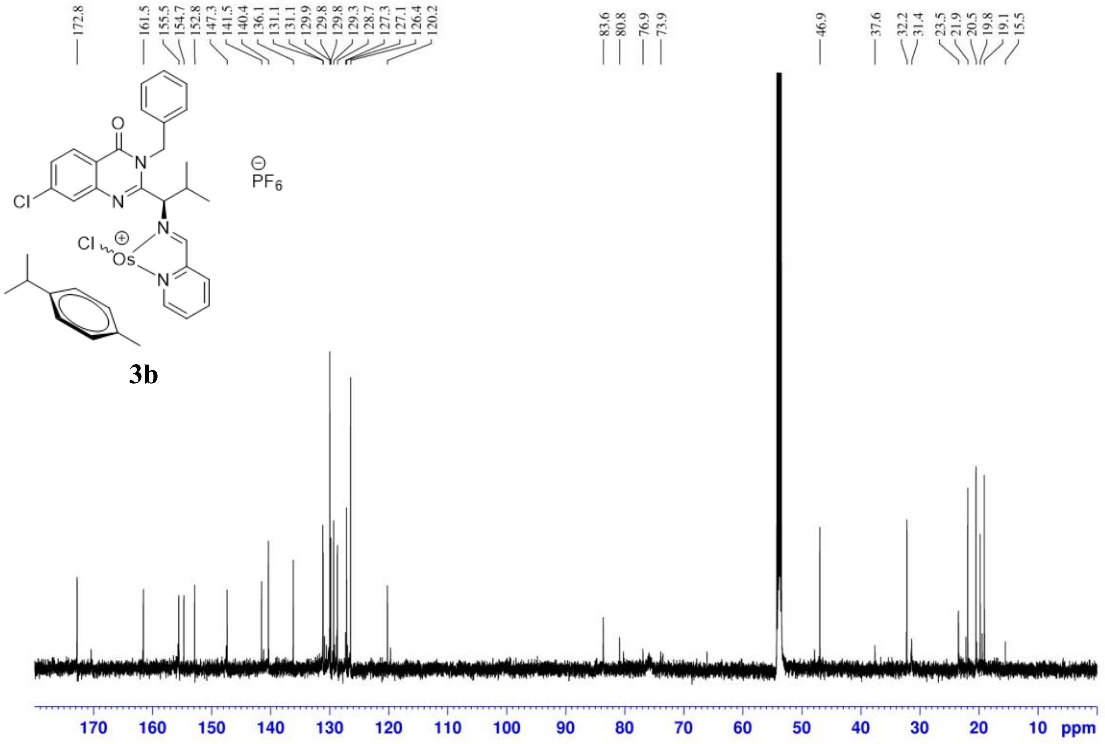

Figure S50. $^{13}C\{^{1}H\}$ NMR spectra of **3b** in CD_2Cl_2

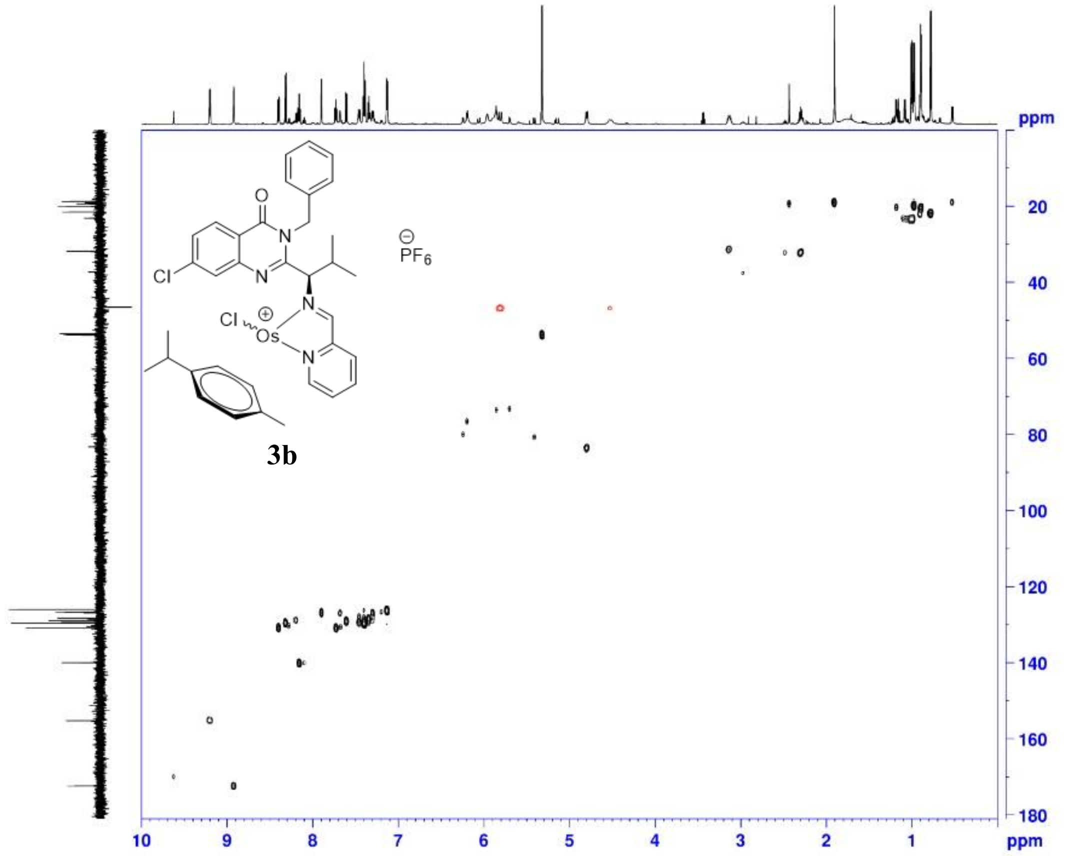

Figure S51. ^{1}H-^{13}C HSQC NMR spectra of **3b** in CD_2Cl_2

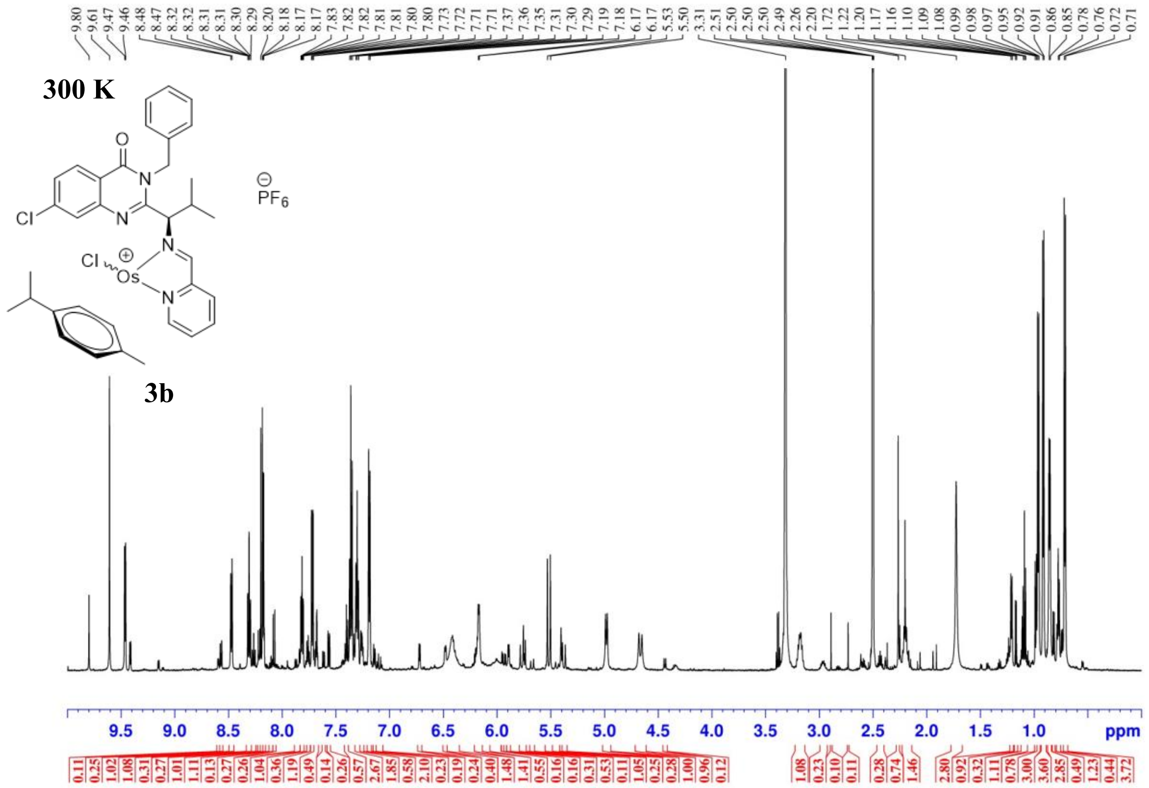

Figure S52. ^{1}H NMR spectra of **3b** in DMSO-d_6 at 300 K

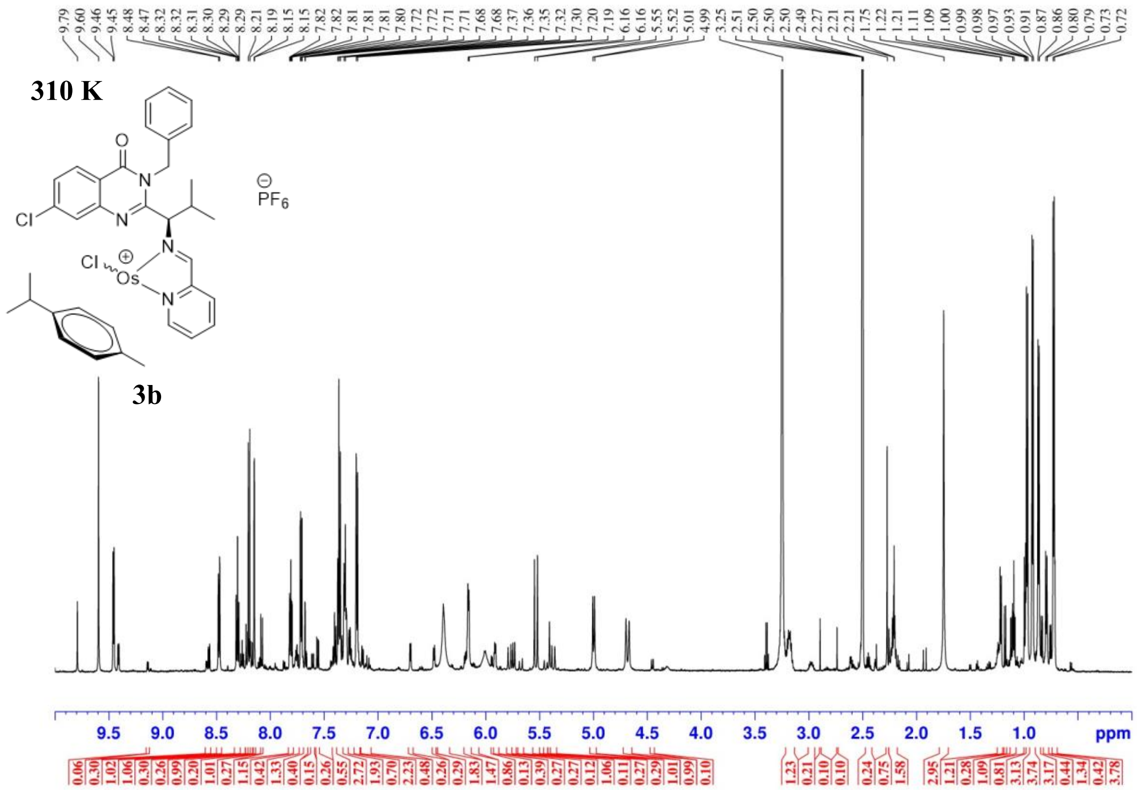

Figure S53. ^{1}H NMR spectra of **3b** in DMSO-d_6 at 310 K

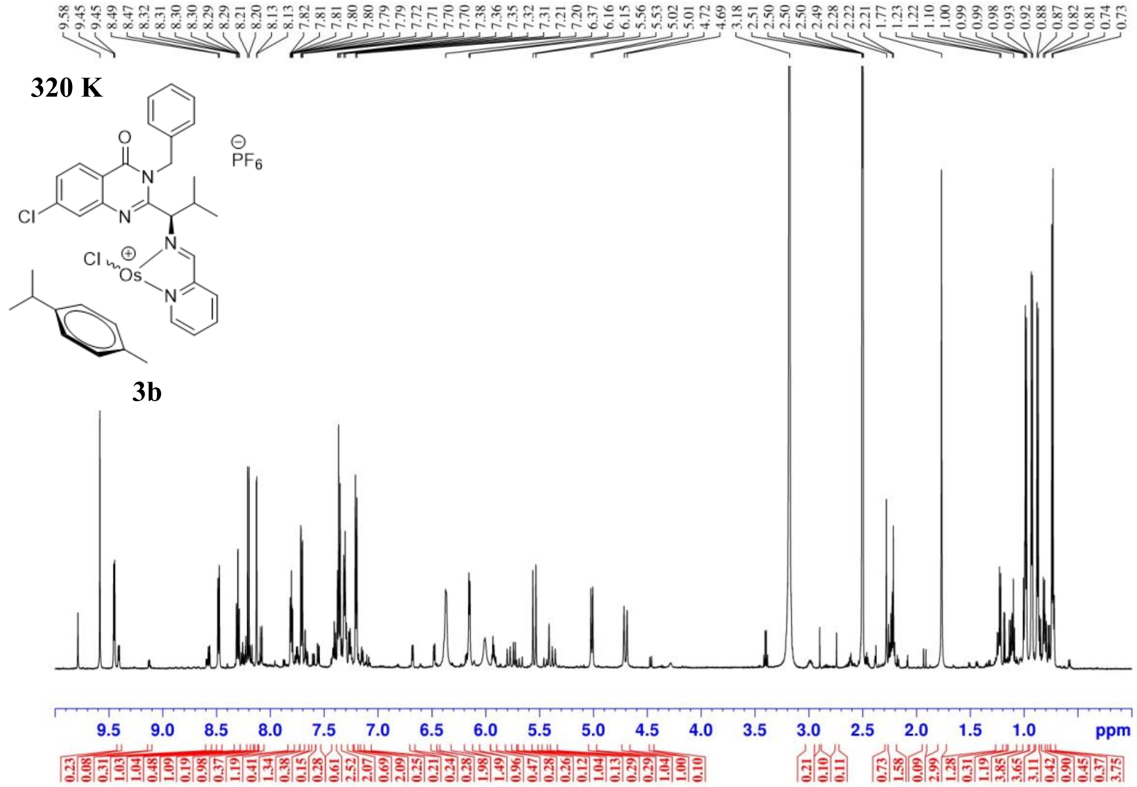

Figure S54. ^{1}H NMR spectra of **3b** in DMSO-d_6 at 320 K

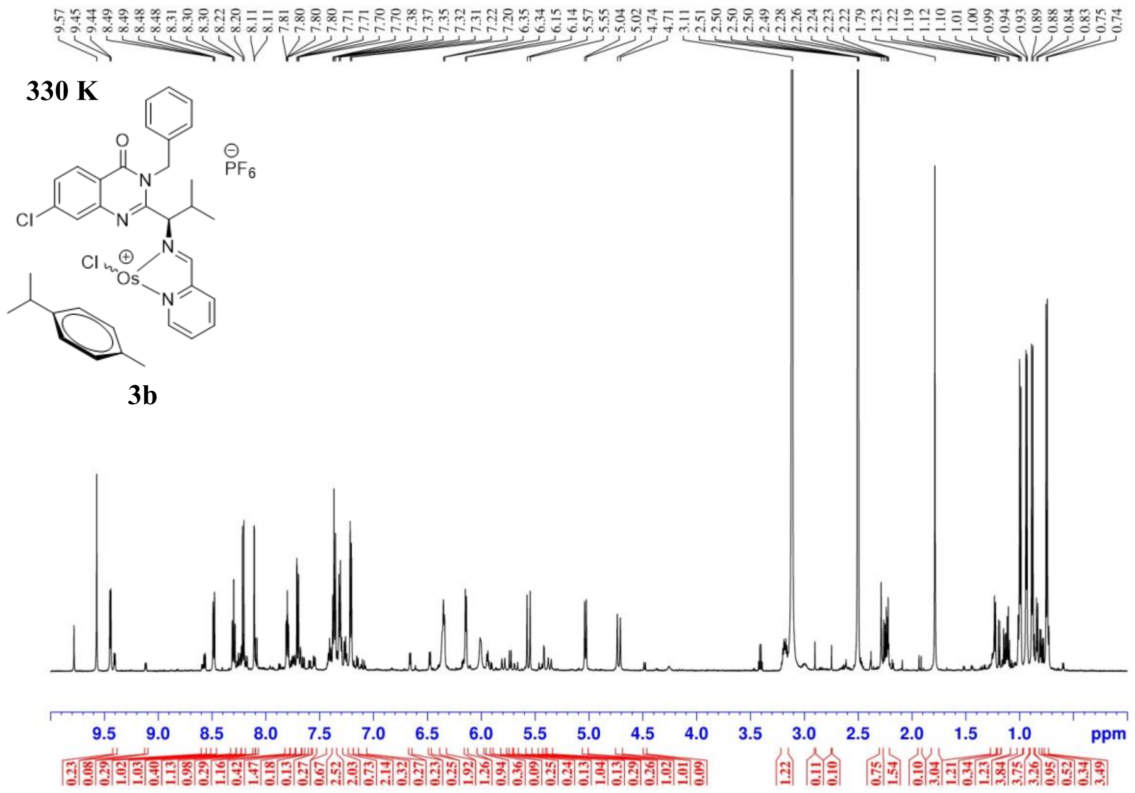

Figure S55. ^{1}H NMR spectra of **3b** in DMSO-d$_6$ at 330 K

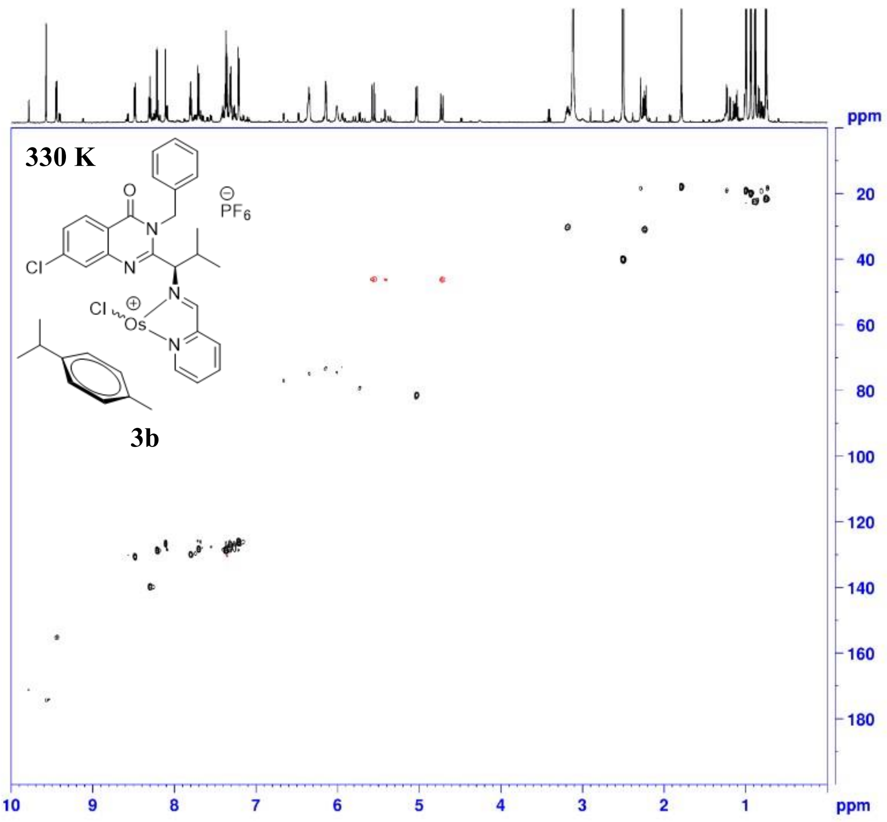

Figure S56. ^{1}H-^{13}C HSQC NMR spectra of **3b** in DMSO-d$_6$ at 330 K

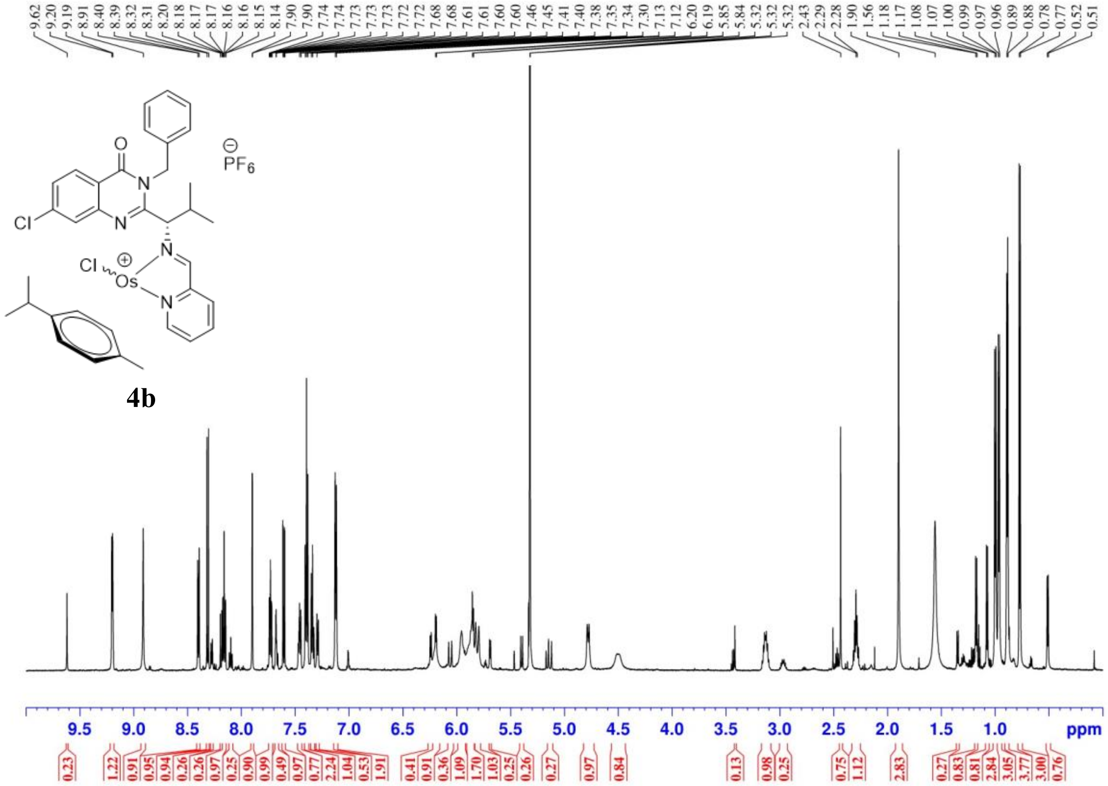

Figure S57. ^{1}H NMR spectra of **4b** in CD_2Cl_2

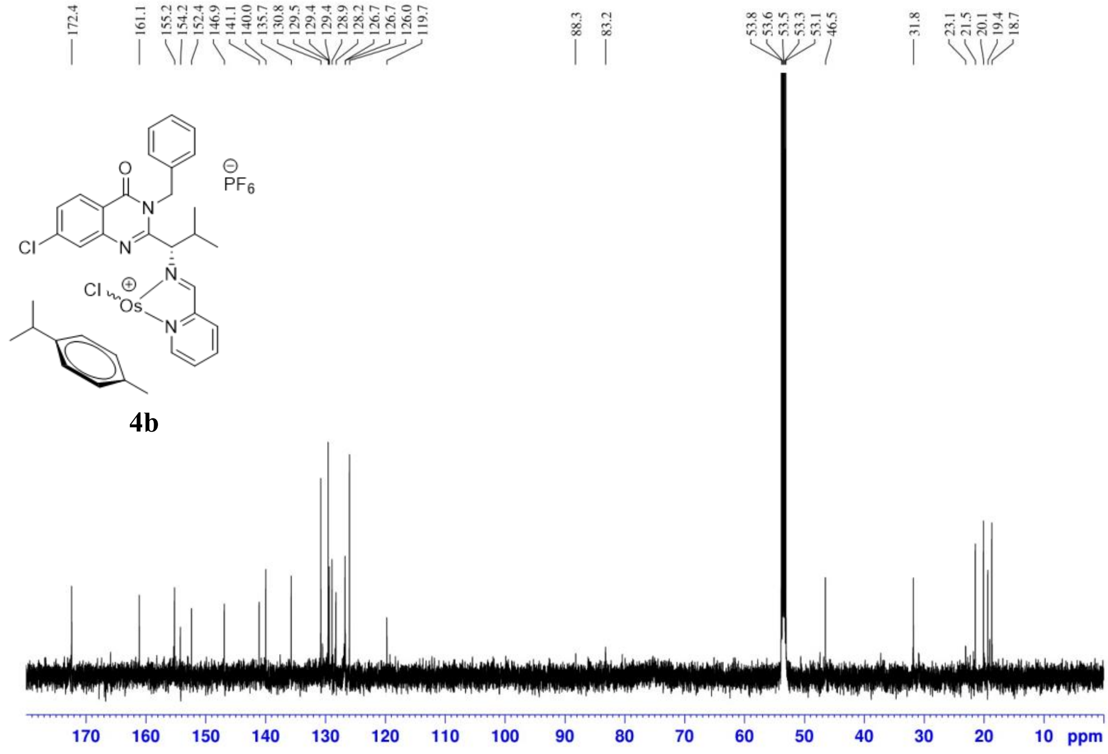

Figure S58. $^{13}C\{^{1}H\}$ NMR spectra of **4b** in CD_2Cl_2

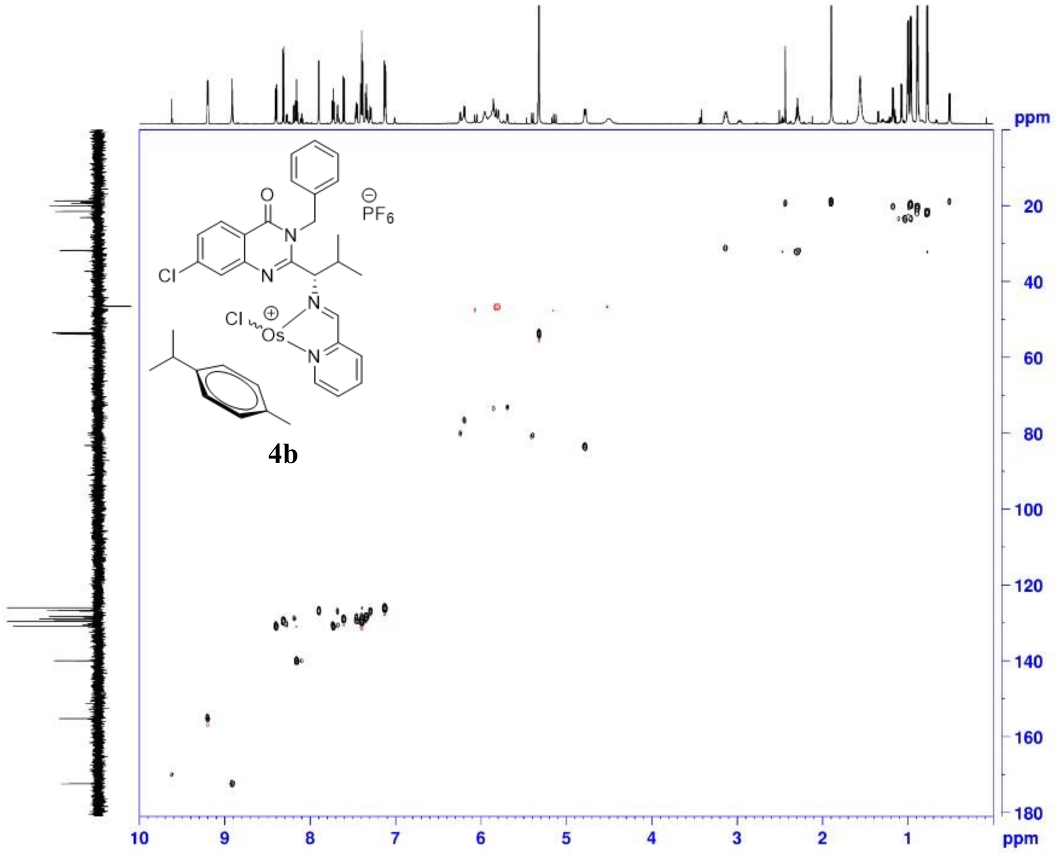

Figure S59. ^{1}H-^{13}C HSQC NMR spectra of **4b** in CD_2Cl_2

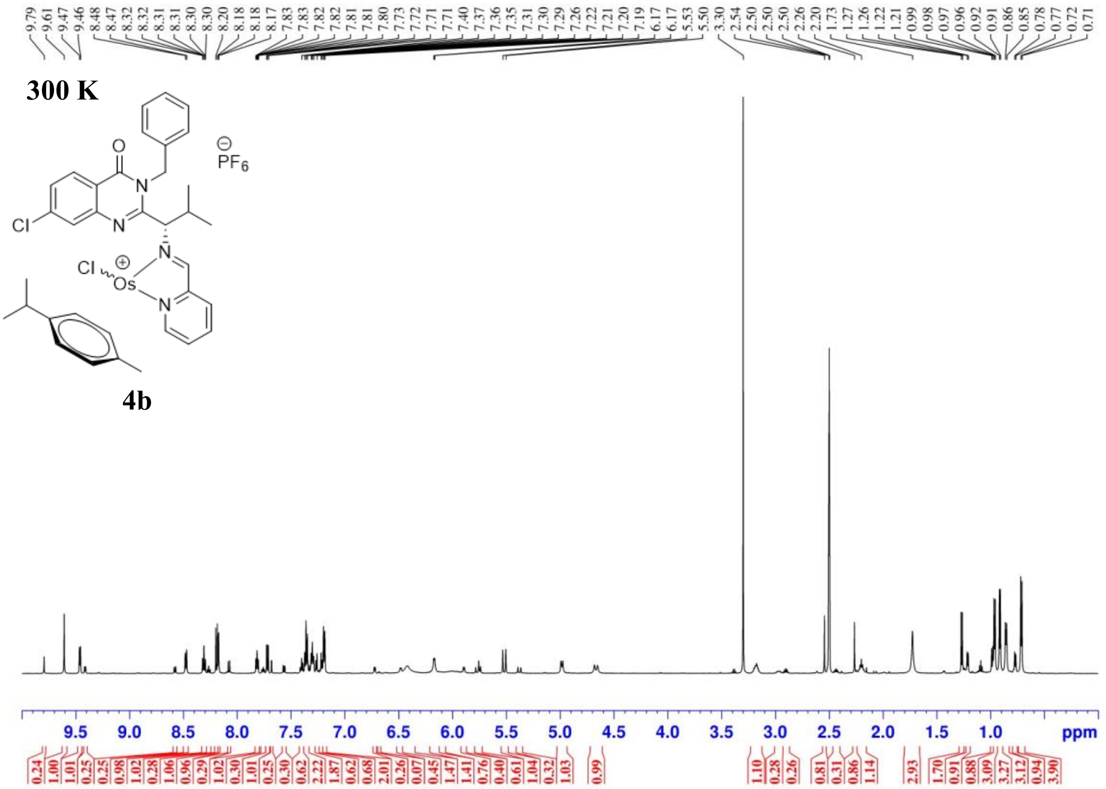

Figure S60. ^{1}H NMR spectra of 4**b** in DMSO-d_6 at 300 K

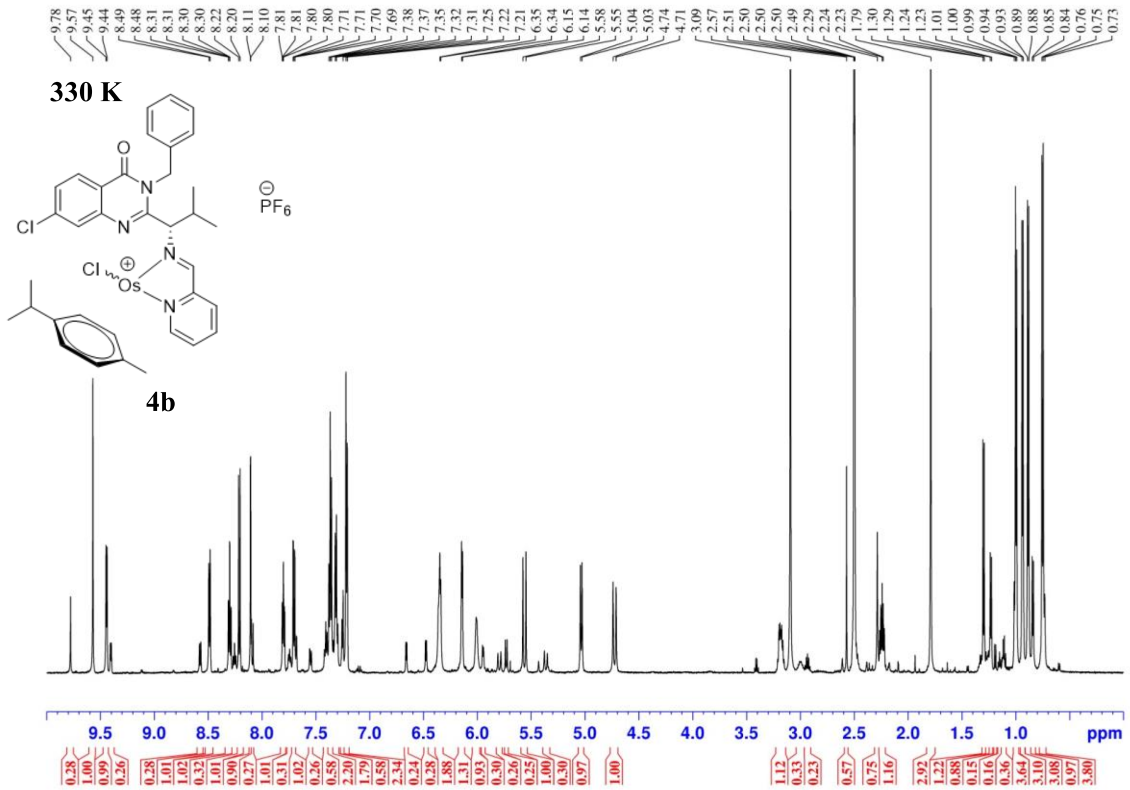

Figure S61. ^{1}H NMR spectra of 4**b** in DMSO-d$_6$ at 330 K

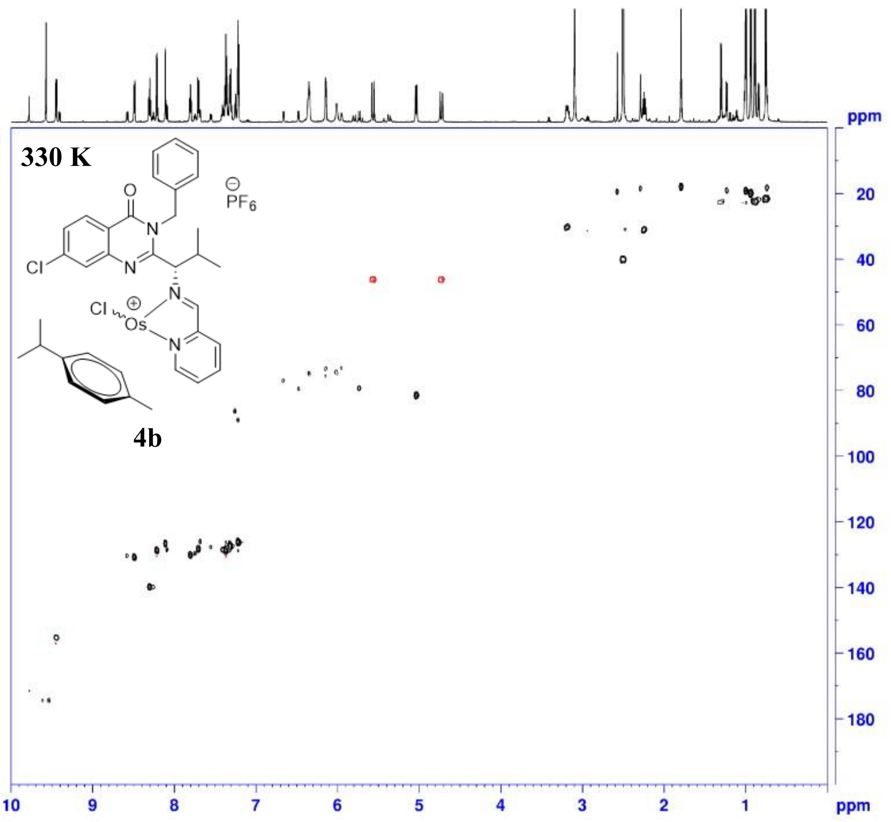

Figure S62. ^{1}H-^{13}C HSQC NMR spectra of **4b** in DMSO-d$_6$ at 330 K

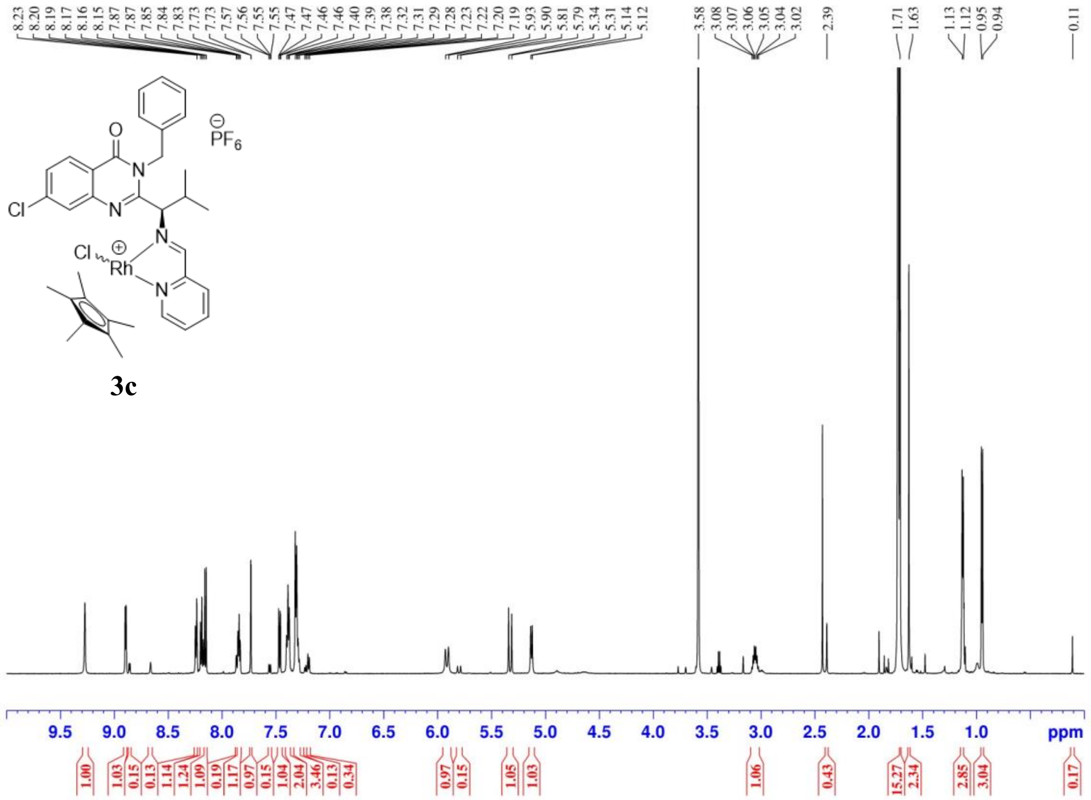

3c

Figure S63. ^{1}H NMR spectra of **3c** in THF-d_8

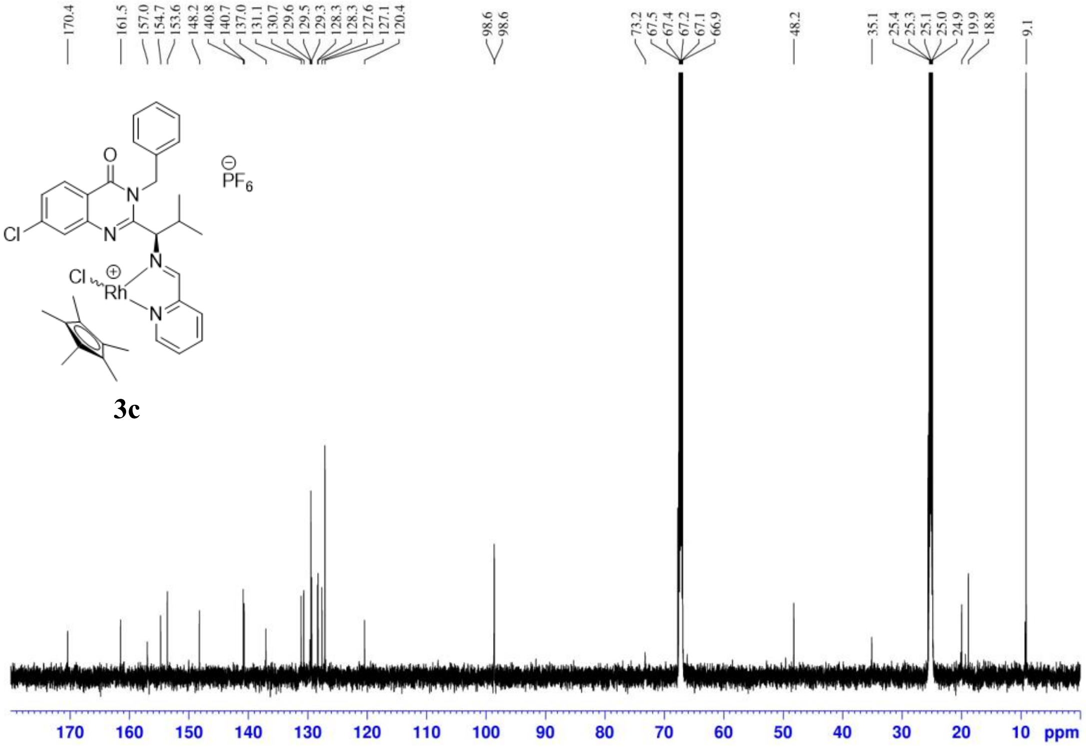

3c

Figure S64. $^{13}C\{^{1}H\}$ NMR spectra of **3c** in THF-d_8

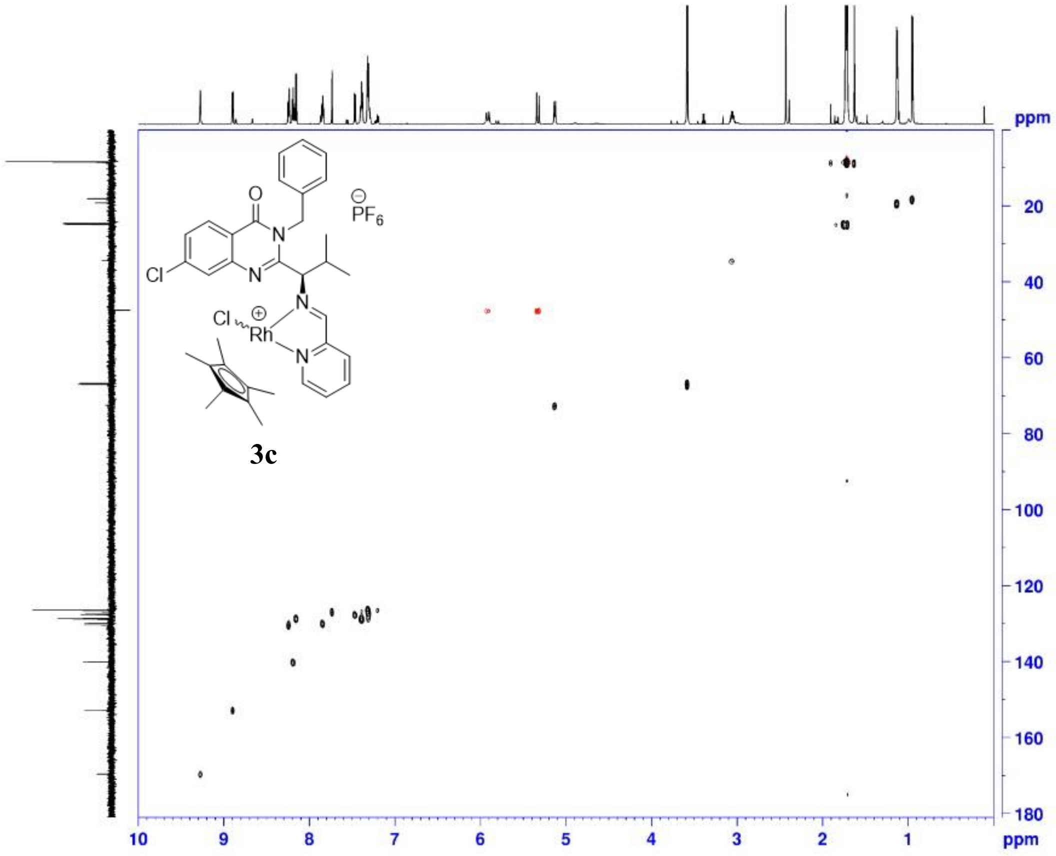

Figure S65. ^{1}H-^{13}C HSQC NMR spectra of **3c** in THF-d_8

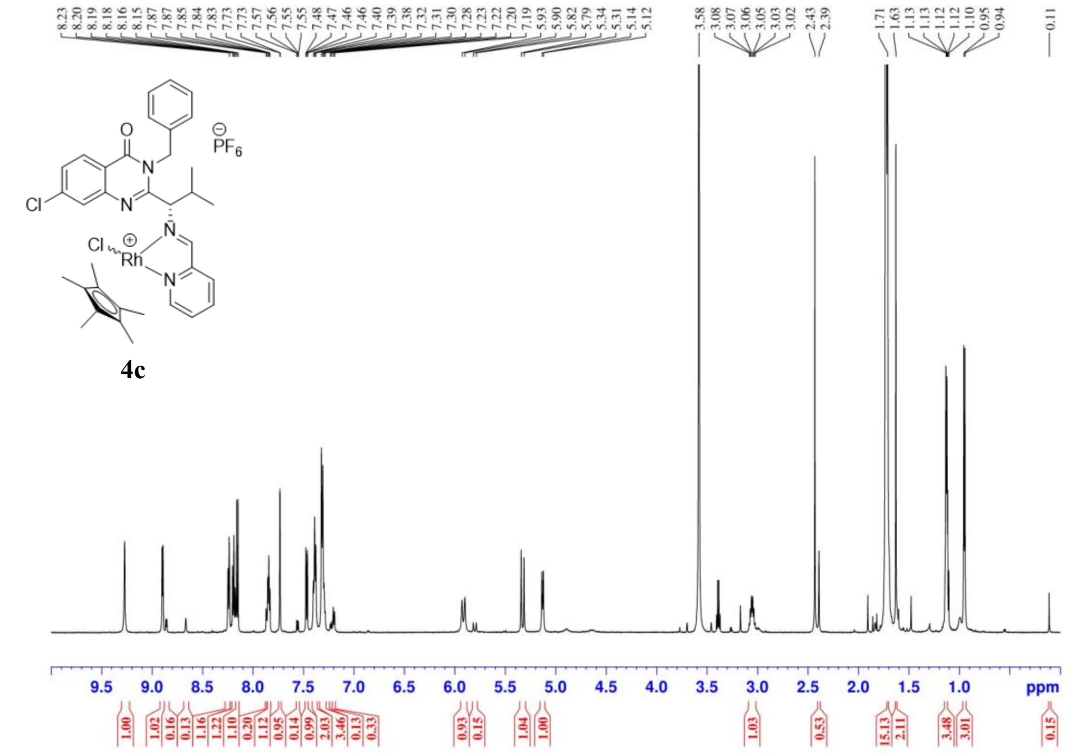

Figure S66. ^{1}H NMR spectra of **4c** in THF-d_8

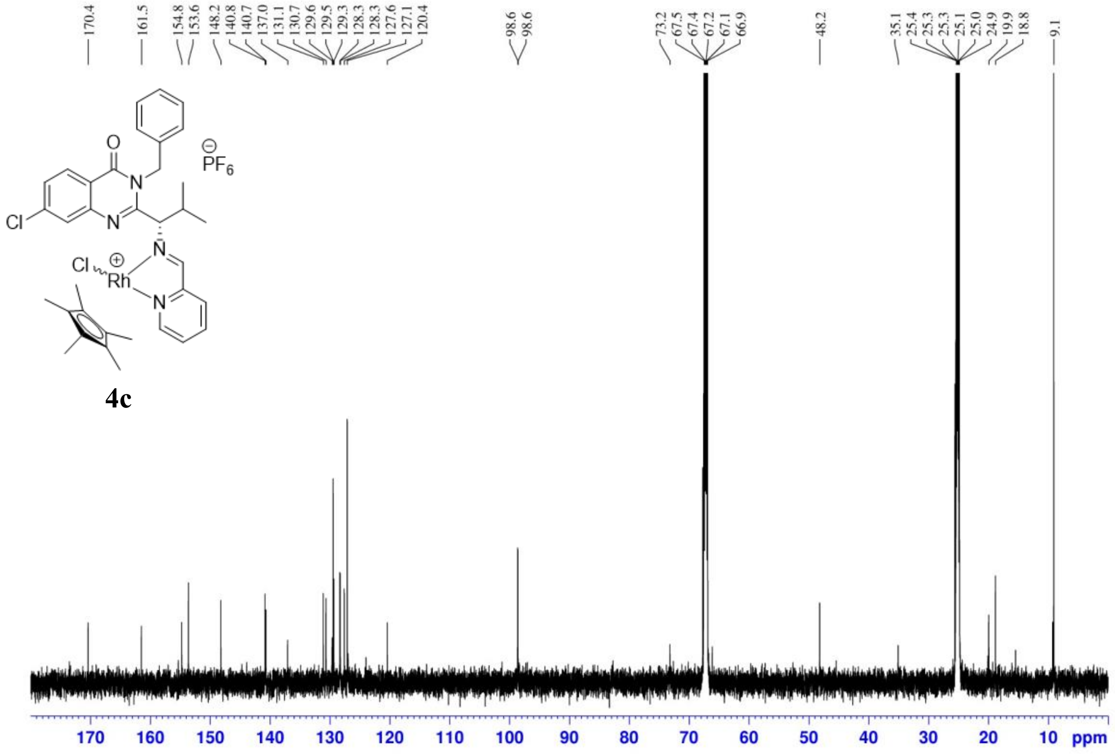

Figure S67. ^{13}C{^{1}H} NMR spectra of **4c** in THF-d_8

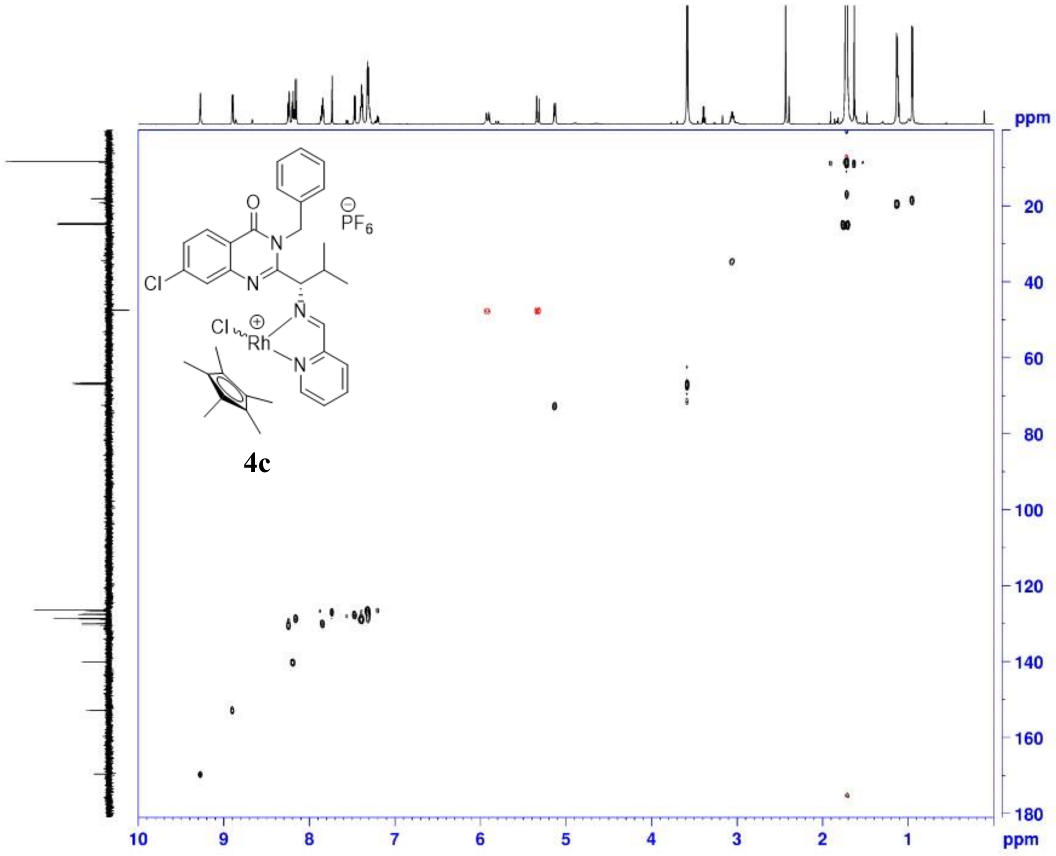

Figure S68. ^{1}H-^{13}C HSQC NMR spectra of **4c** in THF-d_8

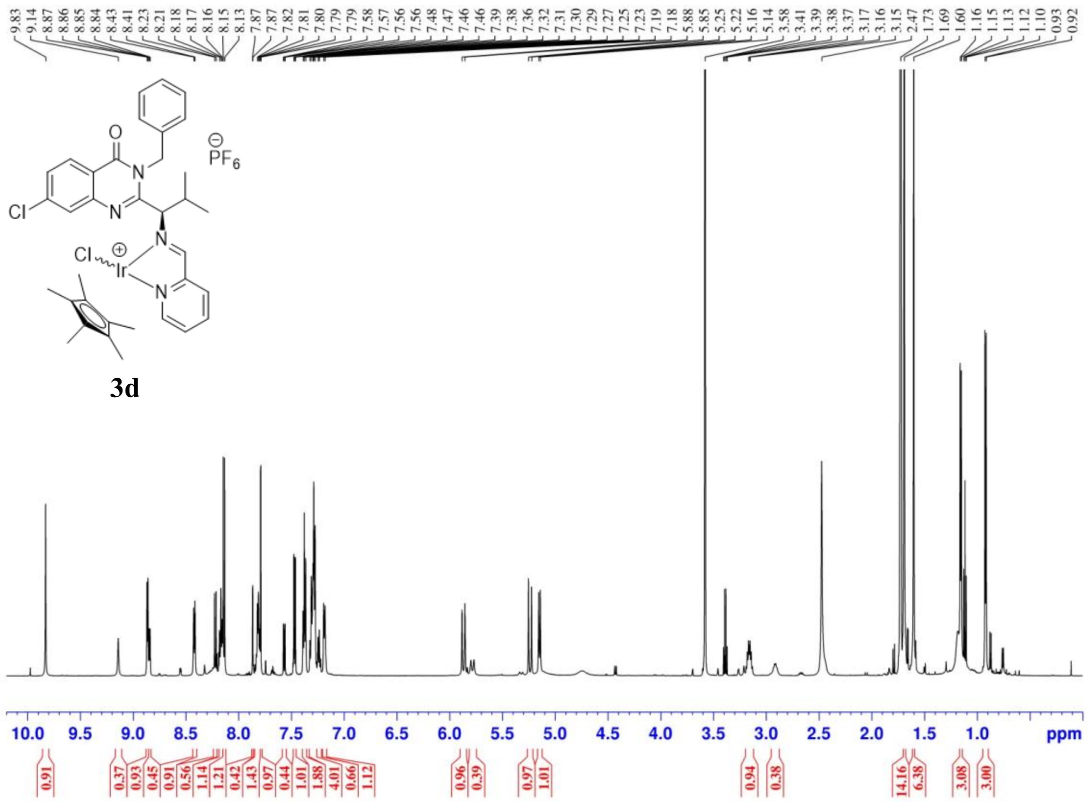

Figure S69. ^{1}H NMR spectra of **3d** in THF-d_8

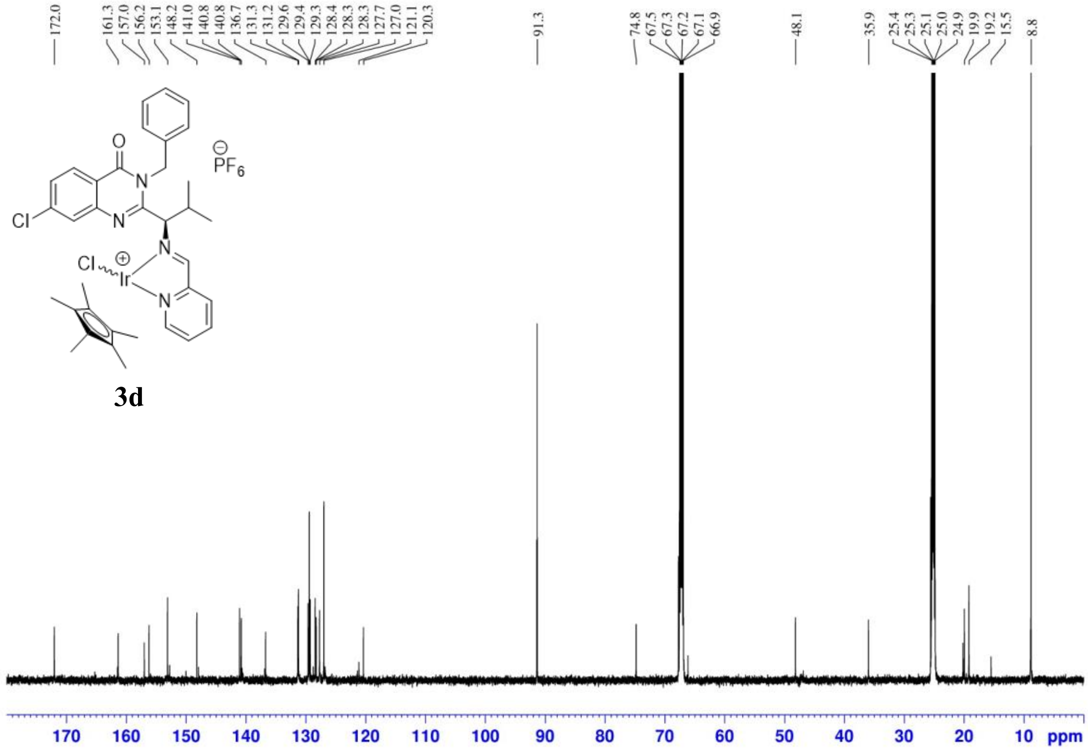

Figure S70. $^{13}C\{^{1}H\}$ NMR spectra of **3d** in THF-d_8

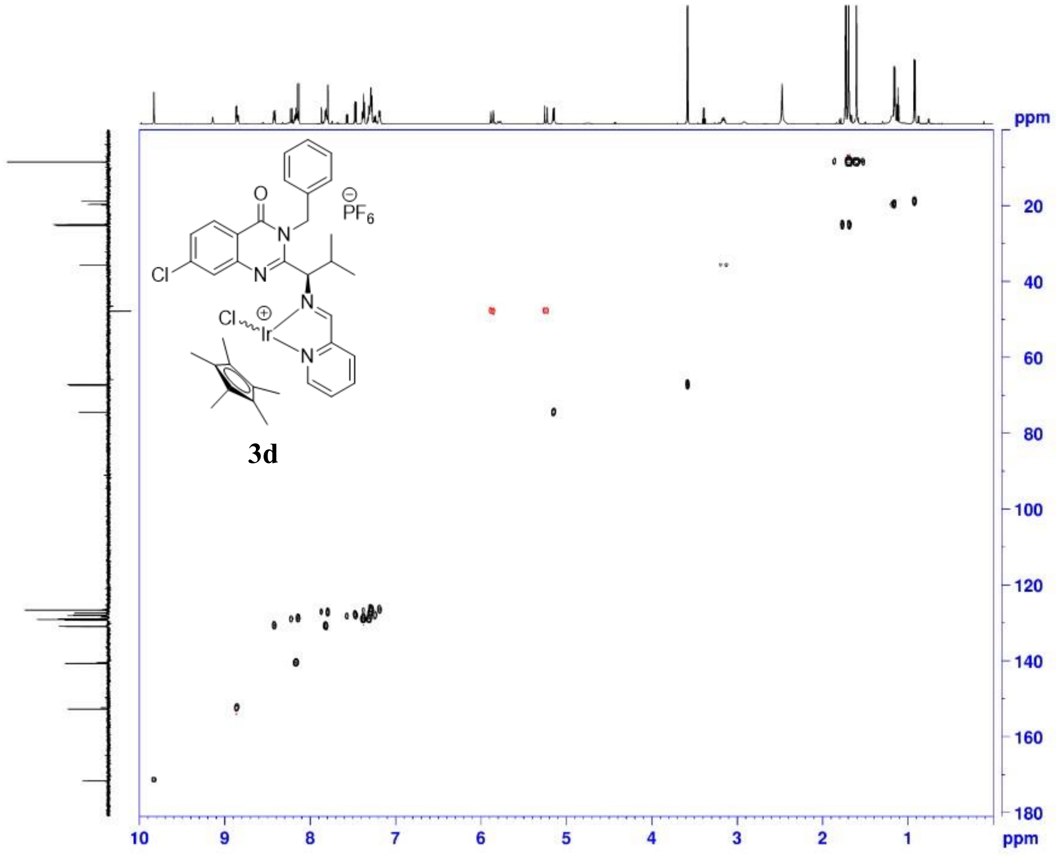

Figure S71. ^{1}H-^{13}C HSQC NMR spectra of **3d** in THF-d_8

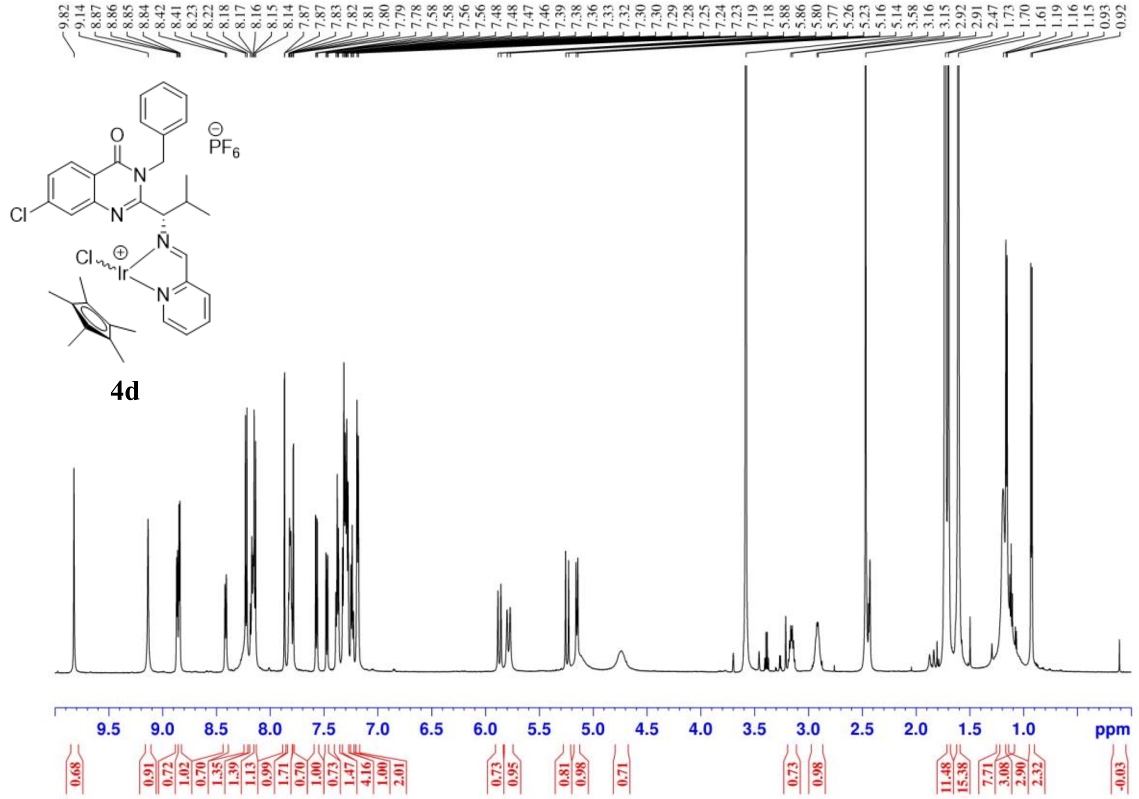

Figure S72. ^{1}H NMR spectra of **4d** in THF-d_8

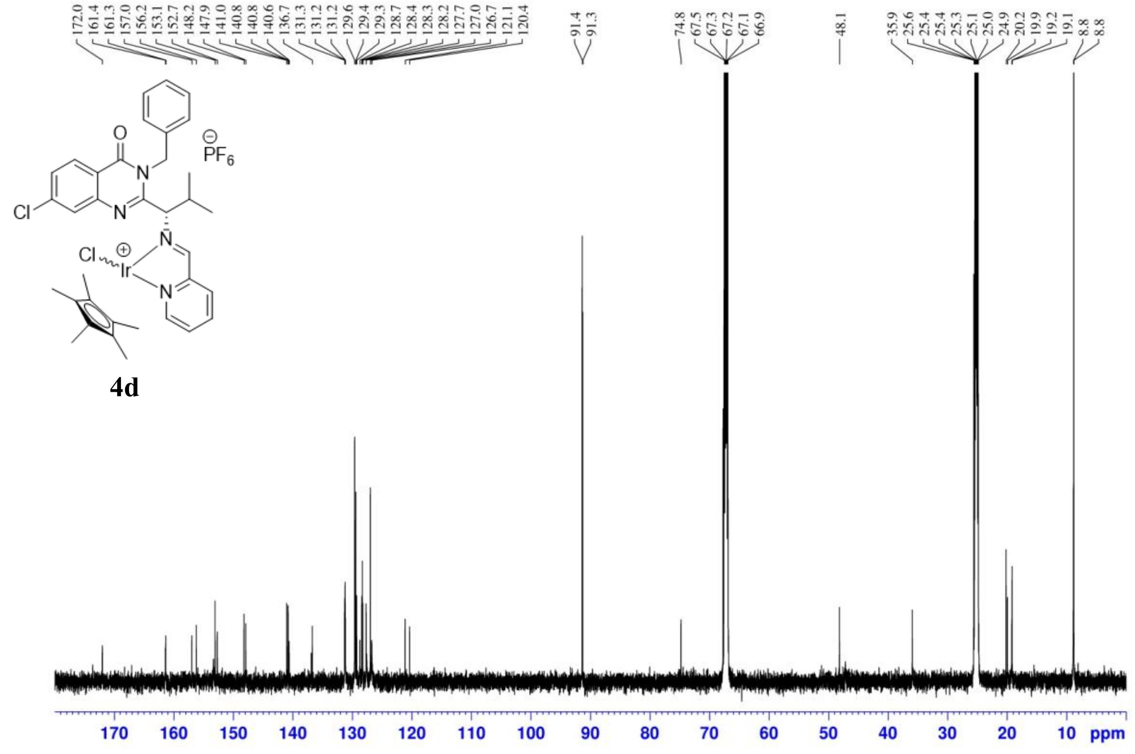

Figure S73. $^{13}C\{^{1}H\}$ NMR spectra of **4d** in THF-d_8

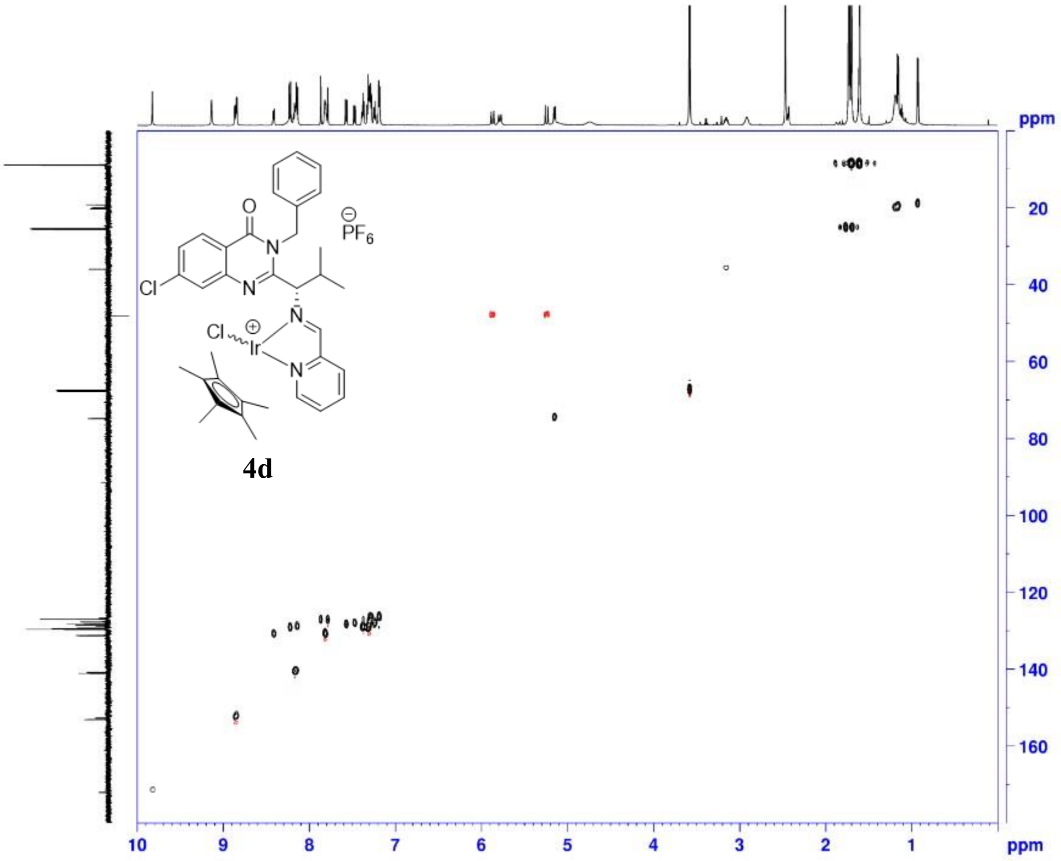

4d

Figure S74. ^{1}H-^{13}C HSQC NMR spectra of **4d** in THF-d_8

Bibliography

(1) Jerschow, A.; Müller, N. 3D Diffusion-Ordered TOCSY for Slowly Diffusing Molecules. *J. Magn. Reson., Ser. A* **1996**, *123* (2), 222-225. DOI: https://doi.org/10.1006/jmra.1996.0241.
(2) Jerschow, A.; Müller, N. Suppression of Convection Artifacts in Stimulated-Echo Diffusion Experiments. Double-Stimulated-Echo Experiments. *J. Magn. Reson.* **1997**, *125* (2), 372-375. DOI: https://doi.org/10.1006/jmre.1997.1123.
(3) Xcalibur/SuperNova CCD System, CrysAlisPro Software System, Rigaku Oxford Diffraction (2020).
(4) Flack, H. On enantiomorph-polarity estimation. *Acta Cryst. A* **1983**, *39* (6), 876-881. DOI: doi:10.1107/S0108767383001762.
(5) Sheldrick, G. SHELXT - Integrated space-group and crystal-structure determination. *Acta Cryst. A* **2015**, *71* (1), 3-8. DOI: doi:10.1107/S2053273314026370.
(6) Sheldrick, G. Crystal structure refinement with SHELXL. *Acta Cryst. C* **2015**, *71* (1), 3-8. DOI: doi:10.1107/S2053229614024218.
(7) Macrae, C. F.; Bruno, I. J.; Chisholm, J. A.; Edgington, P. R.; McCabe, P.; Pidcock, E.; Rodriguez-Monge, L.; Taylor, R.; van de Streek, J.; Wood, P. A. Mercury CSD 2.0 - new features for the visualization and investigation of crystal structures. *J. Appl. Crystallogr.* **2008**, *41* (2), 466-470. DOI: doi:10.1107/S0021889807067908.

(8) Bruno, I. J.; Cole, J. C.; Kessler, M.; Luo, J.; Motherwell, W. D. S.; Purkis, L. H.; Smith, B. R.; Taylor, R.; Cooper, R. I.; Harris, S. E.; et al. Retrieval of Crystallographically-Derived Molecular Geometry Information. *J. Chem. Inf. Comput.* **2004**, *44* (6), 2133-2144. DOI: 10.1021/ci049780b.
(9) Spek, A. checkCIF validation ALERTS: what they mean and how to respond. *Acta Cryst. E* **2020**, *76* (1), 1-11. DOI: doi:10.1107/S2056989019016244.
